# Acknowledgment to the Reviewers of *JCM* in 2022

**DOI:** 10.3390/jcm12030782

**Published:** 2023-01-18

**Authors:** JCM Editorial Office

**Affiliations:** MDPI AG, St. Alban-Anlage 66, 4052 Basel, Switzerland

High-quality academic publishing is built on rigorous peer review. *JCM* was able to uphold its high standards for published papers due to the outstanding efforts of our reviewers. Thanks to the efforts of our reviewers in 2022, the median time to first decision was 20 days and the median time to publication was 41 days. Regardless of whether the articles they examined were ultimately published, the editors would like to express their appreciation and thank the following reviewers for the time and dedication that they have shown *JCM*:
A. Darise FarrisKornelia Kuźnik-TrochaA. J. HoubenKornelia ZarębaAalap ChokshiKory ZimneyAarno DietzKosho YamanouchiAaron Blandino OrtizKosmas SarafidisAaron C. SpauldingKossara DrenovskaAaron PinkhasovKostas LasithiotakisAaron PlittKosuke YoshidaAashima DabasKota BokudaAbbas OrandKotaro HattaAbbas PakdelKottil W. RammohanAbbey SchepersKouji MiyazakiAbbirami ElangovanKouki OhtsukaAbdalkarem AlsharariKoulla ParpaAbdallah Al-SalamehKoumantakis GeorgeAbdelmotagaly ElgaladKourosh HooshmandAbdelouahab BellouKoutlianos NikolaosAbdelrahman M. ElhusseinyKovy Arteaga-LiviasAbderraouf HilaliKranti BhavanaAbdolhassan KazemiKrešimir CrnogaćaAbdorreza Naser-MoghadasiKresimir DolicAbdou ElhendyKrešimir RotimAbdul Alim Al-BariKrishna Kishore UmapathiAbdul Arif KhanKrishna Mohan SurapaneniAbdul RohmanKrishna YerraguntlaAbdul SirajKrishnamachari JananiAbdulbari BenerKrishnarjuna BankalaAbdulkadir ÖzgürKristen Donelle BrantleyAbdulkarim HasanKristian BratAbdulla Al-RashdanKristian HeldalAbdullah GibrielKristian LeisegangAbdullah ShaitoKristijan SkokAbeer Attia TawfikKristin Elizabeth SznajderAbel Po-Hao HuangKristina AndrijauskaiteAbelardo Claudio Fernández ChávezKristina ClarkAbhijeet JoshiKristina KopevaAbhijit AithalKristina LorenzAbhijit MondalKristine HeidemeyerAbhijit PakhareKristine KrajnakAbhijit Sukumaran NairKristine RuppertAbhijit V. LeleKristofer AndréassonAbhik SahaKristoffel R. DumonAbhilasha SinhaKristopher R. GenschmerAbhishek KumarKrisztian GasparAbilash NairKrunoslav M. SvericAbin SajanKrunoslav T. StinglAboElenain MansourKrystel Nyangoh TimohAbouzar NajafiKrzysztof DąbkowskiAbraham DibKrzysztof DowgierdAbraham López VivancosKrzysztof GomułkaAbraham PouliakisKrzysztof GorynskiAbraham Wall MedranoKrzysztof JurekAbu Bakar Hafeez BhattiKrzysztof KaczmarekAbubakr H. MossaKrzysztof KaliszewskiAchille MarinoKrzysztof LetachowiczAchilleas BoutsiadisKrzysztof LewandowskiAdalberto VieyraKrzysztof MuchaAdam B. EdwardsKrzysztof MyrdaAdam D. BramowethKrzysztof OzierańskiAdam FabisiakKrzysztof PiersialaAdam GoodieKrzysztof SzyfterAdam JanasKrzysztof TomasiewiczAdam KernKrzysztof WięckowskiAdam LewisKrzysztof ŻmudkaAdam LinderKseniia D. IevlevaAdam M. ZawadaKuang-Chun HuAdam QuickKuang-Tai KuoAdam ReichKuang-Ting YehÁdám SchléglKuan-Han LeeAdam StrzelczykKuan-Ting WuAdam WalkowiakKuanwei ShengAdam WylęgałaKuan-Wen WuAdel El-NaggarKuan-Yin LinAdelaida AvinoKuan-Ying HsiehAdele MitrottiKugimiya YoshihiroAdeniyi Michael AdebesinKui ZhangAdhiambo M. WitloxKułak WojciechAdi AranKuldeep ShahAdi LahatKuljit SinghAdib Al-Haj HusainKulkarni Uday PrakashAdil AsgharKumail Kumar MotianiAdina AbdullahKumiko MutaAdina StoianKun Bo ParkAdina ThomaidisKun Hee LeeAditi GuptaKun TianAditi SwarupKun WangAditya GoudKunal AjmeraAditya SharmaKunal PalAditya ShreenivasKundan MishraAdnan AynaKun-Hui ChenAdnan KhanKuniaki OtaAdolfo PerrottaKunihiko HiramatsuAdriaan Anthonie Van BodegravenKunihiro TsukasakiAdriaan MoelkerKuniya AsaiAdrian CurtoKunzheng WangAdrian Garcia-MorenoKuo-Ching YuanAdrián LozanoKuo-Chuan HungAdrian ManKuofeng HungAdrian OoKuo-Tai ChenAdrián Pérez-ArandaKuo-Tung TangAdrian SmedowskiKurahara Hai-LinAdrian Vasile MuresanKursad UnluhizarciAdrian W. MesserliKurt A. MelstromAdrian Zaragoza-BastidaKurt DeblAdriana AlbuKurt NaberAdriana Barbosa RibeiroKurt SvärdsuddAdriana GrigorașKurtis SwansonAdriana SeberKushal GandhiAdriana VinhasKushala W. M. AbeysekeraAdriana VisonàKustiariyah TarmanAdriana ZeeviKwang Sun RyuAdriane Ribeiro RosaKwangil YimAdrianna DouvrisKwon Hui SeoAdriano AraujoKye Hun KimAdriano MollicaKyeongmin KwakAdriano MurroneKyeong-Tae LeeAdriano ValerioKyle SueAdrie VerhoevenKyohei KinAdrien BreimanKyoichi ObataAdrien CarmonaKyong IhnAdrija HajraKyoung Sik ParkAdriyan PramonoKyoung-Chan ParkAfrasim MoinKyoung-Min ParkAfrouz AbbaspourKyriaki KyriakidouAfshan BeyKyu Hyang ChoAfshan MasoodKyu Sup LeeAftab TaiyabKyu Young ChoiAfua Adjeiwaa MensahKyu-Nam KimAfzal HussainKyung Eun HanAgam BansalKyung Eun LeeAgata GabryelskaKyung Jae YoonAgata JanowskaKyung Pyo KangAgata Joanna MajosKyung Rae KoAgata KlejmanKyunghee KangAgata M. SakowiczKyunghoon MinAgata MichalakKyung-Hun KimAgata OrzechowskaKyuseok KimAgata Poniewierska-BaranKyuwon ShimAgata Skrzat-KlapaczyńskaL. M. FiroozmandAgata SzulcL. Maximilian BujaAggeliki VerveniotiL’udovít GašparAgieshkumar Balakrishna PillaiLadan GolestanehAgnė Laučytė-CibulskienėLadislav BatalikAgnès Lillo-Le LouëtLadislav DokládalAgnès RibesLafon-Hughes LauraAgnieszka AdamekLaGuinn SherlockAgnieszka BossowskaLai Peng ThamAgnieszka GniadekLaís F. BerroAgnieszka J. SzczepekLajos GergelyAgnieszka Kalinska-BieniasLaleh JamshidiAgnieszka KasiukiewiczLalitha GummidiAgnieszka KolacinskaLambros MessinisAgnieszka KotalczykLamin B. ChamAgnieszka Kubicka-TrzskaLampros FotisAgnieszka MatuszewskaLampros K. MichalisAgnieszka Mazur-BialyLana NežićAgnieszka Owczarczyk-SaczonekLara BaticicAgnieszka Rynda-AppleLara C. KovellAgnieszka SławutaLara MorleyAgnieszka Sobierajska-RekLarisa AnghelAgnieszka StawarskaLarisa J. GeskinAgnieszka Świdnicka-SiergiejkoLarisa LitvinovaAgnieszka WąsikLarisa RyskalinAgnieszka ZyromskaLarissa Vivan CestonaroAgostino OgnibeneLarry R. JunckAgustin AlbillosLars ClemmensenAgustín Díaz-ÁlvarezLars HeggelundAgustín R. MirandaLars JødalAharon WegnerLars NorgrenAhmad AfifyLars WagnerAhmad AlbaghdadiLars-Peter KamolzAhmad AlsayedLars-Rene TueckingAhmad JabriLaszlo DeresAhmad JamlehLászló SzabóAhmad MirzaLászló VáróczyAhmad R. AbuzinadahLaszlo ZavoriAhmad SaltiLatif SumeraAhmed Abd El Aal SultanLaura AcquasalienteAhmed AbdelghanyLaura Adelaide Dalla VecchiaAhmed Abu-AwwadLaura Amaya-PascasioAhmed ArafaLaura BäzAhmed BahgatLaura BozzutoAhmed BakillahLaura BurattiAhmed DoghishLaura CannavòAhmed ElamragyLaura CipollettaAhmed ElmassryLaura Cortés-SanabriaAhmed EsmatLaura CorvattaAhmed GalhoumLaura De Armas-RilloAhmed HasanLaura De Graaff-HerderAhmed HieawyLaura DemartiniAhmed Hussieny ElbarbaryLaura Dos-SubiràAhmed IsmaeelLaura FoxAhmed KhaledLaura Fusar-PoliAhmed M. ZeitounLaura G. GiordaniAhmed MohsenLaura GiacomelliAhmed Nadeem AbbasiLaura GianottiAhmed S. AliLaura H. HaysAhmed Samir AbdelhafizLaura J. RushAhmed Ud DinLaura LindsayAhmed WahidLaura MarchiAhmed Yaseen AlqutaibiLaura MarcianoAhmet AltanLaura MaziluAhmet AyazLaura Mora-LópezAhmet BaydinLaura MorbidoniAhmet BaydurLaura MoschinoAhmet ÖzLaura N. HannivoortAhmet TanhanLaura Naemi WaltiAhsan Zil-E-AliLaura Parra-AnguitaAhsun RiazLaura PerimanAida PetcaLaura PetriniAïda PujolLaura PorrettiAikaterini D. KoutsouLaura SäisänenAikaterini I. LiakouLaura SalvadoriAikaterini MantakaLaura SanapoAikaterini PistikiLaura VicenteAiko TanakaLaura VitielloAimen KhacharemLaura WallardAimilia MantzouLaure Abensur VuillaumeAi-Ming WongLaure FlurinAinhoa Robles-MezcuaLaure Peyro-Saint-PaulAiping LiuLaureline BertelootAiqing LiLaureline BerthelotAisha FarhanaLauren J. LukAishwarya RavindranLauren K. WarehamAiste GintauteLauren LiebermanAistė PranckevičienėLaurence DedekenAisyah ElliyantiLaurence IserinAiyoub PezeshgiLaurence RozenAja AravamudhanLaurent DemizieuxAjai AgrawalLaurent GenserAjay GuptaLaurent GuyotAjay Kumar MishraLaurent MullerAjay Kumar VermaLaurent SebbagAjay Kumar VijayLaurent SuppanAjay PalLaurent WillemsAjinkya PawarLaurenz WeitgasserAjoe John KattoorLauri SoinneAjoy Prasad ShettyLaurie M. ElitAjoy VermaLavinia DuicăAkanksha SinghLavinia RechAkhil VarshneyLawrence B. FaulknerAkhtar AkhtarLawrence Chukwudi NwabudikeAki KatoLawrence P. McMahonAkihiko HiyamaLawrence Yu-Min LiuAkihiko KatayamaLaxman MainaliAkihiko MurayamaLayla A. GouldAkihiro KanamoriLayth Mula HussainAkihiro KanematsuLazar U. VelickiAkihiro OhsumiLazar VelickiAkihiro TamuraLazaros TzelvesAkihito TsubotaLazzaro ParaggioAkiko HanyudaLeandro PernambucoAkiko TanakaLeandro R. D. SanzAkiko YokoiLebon NicolasAkinari SawadaLech B. DobrzańskiAkinobu NakamuraLech ChrostekAkinobu SuzukiLee Ann Laurent-ApplegateAkio OishiLee B. BeermanAkira MinouraLee Samüel WannAkira MonjiLee TanAkira MorimotoLegese ChelkebaAkira NakaoLei GuoAkira NifujiLei WanAkira NishiyamaLei YanAkira ShimizuLeila AbdelhamidAkira SugawaraLeila AllahqoliAkira YoshidaLeila SimaniAkitatsu HayashiLein‐Ray MoAkito NakagawaLeire PedrosaAkiyoshi MizumotoLemoine CarolineAkkan AvciLena FrancescoAkos KusnyerikLena K. L. OestreichAkram AhangarpourLena Nilsson-WikmarAkram Hernández-VásquezLena TschidererAkshara RaghavendraLena Von KochAkshay GoelLennard SchröderAkshay KumarLeny JoseAkshay PandeyLeo L. TsaiAkshyaya PradhanLeo MassariAla NozariLeo RazakamanantsoaAlaa GhaliLeonardo Catalano-IniestaAlaattin OzenLeonardo CentonzeAlain AttarLeonardo César CarvalhoAlain BernardLeonardo D’AcquistoAlain Favre-RéguillonLeonardo FabbriAlain SarauxLeonardo FranzAlak MannaLeonardo ManzariAlan BonderLeonardo MastropasquaAlan D. FriedmanLeonardo PellicciariAlan Fleischer Jr.Leonidas PalaiodimosAlan FongLeonie VenemaAlan G. FinkelLeonzio FortunatoAlan GallinganiLeopoldo ArdilesAlan Kawarai LeforLeor PerlAlan KirkLeping LiAlan Lins FernandesLesley BreechAlan MorrisonLeslie Chavez-GalanAlan S. KaufmanLeslie Marisol Lugo GavidiaAlan SugarLeszek DrabikAlannah M. SmrkeLetizia CastelliAlarico ArianiLetizia Corinna MorlacchiAlauddin BhuiyanLetizia DeantonioAlba CorellLeuridan Cavalcante TorresAlban Fouasson-ChaillouxLia PulsatelliAlbert A. De GraafLiam O’NeilAlbert Arizá-SoléLiana DeheleanAlbert Clará-VelascoLiana PlesAlbert Estrugo-DevesaLiang HuAlbert H. ChaoLiang Ting LinAlbert Stuart ReeceLiang YanAlbert T. AnastasioLibin LiuAlbert Thomas AnastasioLi-Ching ChangAlbert WabneggerLidia Cabañes-MartínezAlbert Youngwoo JangLidia GavicAlberto Adanero VelascoLidia PerencAlberto AntonicelliLidija PoposkaAlberto BallestínLidija SavićAlberto BarbieriLies LahousseAlberto BollettaLieven De WildeAlberto CellaLigia RusuAlberto CeriniLiisa TomppoAlberto ChighineLijuan SunAlberto Di BlasioLikwang ChenAlberto Di SommaLilach GavishAlberto FerrareseLilach SoreqAlberto Francesco CeredaLilia González-CerónAlberto GrassiniLilia Maria Rizo-TopeteAlberto Gutierrez-CalvoLilian LacourpailleAlberto Jorge Oliveira LopesLiliana BachsAlberto Jorge-MoraLiliana Szyszka-SommerfeldAlberto Lo GulloLilith Caballero AguilarAlberto MaccariLilla MakszinAlberto MacchiLilly-Ann MohlkertAlberto MartinoLimor Muallem-KalmovichAlberto MellaLin ChengAlberto NogalesLin WuAlberto PalazzuoliLina Bezdetnaya-BolotineAlberto PariseLina HandayaniAlberto PianaLina YoussefAlberto PiccaLinchen HeAlberto PozzoliLinda DiehlAlberto RecchioniLinda GailīteAlberto RighiLinda J. Larson-PriorAlberto TommasiniLinda K. RauschAlberto VerrottiLinda NiciAlberto Villarejo-GalendeLindsay BrownAlberto ZanettoLindsay L. LoundaginAlbino EccherLindy M. RossowAlbrecht GuntherLing KuoAldo Bruno GiannìLing-Feng WangAldo PezzutoLingsha JuAldo RoccaLinto ThomasAldo RoccaroLionel LerouxAldostefano PorcariLior LipskyAleix SolanesLiquan GuoAlejandra FernándezLiron Danay SinvaniAlejandro Botero-CarvajalLirong WangAlejandro Olivares-HernándezLisa CunninghamAlejandro Silva PalaciosLisa D. WigginsAlejandro Suarez-De-La-RicaLisa HansonAlejandro TelloLisa L. HunterAlejandro Y. Meraz-MuñozLisa LiebermanAleksandar DiklicLisa M. JohnsonAleksandar DžakulaLisa ShawAleksandar RakićLisa VorkAleksander ChaibiLisbet BrandiAleksander KuśLiselotte HolAleksander ZwierzLi-Tu ZhangAleksandr N. IshmatovLiubov TkachevaAleksandr PoliakovLiudmila MatskovaAleksandra A. StefaniakLiudmila P. SmirnovaAleksandra AsaturovaLiverana LaurettiAleksandra BlachutLivia Camargo GarbinAleksandra Gilis-JanuszewskaLivia Ionela BobuAleksandra IlicLivia VeressAleksandra JarońLivio VitielloAleksandra JoticLiviu BilteanuAleksandra Karczmarska-WódzkaLiviu MacoveiAleksandra Milic LemicLiwei ChenAleksandra Nitecka-BuchtaLi-Wen LeeAleksandra Palatyńska-UlatowskaLixin ChengAleksandra Piechota-PolanczykLiza JohannessonAleksandra RogowskaLizz Van Der HeijdenAleksandra RyłLjiljiana JovancevicAleksandra StachońLluís AsmaratsAleksandra StupakLluís MasmiquelAleksandra SzylińskaLocati EmanuelaAleksandra Truszczyńska-BaszakLok ShresthaAleksandras VilionskisLokesh KumarAleksey Michailovich ChaulinLong ShenAlen DžuburLongbo ZhangAlena FurdovaLongfei LiuAlena Ribeiro Alves Peixoto MedradoLor RandallAleš JerinLore DamsAlessandra AldieriLoredana Sabina Cornelia ManolescuAlessandra BerzuiniLorel E. BurnsAlessandra BorchielliniLoren E. SmithAlessandra K. MatsunoLorena DimaAlessandra LuccheseLorena Vega-ZelayaAlessandra Lucia FlucaLorena ZentilinAlessandra MeneghelLorenza BresciaAlessandra PierangeliLorenzo CostaAlessandra RomandiniLorenzo DragoAlessandra Verzelloni SefLorenzo GibelloAlessandro A. ViansoneLorenzo LippiAlessandro AndreaniLorenzo MalatinoAlessandro AntonelliLorenzo MontiAlessandro ArenaLorenzo StacchiniAlessandro CapucciLori D. GarmanAlessandro CorsiLoris MarinAlessandro De SireLorraine A. T. BoakyeAlessandro Di MaggioLorraine Sheena KasavenAlessandro FavilliLoubna BahijeAlessandro Ferdinando RuffoloLouie Mar A. GangcuangcoAlessandro GambellaLouis ArnouldAlessandro GiolloLouis BlierAlessandro GiudiciLouis KreitmannAlessandro GonfiottiLouise BenningAlessandro GranitoLouise BonnetAlessandro InserraLouise ÖhlundAlessandro Mandurino-MirizziLouise PittAlessandro MedoroLouise-Laure MarianiAlessandro MeduriLoukianos S. RallidisAlessandro MengozziLourdes GimenoAlessandro MorlaccoLourdes M. VarelaAlessandro PedicelliLovro LamotAlessandro PosaLuan M. ChuAlessandro RamieriLuana Amorim BiondoAlessandro RizzoLubica RauovaAlessandro RossiLubomir SkladanyAlessandro ScanoLuc FavardAlessandro ScoppolaLuca AmbrosioAlessandro TonacciLuca ArcariAlessandro ViganòLuca BaldettiAlessandro VinciguerraLuca BrazziAlessandro VittoriLuca BruschiniAlessandro ZannottiLuca BurroniAlessandro ZorziLuca CerritelliAlessia CozzolinoLuca CevolaniAlessia GallucciLuca CimaAlessia RenziLuca D’OnofrioAlessia VignoliLuca De LucaAlessio ArriviLuca De SimoneAlessio BruniLuca Di GianfrancescoAlessio ContiLuca FlesiaAlessio Danilo ComisLuca FranchinAlessio Filippo PeritoreLuca GazziniAlessio FranchinaLuca GiacomelliAlessio G. MorgantiLuca GiannellaAlessio GasperettiLuca Giovanni LocatelloAlessio MylonasLuca IdolazziAlessio PaladiniLuca IelasiAlex Al KhouryLuca Lo PiccoloAlex AlfieriLuca MastorinoAlex Buoite StellaLuca MenilliÁlex FerrerLuca MezzettoAlex HanLuca OrtensiAlex HoferLuca Oscar Redaelli De ZinisAlex Ortega-LoayzaLuca Paolo WeltertAlex SoldererLuca PierelliAlexa BonacquistiLuca RegusciAlexa HollingerLuca Salvatore De SantoAlexa KlettnerLuca Salvatore MengaAlexander B. BerdalinLuca SchwarzenbacherAlexander BerezinLuca SegretiAlexander Dimitrov ShinkovLuca SimioneAlexander EckertLuca ValerioAlexander GardnerLuca VerrecchiaAlexander HaraganLuca ZanoliAlexander IonidesLucas HaaseAlexander J. NedopilLucas LauderAlexander Joseph JonesLucas LiaudetAlexander K. SchusterLucas MarinacciAlexander KokkinosLucas Pinheiro BarbosaAlexander M. BernhardtLucas WiegandAlexander Maximilian EickhoffLucia CastelliAlexander MilstreyLucia FratinoAlexander OberhuberLucía González-BuendíaAlexander OsmolovskiyLucia GrengaAlexander RauLucia Ilaria BirtoloAlexander RitterLucia KendrováAlexander ShifrinLucia MirabellaAlexander SobinoffLucia RomoAlexander StahlLucia ScarabelAlexander StepanovLucia ScisciolaAlexander TamalunasLucia StanciakovaAlexander W. LohmanLucia StančiakováAlexander YakobsonLucia TricaricoAlexander Yu MeigalLucian CâlmâcAlexandra BálintLucian Florin DorobanţuAlexandra BöhmLucian PuscasiuAlexandra BurluiLuciana BordinAlexandra Cristina MunteanuLuciana LabancaAlexandra F. FreemanLuciana MunhozAlexandra K. KarachaliouLuciana TeofiliAlexandra KalogerakiLuciane KopittkeAlexandra LappasLuciano De BiaseAlexandra MartuLuciano Rodríguez-DíazAlexandra MateiLuciano WannessonAlexandra PereiraLucie-Marie ScailteuxAlexandra PerricosLucija Virovic-JukicAlexandra Porras-RamirezLucile BroséusAlexandra RomanLucilla BarbanoAlexandra RoudenkoLúcio Fernandes FerreiraAlexandre BuffetLucrezia Ginevra LulliAlexandre DumuscLucrezia MoggioAlexandre FabreLucy D. MastrandreaAlexandre Pérez-GonzálezLucy NorrisAlexandre PersuLucy WebbAlexandre SemionovLucyna TomaszekAlexandre TerrierLudmila Danilowicz-SzymanowiczAlexandrina S. NikovaLudmila MáčováAlexandros G. BrotisLudovic JondrevilleAlexandros G. SykarasLudovic TrefondAlexandros PapachristodoulouLudovica NucciAlexandros PoutoglidisLu-Han LaiAlexandros PsarrisLuigi A. M. J. G. Van RielAlexandru AchimLuigi Alberto PiniAlexandru BejinariuLuigi BennardoAlexandru Bogdan CiubaraLuigi CalifanoAlexandru BurlacuLuigi CappannoliAlexandru ConstantinescuLuigi CarboneAlexandru CorlateanuLuigi CavannaAlexandru Eugen PetreLuigi CirrincioneAlexandru Gabriel BejinariuLuigi Della CorteAlexandru LupuLuigi Di FilippoAlexandru PopaLuigi Di TommasoAlexandru SchiopuLuigi Federico RinaldiAlexandru UliciLuigi Francesco SaccaroAlexei ChukhlovinLuigi GianturcoAlexei SlepzofLuigi GrecoAlexey ChubarovLuigi La ViaAlexey DashkevichLuigi LoscoAlexey GlukhovLuigi MeccarielloAlexey MeshkovLuigi NapolitanoAlexey SuminLuigi PerottiAlexey SurovLuigi RemoreAlexia RoulandLuigi RiganteAlexios BatrakoulisLuigi SchiraldiAlexis DziedziechLuigi TritapepeAlexis G. M. CarrascoLuigi TroisiAlexis GuédonLuigi UccioliAlexis HermidaLuigia BruglieraAlexis TalbotLuigia D. De FalcoAlexsandra Fernandes PereiraLuigi-Alberto PiniAlfonso Alvarado-LorenzoLuis A. GonanoAlfonso CeraseLuis Andreu CaravacaAlfonso IbañezLuis Carlos Venegas-SanabriaAlfonso Martínez-NovaLuis Cobos-PucAlfred SimonLuis Exequiel IbarraAlfredas SmailysLuis F. ReyesAlfredo CaturanoLuis FernandezAlfredo ChettaLuis Fernando Falcón-ArañaAlfredo Cruz-GregorioLuis Filipe Moutinho LeitãoAlfredo Di LeoLuis Gerardo Natera-CisnerosAlfredo G. CasanovaLuis Giraldo-CadavidAlfredo Garcia-FernandezLuis Gómez PérezAlfredo IandoloLuis Gustavo Modelli De AndradeAlfredo Larrosa HaroLuis M. TeranAlfredo Malo-MansoLuis Mariano EstebanAlfredo Perales MarinLuís Mendes PedroAlhadi M. JahanLuis Miguel Sánchez LoyoAli AkalinLuis Pablo Cruz HervertAli AkilLuís Pacheco-FigueiredoAli Al KaissiLuis Sánchez-GuillénAli AlbarratiLuis Sánchez-LabradorAli H. AldoukhiLuís Serviá-GoixartAli HeidarianpourLuis Suarez-ArronesAli KhademLuis Suso-MartíAli Mahdavi FardLuisa AgnelloAli MahtaLuisa FrizzieroAli MontazeriLuisa GiacconeAli RostamiLuisa María BotellaAli S. M. JawadLuisa MasciulloAli SaeedLuisa RicciardiAli ZarrabiLuise WagnerAlice AvanciniLuis-Guillermo Oliveros-LópezAlice BonomiLuiz CarneiroAlice DoreilleLuiz Fernando SelistreAlice GrossiLuiz Flávio Cordeiro FernandesAlice HadchouelLuiz Gonzaga Vaz CoelhoAlice JacquensLuiza Marek-JózefowiczAlice LaffiLuka MatakAlice LuddiLukas B. MoserAlice RossiLukas JenneweinAlice S. DayLukas KoflerAlice VerticchioLukas Martin Müller-WirtzAlice Verticchio VercellinLukas MeyerAlicia FischerLukas PrantlAlicia Martínez-VareaLukas RasulićAlicia RovoLukáš ŠkoloudíkAlicia Ruiz-PomedaLukas StreeseAlicja BortkiewiczLukasz BuldakAlicja Ewa RatajczakŁukasz CichockiAlim M. DymovŁukasz DembińskiAlin Mihai VasilescuŁukasz JanuszkiewiczAlina Daciana ElecŁukasz KruszynaAlina DanetLukasz KrzychAlina Delia PopaŁukasz ŁapajAlina KuryłowiczŁukasz M. MilanowskiAlina V. DumitrescuLukasz MalekAlina VedutaŁukasz MazurkiewiczAline Rangel-PozzoŁukasz NawackiAline Silva De MirandaŁukasz PaczesnyAlireza AbouhosseinŁukasz PałkaAlireza GheiniŁukasz PietrzykowskiAlireza KarimiŁukasz RypiczAlireza TahamtanŁukasz RzepińskiAlisa JohnsonŁukasz SzymañskiAlisa LikhitsupŁukasz WiktorAlise KaltenieceŁukasz ZapałaAlison CliffordLuke ChongAlison E. Fox-RobichaudLuke J. NeyAlissa M. BanyaszLuke NormanAlizadeh HussainLulin HuangAljoša MandićLulu WangAlkiviadis VatopoulosLungu AdrianAlkmini T. AnastasiadiLuo MingyaoAlla SplichalovaLura LlullAlla Vash-MargitaLut TamamAllegra BattistoniLutz FreitagAllen WongLutz KonradAllison HodgeLuyao ZhouAlmea MatanockLuyu XieAlmudena González RoviraLuz Kelly AnzolaAloisio AnnamariaLuz MoranAlok KumarLw GreenenAlok MishraLydi M. J. W. Van DrielAlok SudLydia ÁlvarezAlona Emodi-PerlmanLydia Min-Ying SuAlp AtikLydia VisserAlp UcokLyle BurdineAlpamys IssanovLynn M. RusyAl-Shaimaa F. AhmedLyudmila I. RachekAlvarado-Ibarra MarthaLyudmila S. KorostovtsevaÁlvaro Conrado Ucero HerreríaM. A. González De La RosaAlvaro DowlingM. A. JabbarÁlvaro Gabriel Sepúlveda MartínezM. CellinaAlvaro Gallego-MartinezM. Christian BrownAlvaro Hermida-AmeijeirasM. De NadalÁlvaro López-DíazM. F. AhamedAlvaro MaciasM. I. F. J. OerlemansÁlvaro Monteiro PerazzoM. Isabel LozaÁlvaro Plaza ReyesM. S. WongAlvaro Rojas-PeñaM. V. Padma SrivastavaÁlvaro Zubizarreta-MachoM. Van DetAlvin J. MunsamyM. Ya. YadgarovAlvin S. DasMª Carmen Castillejos-AnguianoAlvise SernicolaMaarten F. BijlsmaAlyaa FaridMaarten GlibertAlyssa HuffMaarten LimperAmadeus DobrescuMaayan KonigsteinAmal BouzianeMacarena Rodríguez-FraileAmal LinjawiMaciej ChęcińskiAman KumarMaciej CzepitaAman RajpalMaciej DębskiAmanda Cunha Regal De CastroMaciej GawęckiAmanda HellstromMaciej J. KotowskiAmanda Jean VinsonMaciej JedlińskiAmanda SaksidaMaciej JozwikAmandeep SinghMaciej KaczmarskiAmandine AnastácioMaciej KrasnodębskiAmandine BichonMaciej MichalikAmaniel KefleyesusMaciej PolakAmankeldi SalybekovMaciej SałagaAmany MohamedMaciej SińskiAmany SholkamyMaciej SkrzypczakAmar KrishnaMaciej SosnowskiAmar PujariMaciej StaporAmarinder Singh SinghMaciej TomczakAmato SantoroMaciej W. SochaAmber G. HealyMaciej ZalewskiAmblessed OnumaMaciej ŻukowskiAmbreen AzharMackenzi PergolottiAmbrina A. QureshiMadalina CarpAmbrish SinghMadalina-Gabriela IliescuAmbrogio P. LonderoMadankumar GhatgeAmbuga BadariMaddalena Alessandra WuAmedeo LonardoMadeeha MalikAmeesh M. IsathMadelyn GillentineAmelia DrakeMadelynn ChanAmelia J. HessheimerMadhan JeyaramanAmelia MunstermanMadhivanan KarthigeyanAmelia PietropaoloMadhulata ChauhanAmerigo GiudiceMadhumita ChatterjeeAmílcar Silva-Dos-SantosMadhup RastogiAmin Talebi Bazmin AbadiMadhur SachanAmina NasriMadoka NakajimaAmine OunajimMads Langager LarsenAmir Ali HamidiehMaegen BorzokAmir JadidiMael LeverAmir MellatiMagalie BoguenetAmir Moghadam AhmadiMagda Ecaterina AntohéAmir R. KachooeiMagda ZanelliAmir Reza HajrasoulihaMagdalena A. BryndzaAmir S. YazdiMagdalena DudekAmirah GozaliMagdalena DurlikAmirali SalmasiMagdalena GębskaAmirhossein MirhashemiMagdalena GrzybowskaAmit FrenkelMagdalena HagovskaAmit GoelMagdalena HurkaczAmit Kumar MittalMagdalena Jastrzębska-WięsekAmit KunduMagdalena KołodziejAmit NahumMagdalena KostkiewiczAmit PantMagdalena KrajewskaAmit PorwalMagdalena KrbotAmit ReddyMagdalena LeszkoAmit SinghMagdalena LitwinskaAmita NandalMagdalena M. MaslonAmiya PatraMagdalena Mackiewicz-MilewskaAmjad RehmanMagdalena MarczyńskaAmmar EbrahimiMagdalena PaplinskaAmornluck KrasaelapMagdalena SzopaAmosy M’KomaMagdalena WalickaAmpaiwan ChuansumritMagdalena ZadwornaAmr Ahmed El‑ArabeyMagdalena ZgliczynskaAmra AdroviçMagdalena ŻychowskaAmritlal MandalMagdolna NagyAmro M. SolimanMagdy Ibrahim AlgowharyAmutha RamadasMaged M. A. FoudaAmy HinkelmanMagnus Bjorkavoll-BergsethAmy L. ShafrirMagnus SjogrenAmy LiMaha Emad IbrahimAmy LossieMaha Saber-AyadAmy Scott-ThomasMahadev RaoAmy SheppardMahdi HaghighatafsharAna Amorim-de-SousaMahdi MahdipourAna AulinasMahdi ZavvarAna Aurora Díaz-GavelaMahendran SekarAna Ballesta-CastillejosMaheswari MuruganandamAna BatallaMahipal SachdevAna Belén Santos-OlmoMahmood ChoudheryAna Beltrán VelascoMahmoud A. SenousyAna BlascoMahmoud AbdallatAna Carina FerreiraMahmoud BalbaaAna Carla Raphaelli Nahás-ScocateMahmoud BukarAna Carolina De CamposMahmoud DiabAna Carolina Silveira RabeloMahmoud ElbattahAna Catarina ÂngeloMahmoud HammadAna CiobanuMahmoudreza HadjighassemAna CoelhoMahmud HasanAna Corte-RealMahnaz Bahri KhomamiAna Cristina CamarozanoMahnaz EskandariAna DascaluMahsa DabaghAna Elena Pérez-CobasMai MostafaAna Flávia Fernandes FerreiraMai Salah El Din Mohamed HelmyAna García-MuñozMaicon Rodrigues AlbuquerqueAna Gascón-CatalánMaija CastrenAna I. ÁvilaMaija Huttunen-LenzAna IvaniševićMaike TrommerAna Leticia Fornari CapraraMaire PetersAna Luísa JoãoMairi PucciAna M. Cebrián-CuencaMaite E. Rivera GorrínAna M. Sánchez‑TorresMaja MiletićAna Maria BalahuraMaja MiskulinAna Maria CiubarǎMaja MizdrakAna María Fernández-AlonsoMaja PakižAna Maria GheorghiuMaja Rogić VidakovićAna Maria IordacheMaja Šikić PogačarAna Moreno-ÁlvarezMaja SurbatovicAna P. SantosMaja TomičićAna Paula BassiMajed AlamriAna Paula Duarte De SouzaMajid JadidiAna Paula MacedoMajid MoshirfarAna Paula TagliariMajid Mufaqam Syed-AbdulAna ReulaMakiko HiranoAna RochaMakkai-Popa Silviu-TiberiuAna Rosa Rincón-SánchezMakoto EndoAna Sánchez-AzofraMakoto FurugenAna SebioMakoto HaradaAnabik PalMakoto HosoyaAnamaria BechirMakoto InoueAnamaria CristinaMakoto IsozakiAna-Maria FloreaMakoto KondoAnan ShtayaMakoto NakamuraAnan SrikiatkhachornMakoto SugayaAnand MaryaMakoto SugiharaAnand ParamasivamMakoto SuzukiAnand Prakash SinghMakoto TakeiAnandharajan RathinasabapathyMalak El SabehAnant JainMalcolm Scott DuthieAnanth S. EleswarapuMalek Ben Ahmed AdouniAnanto AlhasyimiMalene PedersenAnastasia G. MitseaMałgorzata BlatkiewiczAnastasia KotanidouMałgorzata BrauncajsAnastasia MitseaMałgorzata Buksińska-LisikAnastasia SaadeMałgorzata ChlabiczAnastasia SveshnikovaMałgorzata CzogałaAnastasios ApostolosMalgorzata DebowskaAnastasios KartasMałgorzata DobrzyckaAnastasios KolliasMałgorzata Dorota SkibinskaAnastasios KoulaouzidisMałgorzata GajdzisAnastasios StathisMalgorzata KolodziejAnatol PrinzingMalgorzata KolpaAnca BobircaMałgorzata Kotula-BalakAnca Chelariu-RaicuMałgorzata Kulesa-MrowieckaAnca MareMalgorzata Maria JerzakAnca PanaitescuMałgorzata Mikaszewska-SokolewiczAnca SimionescuMałgorzata MizgierAnca ZguraMałgorzata MoszyńskaAnca-Olguța OrzanMalgorzata PihutAndaleb KholmukhamedovMałgorzata PorębaAnda-Petronela RadanMałgorzata ŚliwinskaAnder MatheuMalgorzata Sokolowska-WojdyloAnders EttrupMałgorzata StachońAnders GottsaterMalgorzata WasniewskaAnders Husted MadsenMałgorzata WisłowskaAnders KjellbergMałgorzata Zalewska-AdamiecAnders LarssonMalik AydinAnders MellemgaardMalik SallamAndi RrokuMalin BrundinAndoni Carrasco-UribarrenMalini MukherjeeAndra PiciuMalini SurapaneniAndrada SeiceanMalte HolschenAndrás BikovMalvinder S. ParmarAndrás MolnárMamata SivagnanamAndrás RabMamoru ShibataAndraž CerarMamoru TakahashiAndraẑ DovnikMamta Chawla SarkarAndre IgneeManabu NittaAndré L. L. BachiManal AlemAndre Luiz CostaManan ShahAndré MermoudManar KhashramAndre MessiasMandana KamgarAndré NohlMandy BusseAndré P. WolffManel Sabaté TenasAndre Paes Batista Da SilvaManel SantaféAndré RamonManfred P. LutzAndré Ricardo Araujo Da SilvaManfred PhilippAndre RodackiMang L. ChenAndre Van ZundertMangesh Prashant JoshiAndre WirriesMangla SoodAndrea AbateManickam AshokkumarAndréa Alice Da SilvaManik N. AggarwalAndrea BalduitManish DhawanAndrea BarberisManisha WitmansAndrea BarbieriManju DhandapaniAndrea BerniManju M. ChandraAndrea BiglioManju R. PillaiAndrea BiondiManlio SantilliAndrea BoccatondaManmohan SinghAndrea BruniManmohan SinghalAndrea BuscaroliManoj GargAndrea ButeraManoj H. IyerAndrea C. PostierManoj Kumar TembhreAndrea CioffiManon GijtenbeekAndrea CortegianiManon LaunayAndrea D’AmatoManouk De HoogeAndrea DekanicMåns MagnussonAndrea DeussenMansour A. MahmoudAndrea Di CataldoMansur DuranAndrea Duarte DoetzerManu PrasadAndrea ErmolaoManuel Aguilar-DiosdadoAndrea FabbriManuel Barreiro-PérezAndrea FabianiManuel Caro-MagdalenoAndrea FariniManuel Coheña-JimenezAndrea FontanaManuel Glauco CarboneAndrea FrosoliniManuel Gonzalez De La RosaAndrea FrustaciManuel GranellAndrea GalosiManuel Hermida PrietoAndrea GažováManuel José LopesAndrea GianniniManuel MadrazoAndrea Gonzalez-MontoroManuel Martinez-SellesAndrea GovettoManuel Mendez-BailonAndrea GrilloManuel MorrensAndrea Hawkins-DaarudManuel Ortega-CalvoAndrea JaníkováManuel Pardo-RiosAndrea KleindienstManuel Perez-MirandaAndrea LatiniManuel PestanaAndrea LauferManuel RamírezAndrea LazzeriniManuel Ribeiro Da SilvaAndrea Leti AcciaroManuel Rodríguez-PerálvarezAndrea MafficiniManuel Rodríguez-VallejoAndrea ManciniManuel Sanchez-DiazAndrea MarcinnòManuel Sánchez-MollaAndrea MichelerioManuel Sanchez-SolisAndrea Milostić SrbManuel StruckAndrea MilziManuel Teixeira VeríssimoAndrea MontaltoManuel TousidonisAndrea MontisciManuel Trinidad-FernándezAndrea MulliriManuel ValdebranAndrea MunafoManuel VilelaAndrea PantaloneManuel WilbringAndrea Paola Rojas GilManuela A. HoffmannAndrea PernaManuela DudeckAndrea PozziManuela RoncellaAndrea RegnerManwaring Jamie L.Andrea S. MelaniMao Qiang WangAndrea SambriMaolin LuAndrea SeilerMar Llamas-VelascoAndrea SpiniMara CarsoteAndrea SzékelyMara HorwitzAndrea TinelliMara Pavel-DinuAndrea TinnirelloMarat EzhovAndrea VecchiolaMarawan Abdel BasetAndrea WeinzierlMarc Antoine BenderraAndrea XodoMarc B. MuijzerAndreadi AikateriniMarc BernaAndreas BoeningMarc FischlerAndreas EbertMarc Giménez-MilàAndreas Eugen EnzMarc HanschenAndreas F. M. BorkensteinMarc HendrikxAndreas GoulesMarc HilhorstAndreas JokusziesMarc L. De BuyzereAndreas KalogeropoulosMarc LeoneAndreas KarakatsanisMarc LiesaAndreas KriegMarc OlivaAndreas LambertzMarc RussoAndreas MitsisMarc SimardAndreas PolydorouMarc Töteberg-HarmsAndreas RiesMarc VanderheydenAndreas S. KalogeropoulosMarc Xipell FontAndreas S. PapazoglouMarcel AdlerAndreas S. PetersMarcel F. KunrathAndreas SchlatterMarcel NaikAndreas SchwingshacklMarcel Olde RikkertAndreas Sebastian PetersMarcel Van’t VeerAndreas StahlMarcel Y. AvilaAndreas StallmachMarcela Martín-Del-CampoAndreas TrotterMarcela RamosAndreas TulzerMarcele Regine De CarvalhoAndreas UmgelterMarcella ZollinoAndreas ZielkeMarcello AugelloAndreas ZietzerMarcello CandelliAndree HartantoMarcello DeracoAndree KurniawanMarcello Magri AmaralAndreea AndronesiMarcello NaccaratoAndreea AvasiloaieiMarcello RomanoAndreea BorleaMarcelo Dutra ArboAndreea MotocMarcelo Fernandes Da CostaAndreea-Iren SerbanMarcelo N. MuscaraAndrei BraesterMarcelo PalinkasAndrei C. HolmanMarcelo Volpon SantosAndrei ChurylaMarcel-Philipp HenrichsAndrei ColitaMarchel S. VetrileAndrei FodorMarcin BączykAndrei Gheorghe MotocMarcin BalcerzykAndrei HavasiMarcin BasiakAndrei HolmanMarcin CzopAndrei Marian FeierMarcin DerwichAndreia Martins RosaMarcin DziekiewiczAndreia S. P. SousaMarcin FeldoAndrej BelančićMarcin FijałkowskiAndrej CokanMarcin GierekAndrej MarkotaMarcin GolczakAndrej ThurzoMarcin JozwikAndreja FigurekMarcin KozakiewiczAndreja Trojner BregarMarcin MalinowskiAndres Carrazco-MontalvoMarcin NicośAndrés Da Silva CandalMarcin RozalskiAndres Moreira-SotoMarcin SadowskiAndres YarurMarcin TyrakowskiAndreu Comas-GarciaMarcin WasowiczAndrew BentleyMarcin WełnickiAndrew C. BirkelandMarcin ZielińśkiAndrew Chih Wei HuangMárcio F. SimãoAndrew CovelerMárcio Lima GrossiAndrew D. MihalekMárcio Manozzo BoniattiAndrew D. RedfernMarco AllinoviAndrew E. BlumMarco AngelillisAndrew E. LittmannMarco AnileAndrew G. MillerMarco BattistaAndrew J. SmithMarco BongiovanniAndrew K. ChanMarco BruschiAndrew Li YimMarco CambiaghiAndrew Ming-Liang OngMarco CapecchiAndrew S. DayMarco CerranoAndrey CherstvyMarco ChiappettaAndrey D. NasledovMarco ChiostriAndrey FinegershMarco CiavarroAndrey GlotovMarco CicconeAndrey KuzminMarco De SioAndrey PetrovMarco Di NicolaAndrey SivkovMarco Di PaolaAndrey SudarikovMarco FerrariAndrey V. DubovoyMarco FerriseAndrija MatetićMarco FiorentinoAndrius PranskunasMarco FoganteAndroulla EleftheriouMarco GallusAndrzej BłażejewskiMarco GianiAndrzej BrudnickiMarco HaertleAndrzej CzamaraMarco KaufmannAndrzej HellmannMarco L. LeungAndrzej KoniecznyMarco La VerdeAndrzej LangeMarco MammanaAndrzej Marek ŚliwczyńskiMarco ManfrediniAndrzej MichalskiMarco Maria PascaleAndrzej NowickiMarco Matteo CicconeAndrzej P. OkoMarco MeloniAndrzej PrzybylskiMarco MeyerAndrzej SkrętMarco PalumboAndrzej TomczakMarco PappalardoAndrzej Za̧bekMarco PassiatoreAndy SiddawayMarco PicichèAndy T. Y. LauMarco RameraAndy Wai Kan YeungMarco RanucciAndy Wei-Ge ChenMarco SaettoniAne Murueta-GoyenaMarco Sanchez-GuerraAneeza W. HamizanMarco SapienzaAnel Gómez GarcíaMarco SavareseAnestis KarakatsanisMarco SchiavoneAneta AleksovaMarco SerafinAneta Cymbaluk-PloskaMarco TatulloAneta GrochowskaMarco TofaniAneta Ostróżka-CieślikMarco TomasettiAnetta MüllerMarco TonelloAnette Ramm-PettersenMarco TorresAnge WangMarco TubaroÁngel Augusto Pérez-CalatayudMarco Valerio MarianiAngel BatuecasMarco ValvanoÁngel BecerraMarco Vito MarinoAngel Cuadrado GarcíaMarco ZuinÁngel Estella-GarcíaMarco. A. ReynaAngel L. Fernández-GonzálezMarc-Olivier GauciÁngel L. GuerreroMarcos BrioschiÁngel Ramos-De-MiguelMarcos Edgar HerkenhoffÁngel SevillanoMarcos Fernando Xisto Braga CavalcantiAngela AmmirabileMarcos Navarro-SantanaAngela BentonMarcos PeriniAngela Georgia CaticMarcus BahraAngela Graciela Deliga SchroderMarcus D. LanceAngela NarayanMarcus O. HarringtonAngela RoseMaree A. MilrossAngela S. KohMarek BiesiadeckiAngela ShierkMarek BolanowskiÂngela Toshie Araki YamamotoMarek BugajskiAngela Yen MooreMarek KozińskiAngel-Augusto Perez-CalatayudMarek LančAngeli Christy YuMarek NahajowskiAngelica PetraroliMarek NalosAngelika V. TimofeevaMarek PaulAngeliki AngelidiMarek PieszkaAngelina MeirelesMarek RoikAngeline RouersMarek SierżęgaAngelique EgbertsMarek SlávikAngelita Maria StabileMarek SzwiecAngelo BellinviaMarek UssowiczAngelo CarneiroMarek W. KarwackiAngelo Di GiorgioMargaret StefaterAngelo G. TorrenteMargaret WallenAngelo M. CarellaMargaret WallhagenAngelo NaselliMargarida Freitas-SilvaAngelo RuggieroMargarit SievertAngelo ScuteriMargarita StankovaAngelo SilverioMargherita BarbutiAngels EscorsellMargherita CerroneAnida Maria BabtanMargherita PizzicannellaAniello FedericoMargie A. ReamAniello MaieseMargit SerbanAnil ChekuriMargot ErnstAnil Kumar JonnalagaddaMargrit LoebnerAnindya GangulyMaria A. BaturovaAnindya MukherjeeMaría A. Pérez-HerreroAnirejuoritse BaforMaria A. RadanovaAnirudh SharmaMaria AbbattistaAnish BahraMaria Adele GiamberardinoAnish MukherjeeMarià AlemanyAnish ShivaramMaria Alexandra PapadimitriouAnita BakraniaMaría Alonso De LeciñanaAnita L. NelsonMaria AmbrosioAnita Lyssek-BorońMaría Amparo Blanch-RuizAnita VetraMaria AndreassiAnja HarderMaria Angela CerrutoAnja Kathrin JaehneMaria Angela Zaccarelli-MarinoAnja M. BoosMaria Angelica GuimarãesAnja MottokMaria Angelica MiglinoAnja WeiseMaria Anna DonatiAnjali A. SatoskarMaría Antonia Parra-RizoAnjali GeethadeviMaría Antonia Saornil-ÁlvarezAnjali LepchaMaria AntoniadouAnji E. WallMaria António CastroAnke LangenfeldMaria ArioliAnkit SrivastavaMaría Arregui GambúsAnkush KawaliMaria Augusta Bento Cicaroni GibelliAnn A. VerhaegenMaria Bacci GiacomoAnn Marie EgloffMarıa Benito-De-PedroAnna Bednarska-CzerwińskaMaria Borrell-PagesAnna BersanoMaria BovaAnna BiernasiukMaria C. AlonsoAnna BogaczMaria CamposAnna Bogusława Pilewska-KozakMaria Carmen AcostaAnna BrzeckaMaria Carolina MendesAnna CampanatiMaria Carolina PaulinoAnna CamporesiMaría Carrillo-DíazAnna CastelnovoMaria Chiara DittoAnna Christine NilssonMaria Chiara GattoAnna ComparelliMaria Chiara MeucciAnna CybulskaMaria Concetta PastoreAnna CzarneckaMaria CorreiaAnna DanielewiczMaria Costanza ChitiAnna Duda-MadejMaria Cristina BudaniAnna E. PłatekMaria Cristina MondardiniAnna Erkiert-PolgujMaría D. ArguisuelasAnna ErmundMaria Das Graças CarvalhoAnna Felis-GiemzaMaría Del Mar Fernández-MartínezAnna FreyMaría Del Mar Jiménez LasserrotteAnna FurlanMaria Del Mar Pacheco HerreroAnna Grzywa-CelińskaMaria Del Pilar Estevez-DizAnna HeidbrederMaria DemetriouAnna HymosMaria DenchevaAnna Jamroz-Wis̈niewskaMaria DigiacomoAnna Kablak-ZiembickaMaria Dolores Garcia De LucasAnna Kasielska-TrojanMaría Dolores López-CarmonaAnna KawalecMaria Dos Anjos DixeAnna KozłowskaMaria DudarevaAnna KucharskaMaria Elena CapraAnna La SalviaMaria Elena RiccioniAnna LevinssonMaria Elisa MancusoAnna LisowskaMaría F. Tano De La HozAnna Luiza SzeszMaría Fe Bravo-OrtizAnna LyakhovitskyMaria Fernanda Reis Gavazzoni-DiasAnna M. LebedevaMaria FortofoiuAnna M. Różańska-WalędziakMaria Francisca Colella-SantosAnna MajewskaMaria FrantziAnna MaltagliatiMaria G. TektonidouAnna Maria FrustaciMaria Gabriela O. FernandesAnna Maria HeikkinenMaria Giulia BolognesiAnna Maria MalagoniMaria Giulia DemarzoAnna Maria RoszkowskaMaría Granados SantiagoAnna Maria TartaglioneMaria Grazia AndreassiAnna Maria ZajenkowskaMaria Grazia CappaiAnna MatyniaMaria Grazia CelaniAnna MertasMaria Grazia ScarpaAnna Michalak-StomaMaría Guadalupe Frías-De-LeónAnna MichnoMaria HukkinenAnna MosiołekMaria IskraAnna Mucha-MałeckaMaría J. López-ArmadaAnna Nowak-WegrzynMaría Jesús HerreroAnna NowinskaMaría José Bautista-LlamasAnna Olasińska-WiśniewskaMaría José García-PolaAnna Olewicz-GawlikMaría José Pujalte-JesúsAnna OliverasMaria JustineAnna PadoaMaria LemboAnna Paradowska-StolarzMaría Llorián-SalvadorAnna PellatMaria Lorenza MuiesanAnna PiekarskaMaria Luce CaputoAnna PiotrowskaMaria Luisa MarvánAnna PocurullMaria Luisa ZeddeAnna PodlasekMaria MaccaroneAnna PolewczykMaria Malvina TsamouriAnna PresicciMaría Maricela Carrasco-YépezAnna R. YousafMaria MartzoukouAnna Rita FetoniMaria MatuzAnna RogalskaMaria MazzitelliAnna RoszkowskaMaria Mei Ha WongAnna RozaliyaniMaría MeloAnna RozensztrauchMaria Michela ChiarelloAnna RuggieriMaria Oana SasaranAnna S. TzortziMaria OrdonezAnna ShcheglovaMaria P. A. BaldassarreAnna ShmakovaMaria P. NtaloukaAnna ShtroMaria P. YavropoulouAnna SiemiątkowskaMaria Paola BertuccioAnna SobiepanekMaria PaparoupaAnna Sonia PetronioMaria PassanteAnna StarzyńskaMaria Pia FerrazAnna StefanskaMaria PiancinoAnna SzumilewiczMaria PichlerAnna Tankiewicz-KwedloMaría Pilar BallesterAnna TurkinaMaría Pilar López-RoyoAnna Valeria SamarelliMaria Polina KonstantinouAnna ViolaMaria PuiuAnna Waśkiel-BurnatMaria RasoAnna WozniackaMaría Reina BuenoAnna WozniakMaria Respondek-LiberskaAnna WrzosekMaria Rita BiancoAnna Zalewska-JanowskaMaria Rosaria VarìAnna ZwierzchowskaMaria SamaraAnnabel BlasiMaria SarapultsevaAnnabelle DupontMaria Sebastiana SilvaAnnalisa BoscoloMaría SerramitoAnnalisa CappellaMaria SimonenkoAnnalisa Di CelloMaria SobridoAnnalisa PaceMaria Stella EpifanioAnna-Maria BarciszewskaMaria Swennergren HansenAnnamária KosztinMaria SzubertAnnamaria PetitoMaría Teresa García-SanzAnnamaria PorrecaMaría Teresa García-UnzuetaAnna-Maria SchneiderMaria Teresa GuagnanoAnnarita BaroneMaría Teresa MartínezAnne BlaisMaria Teresa VillaniAnne Braae OlesenMaria TotanAnne C. SullivanMaria V. TurovskayaAnne CornelissenMaria VelascoAnne FalcouMaria Vittoria CicinelliAnne FlöckMaria Vittoria CubellisAnne G. RaafsMaria Wieteska-MiłekAnne K. GalgonMaria WitteAnne KargeMaría Yolanda Castellote-CaballeroAnne M. DorranceMaria Zelinda RomanoAnne Prigent-TessierMaria ZverevaAnne RegazzettiMaría-Carmen Sánchez GonzálezAnne Sophie BoureauMariaconcetta LongoAnne TailleuxMariaconsiglia FerrieroAnne-Cécile PaepegaeyMaria-Costanza ChitiAnnekathryn GoodmanMariaenrica TinèAnnekatrin CoordesMaria-Fernanda Lorenzo-GómezAnne-Laure SimonMariagrazia PiccioniAnnelien DuitsMariagrazia RossiAnnemiek NapMarialaura SimonettoAnnemieke Oude Lansink-HartgringMariam Margvelashvili-MalamentAnne-Priscille TrouvinMarian SimkaAnneza YiallourouMarian Y. GirgisAnnibale PucaMariana BenderskyAnnie HasibMariana FloriaAnnika StrandellMariana Fragão-MarquesAnnina B. SchmidMariana NucciAnn-Kathrin KahleMariana P. SocalAnnunziata NuscaMariana Pereira AntoniassiAnoop RawatMariana TudoranAnoopum S. GuptaMariangela ManciniAnselm A. DerdaMariangela ScavoneAnselm JordaMariángeles DíazAnselm Sebastian UebingMarianna E. WeenerAnselmo CaricatoMarianna KulkaAnsgar SchulzMarianna RanieriAnshul BhallaMarianna TriantouAnshul RaiMarianne BrodmannAnshumali MittalMarianne HollensteinerAn-Suey ShiaoMarianne ZellerAntanas VaitkusMariano E. MenendezAnthony CheungMariano FerraressoAnthony GloverMariarita TaralloAnthony GuihurMariarosaria CampiseAnthony KondrackiMarie BobowskiAnthony L. KovacMarie CarbonnelAnthony Michael HunterMarie Dam LauridsenAnthony PappMarie De BoutrayAnthony R. AbsalomMarie M. BudevAntigoni SakellaropoulouMarie MarschalekAntje BanningMarie Maude GeoffrayAntje Büttner-TeleagăMarie ThomasAntoine ChatrenetMarie-Cécile NassogneAntoine GennariMarie-Helene MarionAntoine NaemMarie-Hélène Metz-BoutigueAntoine SalibaMariela CorralesAnton KiselevMarie‐Laure JourdainAnton MoritzMariella D’AngiòAntonella CiabattoniMarie-Odile Soyer-GobillardAntonella Di CesareMariia ZhukovaAntonella LeonettiMarija M. BožićAntonella ScorzielloMarija ŽarkovAntonella StravatoMarijana CurcicAntonella TammaroMarijana VičićAntonia KondouMarijke DieltjensAntonieta Guerrero-PlataMarijn SpeeckaertAntonietta CoppolaMarika C. CoffmanAntonietta GiganteMarika LanzaAntonietta LiottiMarika QuadriAntonija TadinMariko KawashimaAntonin BrisudaMarilda De Souza GonçalvesAntonin KrajinaMarilena GregoriniAntonin TrimailleMarilène FilbetAntonino Lo GiudiceMarilia CarabottiAntonino ManiaciMarilia TrevelierAntonino MignanoMarina CavuotoAntonino TuttolomondoMarina Consuelo VitaleAntonio AmmendoliaMarina E. CazzanigaAntonio Andrea GrossoMarina Elena CazzanigaAntonio BellasiMarina LeitmanAntonio BellastellaMarina MartelloAntonio BorzelliMarina Mat BakiAntonio BrunoMarina MencingerAntónio CamposMarina MichalakiAntonio CantalapiedraMarina PericAntonio Carlos XimenesMarina Rodríguez-Calvo-De-MoraAntonio CeliaMarina RomozziAntonio CicioneMarina Ruxandra OteleaAntonio Córdoba FernándezMarina S. DukhinovaAntonio CovielloMarina Sastre-IbañezAntonio D’AmatiMarina SoroAntonio De TantiMarina Šprem GoldštajnAntonio De VitaMarina ValenteAntonio Del CasaleMarina Zaric KonticAntonio Di ChiaraMarina ZeldovichAntonio FacciorussoMarine ForienAntonio FrancoMarinela BostanAntonio GidaroMario Basulto-MartinezAntonio González-BulnesMario Bernardo-FilhoAntonio GrecoMario BilićAntonio J. OrtizMario Chico-FernándezAntonio KokotMario Damiano ToroAntonio LacquanitiMario DauriAntonio LaluezaMario Di GioacchinoAntonio M. Pico AlfonsoMario DiazAntónio MachadoMario Enrico CanonicoAntonio ManentiMario Felice TecceAntonio Maria FeaMario FruschelliAntonio Martinez-LopezMario G. BianchettiAntonio MarzolloMario GanauAntonio MassaroMario HirataAntónio Mesquita MontesMario J. Valladares-GarridoAntonio MolinoMario LimaAntonio NarzisiMario LovrićAntonio NennaMario Luca MorieriAntonio OrlacchioMario MarzilliAntônio Pazin-FilhoMario MascalchiAntonio Pérez-RuedaMario Mendoza-SagaonAntonio PiresMario MiniatiAntonio Pompilio GiganteMario PacilliAntonio PontorieroMario Pelaez-LunaAntonio Popolo RubbioMario SenechalAntonio PortolesMario ŠokacAntónio QueirósMario Ulises Herrera-PérezAntonio Ramos MartinezMario VendittiAntonio RizzaMărioara ȘimonAntonio RomanelliMarion J. M. TorrèsAntonio RomanoMarion RabantAntonio SalsanoMarion SalléeAntonio SantagostiniMarion SchaeferAntonio Sarría SantameraMarion TomičićAntonio SerranoMariona RiusAntonio SommarivaMarios G. KrokidisAntonio SteccoMarios PapadakisAntonio TufanoMarios PapasotiriouAntónio Valentim GonçalvesMarios RossidesAntonio Videira Da SilvaMarisa AvvedimentoAntonio ZanghiMarisa FilipeAntonio-Javier ChamorroMarisa MarquesAntonios CharokoposMarisa ShiinaAntti PerheentupaMarish OerlemansAntuani R. BaptistellaMarisol OcampoAnu Anna GeorgeMarit KalisvaartAnudishi TyagiMarita GilljamAnuradha ChakrabortiMaritza N. Puray-ChavezAnuradha GodishalaMarius A. ØvrehusAnwar A. SayedMarius Gustav BredellAo ZhangMarius J. CoetzeeAparna ShindeMarius NedelcuApichat KaewdechMarius RademakerApolline ImbardMariusz DabrowskiApolonija Bedina ZavecMariusz DrużbickiApostol Ciobanu Delia GabrielaMariusz DubielApostolos BeloukasMariusz G. FleszarApostolos I. TsolakisMariusz KlenckiApostolos MamopoulosMariusz KlopotowskiApostolos PerelasMariusz NiemczykAraceli Ortiz RubioMariusz SikoraAram BoadaMariusz TomaniakAram YangMariya A. SmetaninaArash MotekallemiMarjan SalariArastoo NiaMarjan Wouthuyzen-BakkerArcady MarkelMarjolaine GossetArchi Ramesh AgrawalMarjolein KluytmansArda GülerMark A. LikerArely Vergara-CastañedaMárk Ádám AntalAremis VillalobosMark BuchfuhrerAreti OkalidouMark ClemensArgiris S. SymeonidisMark Dominik AlscherAri LeshnoMark E. MurphyAria NouriMark Elisabeth Theodorus WillemsAriadni SpyroglouMark EvansArian ZaboliMárk F. JuhászAriana AlbaneseMark F. SommerfeldtAriane BerdalMárk FráterArieh EdenMark FreedmanAriel Soares TelesMark G. BowdenArielle L. OlickerMark G. LebwohlArietta SpinouMark GorrellArik WolakMark H. BilskyArio De MarcoMark Hans EmanuelAristi BoulmpouMark J. LambrechtsAristotelis PerrakisMark J. MidwinterAriz AkhterMark J. RoefArjan VissinkMark K. FarrugiaArjun K. NambiarMark K. TimmonsArkadiusz KazimierczakMark M. WalshArkadiusz KrzyżanowskiMark McCauleyArkadiusz SzarekMark McGurkArkady KotlyarMark MillerArkasubhra GhoshMark PlazierArkom ChaiwongkotMark S. MandelcornArleta DrozdMark S. TaylorArman ArghamiMark SuterArmando CaseiroMark ThomasArmando L. OliverMark W. GreenArmando SenaMarkku J. NissinenArmin Niklas FlinspachMarko BaškovićArmond DaciMarko KumrićArnaldo AmatoMarko KutlešaArnaldo DubinMarko TarleArnaldo ScardapaneMarko VelepicArnaud AlvesMarko VuletićArnaud DesclauxMarko ZdravkovićArne BöttcherMarko ZelićArne BurssensMarkos KlonizakisArne ViestenzMarkus B. SkrifvarsArnold W. J. M. Van De LaarMarkus Bernhard BlatzArnoldo Aquino-GálvezMarkus BlaessArnulfo Ramos-JiménezMarkus Florian OertelAroa TardaguilaMarkus KoestenbergerAroa Tardáguila-GarcíaMarkus KoflerAron TendlerMarkus LinhartAron VinczeMarkus MandlArong GaowaMarkus SchoenbergArrigo CiceroMarkus SkrifvarsArrigo FruscalzoMarkus StraussArsalan SalariMarkus VeltenArseniy E. YuzhalinMarkus WahrmannArseniy LobovMarkus WeberArtem KuzovlevMarlena SzalataArtem OvchinnikovMarlene L. DurandArthi ShridharMarlies GijsArthit PhosriMarlijn HoeksArthur C. SilvaMaroš HrubinaArthur De Sá FerreiraMarousa KouvelaArthur FerreiraMarsal SanchesArthur M. MelkumyantsMarta Araujo-CastroArthur OrieuxMarta Baranowska-KuczkoArtur DziewierzMarta Blasco-AlonsoArtur LemińskiMarta CarettoArtur LorensMarta CitelliArtur RebeloMarta CvijicArtur SłomkaMarta Dąbrowska-BenderArtur WdowiakMarta FijałkowskaArturo Flores-PliegoMarta FiscalettiArturo López CastelMarta FocardiArturo Sanchez-PerezMarta Hernández-CondeArun ButreddyMarta IscarArun MeyyazhaganMarta Jóźwiak-BębenistaArun Pandian J.Marta MaleszaArun PrasadMarta MonistArun PrasathMarta Nowakowska-KotasArun RajasekaranMarta OknińskaArun Saravanakumar AnnamalaiMarta OpalińskaArun-Kumar Kaliya-PerumalMarta Pérez-de-Heredia-TorresArvind NegiMarta Ramon-KrauelArvind NuneMarta Regina Cezar-VazArvind Singh ChandelMarta Rojas-GimenezArya AminorroayaMarta RusekAryeh ShanderMarta RybskaArzu BakirtasMarta SupervíaAsaf AchironMarta TajesAsaf LevartovskyMarta VerneroAsaff HonigMarta Waliszewska-ProsółAsbjørn ÅrøenMarta Zaleska-KocieckaAsha TyagiMarta Zapata-TarresAsher BashiriMarta ZolaAsher OrnoyMarten TrendelenburgAshesha MechineniMartha Alejandra Morales-SánchezAshfaq AhmadMartha Eva Viveros-SandovalAshish JainMartha NowosielskiAshish KumarMartín A. MaraschioAshish ManneMartin BalzanAshish NoronhaMartin BergerAshish RunthalaMartin BesserAshlee StilesMartin Bjørn StausholmAshok Kumar BanskotaMartin BommerAshraf Al-BrakatiMartin BorggrefeAshraf KariminikMartin BuschAshraf M. MohieldinMartin Christian MichelAshril YusofMartin Cornelius JordanAshton TheakstoneMartin FrisherAshutosh KumarMartin FunkAshwini YenamandraMartin G. SchermAsim Abu-BakerMartin HelanAsim Bikas DasMartin HerrmannAsitha D. L. JayawardenaMartin J. SwaansAsja ČelebićMartin JanickoAsli AykacMartin John DedicoatAsli OzmenMartin Jozef PéčAsmerom SengalMartin K. AngeleAsnat RazielMartin KrsekAsra Al FauziMartin LoertscherAsri MaharaniMartin M. MortazaviAsser A. SallamMartin ManningerAssumpta PeralMartin MullerAsta LinauskasMartin MynarekAstrid Christine ErberMartin Oliver SchmiadyAstrid J. TerkelsenMartin PohlAstrid Kamilla StunesMartin RakusaAstrid PetersmannMartin RiesenhuberAstrid PinzanoMartin RožánekAta Nevzat YalçinMartin S. LipskyAtari MaherMartin SpitzerAtefeh AbediniMartin StimpfelAtefeh AmerizadehMartin TeraaAthanasia PapazafiropoulouMartin UnverdorbenAthanasia PrintzaMartin VlkAthanasia ProklouMartina BehanovaAthanasia XirogianniMartina BonifaziAthanasios AnastasilakisMartina BrianiAthanasios N. TsartsalisMartina BurlandoAthanasios T. PapadasMartina C. Herwig-CarlAthanasios VergadosMartina De ZwaanAtheer ZgairMartina FabrisAthikhun SuwannakhanMartina GabrielliAthina PyrpasopoulouMartina IrionAthina-Maria AloizouMartina MeszarosAtiyeh AbdallahMartina Rojnic KuzmanAtiyeh M. AbdallahMartina SettiAtsuhito TakedaMartínez CastelaoAtsumasa KomoriMartins IsabelAtsushi FukuiMartti VastamäkiAtsushi FukunagaMartyna SławińskaAtsushi IkedaMaruška MarovtAtsushi IrisawaMarvin Antonio Soriano-UrsuaAtsushi KawaharaMarvin R. BalaanAtsushi SaigaMarwa GaberAtsushi SakamotoMarwa MatboliAtsushi SatakeMarwan El MobadderAtsushi SugiuraMarwan F. JumeanAtsushi SuzukiMary GannottiAtsushi TanakaMary Garland-KledzikAtsushi YamashitaMary HendrixAtsuyuki InuiMary ImbodenAttila FeherMary J. DunbarAttila FrigyMary M. Y. WayeAttila JakabMary N. SheppardAttila OláhMaryam ArdalanAttila RokaMaryam AzlanAtukuri DorababuMaryam FarzadAtul B. MehtaMaryam Mobed-MiremadiAtul BabbarMaryam MoiniAubert AgostiniMaryam TofangchihaAude BelbézierMary-Ann FitzcharlesAude Sophie NassifMarzena LaskowskaAudrey A. Stinghen Garcia LonniMarzena LenartAudrey Cougnard-GrégoireMarzenna ZielinskaAudrey PerryMarzia Adelia LocatelliAudrius DulskasMarzia CottiniAudrius SileikisMarzia GiaccardiAugustin Mouinga-OndéméMarzia SegùAugusto LauroMarziliano NicolaAugustus D. MazzoccaMaša SinreihAura MihaiMasaaki HoshigaAurel BurciuMasaaki KonishiAurélie WagenerMasaaki TakeuchiAurélien JustetMasafumi FunamotoAurélien LorthioirMasafumi ImamuraAurélien LouvrierMasafumi InomataAurelio Luna MaldonadoMasafumi OdaAurora VassanelliMasafumi OnoAveek JayantMasafumi SugiyamaAvi DombMasahiko AyakiAviad Tur-SinaiMasahiko KatoAvik DuttaMasahiko SugimotoAviram HochstadtMasahiko TsuchiyaAvishay ElisMasahiro FunabaAvital MendelsonMasahiro NakayamaAxel BoeseMasahiro OkadaAxel KünstnerMasahiro TajikaAxel SchlittMasahiro TakahashiAxel SchmutzMasahito KatsukiAxel SteigerMasahito NakanoAya SaitoMasakazu HirotaAyako AraiMasakazu MinetamaAyataka FujimotoMasakazu TanakaAydin GülsesMasaki HanibuchiAykut CilliMasaki IzumoAyman ElnahriMasaki KajimotoAyman ElnahryMasaki NagayaAyman ElsamanoudyMasaki OkamotoAyoung JeongMasaki YasufumiAysegul YildizMasakiyo YatomiAytan MusayevaMasako NakanishiAyumi SugiuraMasamitsu HyodoAyushi ChauhanMasanori KawakamiAzamat TemerdashevMasanori KonoAzza Abdel Mohsen El HousseinyMasanori MurakamiAzza MostafaMasanori SaitoAzzawi HadiMasao KodaB. Anthony ArmsonMasao SaotomeB. Keegan MarkhardtMasaru MiyazakiBabak E. SaraviMasaru YamaguchiBabak SabouryMasashi FujinoBabar K. RaoMasashi KawamiBabatunde RajiMasashi NinomiyaBabett RamsauerMasashi YamashitaBagher LarijaniMasatake KobayashiBahadir SimsekMasateru UchiyamaBahar AtaeiniaMasato FujikiBaharudin AbdullahMasato OkadaBahauddeen AlrfaeiMasato YanagiBahil GhanimMasato YoshiharaBaki HekimoğluMasatsugu OruiBala RamananMasaya MukaiBalagangadhar TotapallyMasaya UesatoBalakrishna KoneruMasayuki EndoBalazs FeherMasayuki FujiwaraBaldassarre MartireMasayuki OhtsukaBallambhattu Vishnu BhatMasayuki OzakiBang-Gee HsuMasayuki ShimaBanu Eriş GülbayMascha O. FiedlerBaolei JiaMasfueh RazaliBaptiste DemeyMasoud KeikhaBarbara AntonelliMasoud SadeghiBarbara BarunMasoumeh Kourosh AramiBarbara BauceMassimiliano De PaolisBarbara Bodner-AdlerMassimiliano MangoneBarbara BrognaMassimiliano ScalvenziBarbara CvenkelMassimiliano ToscanoBarbara FazekasMassimiliano VerouxBarbara J. PhilipsMassimo BaudoBarbara JasiewiczMassimo BelliniBarbara KabonMassimo BonacchiBarbara ŁapińskaMassimo CapocciaBarbara Nieradko-IwanickaMassimo CarossaBarbara NikolaidouMassimo ChelloBarbara RemberkMassimo D’AgostinoBarbara Ruszkowska-CiastekMassimo GalliBarbara SargentMassimo IacovielloBarbara SonntagMassimo MangiolaBarbara StreicherMassimo MarrelliBarbara SzczepankiewiczMassimo MascoloBarbara TomasinoMassimo SalviBarbara Wis̈niowskaMassimo TusconiBarbara ZaapalaMassimo ZecchinBarclay T. StewartMatas JakubauskasBard KittangMatei BratuBarış Ethem SüzekMatej MlinaricBarry A. SiegelMatej SamosBarry SwerdlowMatej StrnadBart PijlsMateja Dolenc VoljčBartlomiej BarczynskiMateja SiršeBartłomiej GórskiMateo López-MoralBartłomiej Henryk NoszczykMateusz BabickiBartłomiej KamińskiMateusz GrajekBartłomiej KuleszaMateusz KoteckiBartłomiej PerekMateusz SpałekBartosz CzubaMateusz WichtowskiBartosz ForoncewiczMateusz WiniarczykBartosz KrzowskiMateusz ZawadkaBartosz KubisaMatheus BertanhaBartosz MałkiewiczMathias BrandtBartosz MiziołekMathias ForkmannBartosz SzmydMathias MaierBasama MnifMathias OrbanBaseer U. AhmadMathias TrempBasem B. AbdelmalakMathieu BeraneckBasheer MannasahebMathieu JozwiakBasil K. WilliamsMatias IglickiBasilio De La TorreMatías JuradoBassem I. YamoutMatias SoiferBasu Dev PandeyMatiss OzolsBayram Ali DorumMatjaž KopačBeat RothMats C. RaneboBeata Galińska-SkokMats ErikssonBeata Hukowska-SzematowiczMats LindbladBeata Labuz-RoszakMats SjöströmBeata ŁubiankaMatt RutarBeata Mrozikiewicz-RakowskaMatteo BelliniBeata TarnackaMatteo BerniBéatrice Brembilla-PerrotMatteo BiancoBeatrice CairoMatteo BoattiniBeatrice FemminellaMatteo BolcatoBeatrice GalloMatteo BrucoliBéatrice GulbisMatteo CarpiBeatrice ThielmannMatteo FermiBeatrice VolelMatteo FontanaBeatriz Arranz-MartínMatteo FornaiBeatriz Bañuelos MarcoMatteo FoschiBeatriz Benitez-TemiñoMatteo GhilliBeatriz Buentello-VolanteMatteo Luigi Giuseppe LeoniBeatriz Da C. A. AlvesMatteo ManfrediBeatriz De-Mateo-SillerasMatteo MegnaBeatriz MinghelliMatteo MonticelliBeatriz Silva-RamírezMatteo Monzio CompagnoniBeatriz Villarejo-CarballidoMatteo MoroniBebiana CondeMatteo MuellerBecky D. ClarksonMatteo NardinBeda HartmannMatteo PaciniBedjan BehmaneshMatteo PaganiniBee K. TanMatteo PonzoniBegona EzquietaMatteo RiccòBehrooz MamandipoorMatteo RipaBei ZhangMatteo SacchiBéla MerkelyMatteo SaccucciBela OzsvariMatteo TrimarchiBelal ShohayebMatthew A. EdwardsonBelen Alonso OrtizMatthew E. HirschtrittBelinda De SimoneMatthew J. RegulskiBelinda Pinto-SimõesMatthew J. SmithBella UngarMatthew Joe GrimaBellu LuisaMatthew Q. MillerBen Abdallah IsmailMatthew RohlfingBen MulhearnMatthew SchindlerBenan BayrakciMatthew TuckerBence Tamás SzabóMatthew W. VannemanBenedetta GuaniMatthias BechsteinBenedetto IelpoMatthias D. HoferBenedict B. BenignoMatthias FischerBenedict FrancisMatthias FröhlichBénédicte DélireMatthias HolzbauerBenedikt AsbachMatthias KarlBenedikt HofauerMatthias KempeBenedikt ReutersbergMatthias KolibabkaBenedikt SaglMatthias KorellBenedikt SchwormMatthias MillesiBenito ChiofaloMatthias PortBenjamim FicialMatthias SchulzBenjamin AnsaMatthias SendlerBenjamin BabicMatthias SteinertBenjamin BenzonMatthias UnterhuberBenjamin C. P. LeeMatthias VogelBenjamin DaviesMatthias WeissingerBenjamin E. GarfieldMatthias WoiczinskiBenjamin Howard FreedMatthieu LalevéeBenjamin L. WoolbrightMatthieu PeycelonBenjamin R. OshrineMatthijs Paul SomfordBenjamin R. StripeMatti EskelinenBenjamin VoellgerMattia BrancaBenny BjörkblomMattia D’AgostinoBenoit TessoulinMattia DominoniBenoît TousignantMattia Falchetto OstiBenoit WitkowskiMattia Russel PantaloneBeom Jin LimMatus DurdikBeom-Jun KimMau-Ern PohBerdien W. Van Der LindeMaura M. E. PilottiBérengére KoehlMaurice RuettersBerk BurguMaurice-Andre RecanatiBernadette CoricaMauricio Castro-SepulvedaBernadette Emoke TelekyMauricio Cesar De MarziBernard BégaudMauricio EtchebehereBernard CanaudMaurizio BertainaBernard CosynsMaurizio Cappelli BigazziBernard LeungMaurizio DomaninBernard MaitreMaurizio GabrielliBernard NaafsMaurizio IacoboneBernard Pui Lam LeungMaurizio MarchesiniBernard SpeculandMaurizio NordioBernard T. HaylenMaurizio PascadopoliBernd FritzschMaurizio RicciBernd MetznerMaurizio TonettiBernd SalzbergerMauro BerguiBernd StegmayrMauro GiacomelliBernhard HessMauro GiuffrèBernhard LämmleMauro LombardoBernhard MeyerMauro ManiscalcoBernhard N. BohnertMauro RagoneseBernhard RauchMauro TaralloBernhard SchwabergerMaw-Sheng LeeBernhard W. UllrichMax J. GordonBernward LauerMax KurlbaumBert VandenberkMax MasthoffBerthold DrexlerMax NedelmannBertold RennerMax ReijmanBertrand GuidetMaxime ElbazBertrand LiagreMaxime HentzienBertrand TchanaMaxime NguyenBeth A. SchropeMaxime PichonBeth BockMaxime SamsonBethany PesterMaxime TouzotBetina BiagettiMaximilian Johannes SteinhardtBettina CarvalhoMaximilian PilhatschBettina WinzelerMaximilian Von RoederBeyza Ünalan DeğirmenciMaxwell SuBhagavathi Sundaram SivamaruthiMaya MascarenhasBhama RamkhelawonMayuko Osada-OkaBhanu TewariMayur VirarkarBharani Krishna Mynampati ArunadithyaMazen SalehBharat GurnaniMd AdnanBhaswati BhattacharyaMd Daud Mohd KhairiBhoomika PatelMd Hakimul HaqueBhupesh ThakurMd MohiuddinBhupinder KapoorMd Rabiul IslamBhuvanesh Sukhlal KalalMd. Tanvir RahmanBiadsee AmeenMeaghan CloughBiagio BaroneMeda E. PavkovBiagio SantellaMeda-Lavinia NegrutiuBiagio SassoneMedine Karadağ AlpaslanBianca E. ŐszMeenakshi AhluwaliaBianca GoemansMeer M. ChisthiBianca Laura CinicolaMegan L. SulcinerBianka ForgoMegan PaulsenBibek PoudelMeganathan KannanBibhusal ThapaMeghal GagraniBibhuti B. DasMeghan C. McCormickBibiane Steinecker-FrohnwieserMeghan McGee-LawrenceBieneke JanssenMegumi KishimotoBilal Haj NajeebMehdi MoghaddasiBilal MirMehdi MontazerBin ChenMehdi SakkaBin DuMehmet Ali ErgünBin SongMehmet Ali KobatBin ZhangMehmet Ali TokgozBing SunMehmet BaysalBing YangMehmet Fatih ŞentürkBingqing XieMehmet GülBingwen Eugene FanMehmet KocaogluBinila ChackoMehmet N. OrmanBinita ShresthaMehmet SarierBipin Kumar SethiMehran AkselBirgit HabedankMehrdad EmamiBirgit LangeMehrez ElnaggarBirkan BozkurtMei FengBirna Bjarnason-WehrensMeihua JiBirnbaum YochaiMeijuan ZouBirte WeberMeinrad BeerBirute StrukcinskieneMelania M. AmorimBita GeramizadehMelania MaglioBjoern VogtMelanie E. BoeyerBjörn NashanMelanie HagenBjörn SommerMelanie HaymanBjörn StesselMelanie J. SekeresBjörn TampeMelanie JanningBlair UniackeMelanie LengerBlanca De-la-Cruz-TorresMelanie WalkerBlanca Hernández-CruzMelike EmiroğluBlanca Lizaola-MayoMelis OlcumBlandine LaferrereMelis PalamarBlazen MarijicMélissa CôteBob PhillipsMelissa GrigorescuBobirca FlorinMelissa J. AlldredBogdan BatkoMelissa J. WalkerBogdan DorofteiMelissa L. CullimoreBogdan Marian SorohanMelissa MarianaBogdan ObrzutMelita Vukšić PolićBogdan PavelMeliza Goi RoscaniBogdan Silviu UngureanuMenelaos N. ManoussakisBogna Grygiel-GórniakMeng C. LinBogusz Aksak-WąsMeng YinBojan JoksimovicMeng-Han TsaiBojan KnapMeng-Huang WuBojan PajicMengxi ShenBong-Hyeon KyeMenicanti LorenzoBonhyuk GooMenno HoekstraBon-Wook KooMenno R. GermansBoohwi HongMentore RibolsiBoon Lead TeeMeraj Alam KhanBoon-Huan TanMeral SertelBoopathi SubramaniyanMercante GiuseppeBoris Filipović-GrčićMercedes Bueno-CampañaBoris SchmitzMerica Glavina-DurdovBoris SlobodinMeriem BelheouaneBorja Mora-PerisMeritxell IbernonBortolo MartiniMervyn J. EadieBorut JugMetehan ImamogluBorys FrankewyczMetin ÇalışırBorys PrzemyslawMettu Srinivas ReddyBoyan FangMevlüt ÇelikBo-Yie ChenMexhid FeratiBo-Young HongMia RamklintBoyoung JoungMicah ZuhlBožana ČolovićMicha Tobias MaederBožena Ćurko-CofekMichael A. E. RamsayBożena SobkowiczMichael BenderBożena Szyguła-JurkiewiczMichael BouvetBradicich MatteoMichael BullardBradley T. SmithMichael C. McDanielBram JacobsMichael C. SoulenBrandon G. SmagloMichael ChoiBrandon VanderVeenMichael ChristBrandt C. WibleMichael ColemanBranislava A. MilenkovićMichael D. DiamantidisBranka Petrić-MišeMichael DevaneBranka PolicMichael DonahoeBrega CarlottaMichael DoulberisBrenda KazemierMichael DrozdBrenda LakyMichael EderBrendan EckMichael EfremidisBrendan Joseph McMullanMichael EichingerBrent SieskyMichael EwerBrent WagnerMichael F. YayacBrett HamblyMichael FeichtingerBrian AnnexMichael FrassBrian BellMichael FroschBrian C. CaseMichael Garnet IrwinBrian CurtisMichael GembickiBrian D. LowesMichael HardistyBrian K. FoutchMichael HeneinBrian Kwok Hong LawMichael J. GermainBrian S. ApplebyMichael J. KoziolekBrian S. W. ChongMichael J. KrowkaBrian ShawMichael JoachimBrian VestalMichael Kleiner ShochatBrice T. ClelandMichael KnitschkeBridget AtkinsMichael L. BernardBrigida IorioMichael L. CherBrigitte KaufmannMichael L. KuehtBrigitte M. OhlmannMichael MillroseBrigitte StrizekMichael NekludovBrigitte WilczekMichael OeverhausBrijesh TakkarMichael OlsonBrijeshkumar PatelMichael P. ReichelBritni L. AyersMichael P. VallelyBritt OpdebeeckMichael PalaciosBrittany G. SemanMichael PeerBrittany K. TaylorMichael PinkawaBrittany MurphyMichael R. CragerBrittany N. KrekelerMichael R. JacobsBritt-Isabelle BergMichael R. KingBrittney J. SchultzMichael R. NarkewiczBronisława Skrzep-PoloczekMichael RankeBruce Jeffrey RogersMichael ReinerBruna EibelMichael RosenzweigBruna Santos Da SilvaMichael RubartBrunella BagattiniMichael RudnickiBruno BaršićMichael S. KaminerBruno ChrcanovicMichael SchirmerBruno FiondaMichael SchwaigerBruno García-Del-BlancoMichael Scott FreemarkBruno José Nievas SorianoMichael SingletonBruno Kusznir VitturiMichael Stephan GruberBruno Lovaglio Cançado TrindadeMichael SwansonBruno PassarettiMichael T. HreskoBruno PavyMichael T. KoltzBruno Ramos-MolinaMichael Tobias NeuhausBruno RaynardMichael TronnierBruno SalvatiMichael TsatsosBryan D. HujsakMichael V. JoachimBryan GarciaMichael WeitzendorferBryan M. TuckerMichael William MatherBryan YoungMichael YimBryce BairdMichael YoungBryce BinstadtMichael ZeilerBülent KaradagMichael ZyskowskiBum-Yong KangMichaela FiliouBurak ArslanMichaela Shishmanova-DosevaBurak BuldurMichail DiakosavvasBurak YulugMichail MavrosBurghard J. AbendsteinMichail SorotosBuse Ozcan KahramanMichal A. RahatBushrah Binti BasironMichał BijakByoung Sun ChuMichał BoraczyńskiByoung-Ho LeeMichał BorysByron AsimakopoulosMichał ChmielewskiByron VaughnMichal ChowersByuk Sung KoMichał ChyrchelByung Eui KimMichal CiebieraByung Jun ChoMichal CiurzynskiByung Yi KoMichał CzaplaByunghyuk YuMichał GinsztByung-Kwan SeoMichal GurByungok ChoiMichal KorostynskiByung-Wook KimMichal KozlowskiC. Andrew CombsMichał KunickiC. Bulai LivideanuMichał KuszewskiC. J. AlvesMichał LatalskiC. M. Sabbir AhmedMichał LewandowskiC. Sieiro SantosMichał ŁobaczC. ThibeaultMichał MaćkowskiCailu LinMichał MączewskiCaio H. FrancoMichał NowickiCaitlin TakahashiMichal OrdakCalin CorciovaMichał P. PlutaCalogero VetroMichał PomorskiCalvin EiberMichał RabijewskiCalvin YeangMichal S. NowakCamelia CoadaMichał StasiowskiCamelia ProtopopescuMichał SzczyrekCameron W. MacDonaldMichal VágnerCamila TirapelliMichal VostrýCamilla Albertina Dantas De LimaMichał ZarobkiewiczCamilla Biering LundquistMichał ŻorniakCamilla HageMichał-Goran StanišićCamilla RussoMichalis GalanopoulosCamille MaloufMichel I. A. J. WyndaeleCan KoyuncuMichel MondainCan TuygunMichel RoethlisbergerCandelaria Martín-GonzálezMichel ToupetCandelas Pérez Del VillarMichela AmatrudaCândida Teixeira TomazMichela Di TraniCao YangMichela GabelloniCara SaxonMichela IannoneCarina Carvalho SilvestreMichela PiezzoCarina MalmhällMichela RauseoCarl ArndtMichela RelucentiCarl ErbMichela TerlizziCarl FisherMichelangelo La PlacaCarla Busquets-CortésMichelangelo LucianiCarla FeliceMichelangelo ScaglioneCarla Giuseppina CortiMichele AltomareCarla Maria Batista Ferreira PiresMichele AntonelloCarla OlivieriMichele AriglianiCarla PagliariMichele BoffanoCarla PelusiMichele CampigottoCarla RodriguesMichele ColonnaCarla Sanchis-SeguraMichele CorrealeCarla Sílvia FernandesMichele CostanzoCarlo Alberto MaroneseMichele CovelliCarlo BarausseMichele CovielloCarlo BizMichele D’AttilioCarlo BrognaMichele Di CosolaCarlo CaiatiMichele FinottiCarlo CernettiMichele FioreCarlo Di PaoloMichele GolinoCarlo GandiMichele LanzaCarlo L. RomanòMichele LonghiCarlo LavalleMichele MaglioneCarlo MessinaMichele MagnocavalloCarlo PerisanoMichele MazzolaCarlo RostagnoMichele MercurioCarlo VignatiMichele PaolantonioCarlos AbreuMichèle PreydeCarlos Alberto Fontes RibeiroMichele ProvenzanoCarlos Diaz-ArocutipaMichele RizziCarlos DurántezMichele SalemiCarlos Eduardo Fonseca-AlvesMichele Schiano Di VisconteCarlos Eduardo FontanaMichele SicaCarlos Fontes RibeiroMichele SilvestroCarlos GalanMichele UmbrelloCarlos Gonzalez-JuanateyMichele ZiniCarlos H. HirokiMicheline R. AndersonCarlos H. RomeroMichelle L. MillerCarlos Ignacio Ponte-NegrettiMichelle M. Kameda-SmithCarlos J. MarquesMichelle NisolleCárlos L. Ayán PérezMichiel F. G. De MaatCarlos LamMichikazu NakaiCarlos LifschitzMichiko MandaiCarlos Llanes-ÁlvarezMichio HongoCarlos Manuel Ortíz-MendozaMichiyo YamanoCarlos MarquesMickaël BobotCarlos Martín ArdilaMickael BonnanCarlos Martin LlorenteMickael EssoumaCarlos Moreira-NetoMicol FalabellaCarlos Navarro CuéllarMielnik MichalCarlos O’Connor-ReinaMieszko WieckiewiczCarlos OpazoMieszko WięckiewiczCarlos PradaMiguel A. Garcia-GonzalezCarlos Rocha-De-LossadaMiguel A. SimónCarlos Salavera BordásMiguel AlcarazCarlos TrescolíMiguel Angel Alcántara-OrtigozaCarlos VareaMiguel Ángel Canales AlbendeaCarlotta CervaMiguel Ángel Castro VillamorCarlotta SenniMiguel Angel RubioCarmela RinaldiMiguel CardosoCarmelo CaldarellaMiguel Castelo-BrancoCarmelo FerraiMiguel De PedroCarmelo GurnariMiguel Gomes GuerraCarmelo Luca SmeraldaMiguel LitaoCarmelo SaranitiMiguel Nobre MenezesCarmen AcevesMiguel Soares‐OliveiraCarmen BerenguerMiguel TeusCarmen Ferragut FiolMiguel Urina-TrianaCarmen FigueroaMihaela GhitaCarmen González EnguitaMihaela HedeșiuCarmen MannellaMihaela HostiucCarmen Méndez HernándezMihaela IonescuCarmen Moret-TatayMihaela MocanCarmen PanaitescuMihaela PanteaCarmen SavinMihaela PerteaCarmen SerranoMihaela TurtoiCarmen Sílvia Valente BarbasMihaela VladCarmen Soria-SegarraMihai CapilnaCarmen Sorina MartinMihai CraiuCarmen SticlaruMihai Dorin VartolomeiCarmine ConteMihai DumitruCarmine IzzoMihai GeorgescuCarmine NovielloMihai JuncarCarmine PiconeMihai LupuCarmine PizziMihai MusteataCarol BurnsMihai OlteanCarola TuerkMihai PopescuCarole C. UpshurMihai RomanCarole DeanMihai StefanCarolin HoyerMihai Stefan MuresanCarolina Alonso MonteroMihai StrachinaruCarolina DonàMihnea MarinCarolina RojasMiho MurashimaCarolina SerenaMi-Jung KwonCarolina Vila-ChãMika L. NonoyamaCaroline C. JefferyMika VenojärviCaroline HastingsMikael AbergCaroline J. VranaMike DoddCaroline Nervil KistorpMikel San-JuliánCaroline Sévoz-CoucheMikel Vicente-PascualCaroline SubraMikhail A. DziadzkoCarolyn D. KramerMikhail EfanovCarolyn DunfordMikhail M. KostikCarolyn R. BatesMiki KushimaCarpén TimoMikio KajiharaCarrol SaridinMikkel M. RasmussenCarsten GrötzingerMiklós Dénes ReschCarsten TjellMiklós FagyasCasper Glissmann NimMi-Kyoung ChoCassandra E. HendersonMilad SharifpourCassia RighyMilan DrobacCatalin BulaiMilan LepicCatalin G. HerghelegiuMilan PrsaCatalin TiliscanMilan SkitekCatalina De Paco-MatallanaMilena ŚwitońskaCatalina LucaMiles WeinbergerCatalina MihaiMilica DeklevaCatarina GodinhoMilica JankovicCatarina RuaMilica Pejovic-MilovancevicCatarina Santos De SousaMilica ZekovicCatarina TomásMilorad DragićCaterina BensiMilorad TesicCaterina BuffoneMilos LazarevicCaterina DelceaMiłosz CabanCaterina LeoneMilton KanashiroCaterina Maria GambinoMin Hyun ChoCaterina NeriMin JungCaterina StornelloMin PengCaterina TomaMin Soon ChoCatharina Sagita MoniagaMin XieCatherine BungenerMin Young LeeCatherine Caldwell-HarrisMinal ThackerCatherine Creuzot-GarcherMine Gürsaç ÇelikCatherine DavisMinerva Codruta BadescuCatherine KlersyMinerva Gomez-FloreCatherine M. SuttleMing ChenCatherine PaillardMing YangCatherine PenningtonMing-Chang KaoCatherine S. SpinaMing-Chen Paul ShihCatherine WillshireMing-Chin YuCathleen N. BrownMing-Fen LeeCathy A. Johnson-DelaneyMinghang WangCathy PedersenMing-Horng TsaiCátia MagalhãesMing-Hsin YangCécile OuryMing-Hui SunCécile TomowiakMing-Jen LeeCecile VuillermozMingjian SunCecilia Alegado JimenoMing-Ju WuCecilia BindaMing-Jui HungCecilia ChanMingming TongCecilia Gómez RavettiMing-Ping WuCecilia PerinMingshun HsiehCecília RajdaMing-Tse KuoCecilie RøeMingwei LaiCedric HermansMingxing WuCédric RossiMing-Yen LinCees A. SwenneMing-Ying LanCeleste ManfrediMin-Ho HanCelestia S. HiganoMin-Hsien ChiangCelestino SarduMin-Huan WuCelia HardingMinhui GuanCelia Ruiz-SánchezMin-Hui LiCelia Sánchez-RamosMinjee LeeCelina AngMinoru OsanaiCéline ClémentMin-Wook KimCeline KempeneersMinYoung KimCélio José Castro-JuniorMiodrag FilipovicCem SimsekMiodrag JanicCemal UcerMiquéias Lopes-PachecoCemil ColakMiquel KraftCengiz KorkmazMira VukovićCesar MartinMircea Ioan PopaCésar PicadoMircea TampaCezary WatałaMircea-Catalin FortofoiuCezary WójcikMireia TondoChad L. CrossMirela Cleopatra TomescuChae Moon HongMirela FrandesChaeseong LimMirela Pavicic IveljaChahnez Charfi TrikiMirela RaosChahyun OhMirela TiglisChaitanya GadepalliMirella Telles Salgueiro BarboniChambers ShirleyMiriam F. ScelzaChan ParkMiriam FrenkenChan Rok ParkMiriam Keltz PomeranzChanchal Deep KaurMiriam PikkemaatChandrakant LahariyaMiriam WeisskopfChandrashekhar VoshavarMirian Santamaría-PeláezChang Dong YeoMirja TenhunenChang HuangMirjana BjelošChang Hyun KangMirjana JerkicChang-Myung OhMirjana RumboldtChangquan LingMirko BelliatoChangwei WeiMirko DuradoniChangwon JeongMirko ManettiChangzhen WangMirko Parasiliti-CaprinoChanon NgamsombatMirko PretoChao HeMirko ResanChao ZhangMiroslav P. IlićChaochao ZhouMirosław BrykczyńskiChaowalit YuajitMirosław GiurgChao-Yu ChenMiroslaw JanowskiChara GeorgatzakouMiroslaw RajdaCharalampos PierrakosMirosław ŚwierczCharat ThongprayoonMirsala SolakCharikleia KelaidiMirto FolettoCharitha GowdaMiruna Carmen BarbuCharles A. ReismanMirza PojskicCharles C. CaldwellMirza R. BaigCharles C. CoddingtonMisagh PiranCharles FauvelMisako NagasakaCharles H. ThivoletMisao NishikawaCharles NatansonMishall Al-ZubaidieCharles StauftMishra Vikash ChandraCharles VerneyMislav MikusCharles William YoxallMistuhisa TakatsukiCharlotte SimonÿMi-Sun HurChathur AcharyaMital ShahChatree Chai-AdisaksophaMitat KozChawla KiranMithalesh Kumar SinghChaya ShwaartzMithra Nidharsh HegdeChe Matthew HarrisMitja RuprehtChellappagounder ThangavelMitra AkbariChen ChuMitra TavakoliChen Liang TsaiMitsuaki NishikimiChen YuMitsugi ShimodaChen ZhouMitsuhiko KuboChen ZhuMitsuhiko TakahashiChen-Cheng YangMitsuhiro NitoChenchih ChungMitsuhiro TakeuchiChendong HanMitsuro ChibaCheng Hong TsaiMitsuru IdaCheng XueMitsuru NishiyamaCheng Yin TanMitsuru OhishiCheng-Hsien WuMitsuru OkunoCheng-Jen ChangMiya YoshinoCheng-Jun HuMoawiya HaddadChenglei FanMobeen RehmanChengli QueMohamad J. AlshikhoCheng-Shun LuMohamad MokademCheng-Tang ChiuMohamad RahoumaChengting HoMohamed A. HamidCheng-Tsung HsiaoMohamed AliCheng-Wei LiuMohamed BalahaChengyuan WuMohamed BoussarsarChen-Hua LiuMohamed El FarissiChenjin JinMohamed El KassasChenjuan GuMohamed El-MeseryChenlu GaoMohamed El-RaiyChen-Pang HouMohamed ElrayessChenqi TaoMohamed El-SherbinyChen-Xiong HsuMohamed HassanCherie LeeMohamed K. MostafaCherng Ru HsuMohamed Mohamed ElawdyCheukkwan SunMohamed RafiullahCheung-Ter OngMohamed SallaChe-Wei ChangMohamed W. AttwaChewTeng KorMohamed ZakyChe-Yuan KuoMohammad AkbariChi Chun WongMohammad AlamChi MaMohammad Al-Mahdi Al-KaragholiChia-Che LeeMohammad Al-WardatChia-Chi YangMohammad Esmaeil AkbariChiaki ToidaMohammad Esmaeil BarbatiChialung ShihMohammad EtoomChiara BardellaMohammad HajeerChiara BecchettiMohammad HossainChiara CantarelliMohammad Hossein KhosraviChiara CastellaniMohammad ImranChiara CazzorlaMohammad JavanbakhtChiara ChieregoMohammad KassarChiara CogliatiMohammad Kermani-AlghoraishiChiara ColuccioMohammad KhalifehChiara De BiaseMohammad Mahbubur Rahman Khan MamunChiara Di RestaMohammad MohammadianpanahChiara DonadeiMohammad Mostafa Ansari-RamandiChiara GiuffrèMohammad Muzaffar MirChiara Giulia FontanellaMohammad Nazmul HasanChiara Maria MazzantiMohammad RababaChiara MarroneMohammad Reza RouiniChiara MartiniMohammad Sultan AlakhaliChiara MusiuMohammad ZmailiChiara PaoliniMohammad ZulkifliChiara PellicanoMohammadreza SalehiChiara SaraciniMohammadreza ShalbafanChiara SorberaMohammed AlasawshChiara VeneroniMohammed DiykhChia-Ti TsaiMohammed GrawishChien Chih WangMohammed Oday EzzatChien-Chia HuangMohammed Rajik KhanChien-Chung KuoMohammed RohaimChien-Hsien YehMohan Kumar KalaiahChienhsiu HuangMohan RudrappaChien-Jen HsuMohana MaddulaChiensheng HuangMohaned EgredChigozirim N. EkekeMohanraj ThangamuthuChih Feng YenMohd Dzulkhairi Mohd RaniChiharu OtaMohd ImranChih-Che LinMohd Noor NorhayatiChih-Hao ChangMohd Suhail AkhterChih-Hao ChenMohit PahujaChih-Hsin HsuMohit SharmaChihiro AoshimaMohmed Isaqali KarobariChi-Huang ChenMohsen BakouriChih-Wei TsengMohsen KerkeniChihwen WangMohsen YazdanianChih-Yu ChiMoin Uddin AhmedChih-Yuan LinMoïse CoëffierChi-Lun LinMoises BetancortChin Ann Johnny OngMoito IijimaChin LinMojca UrbancicChina NaganoMojgan AlaeddiniChing Feng WengMojtaba AnsariChing Ju ShenMojtaba VaismoradiChingfen YangMona M. El RefaeyChing-Hu ChungMona P. TanChing-Hui SiaMona Seyed SafizadehChing-Sheng HsuMona VintilaChing-Ta LuMoneisha GokhaleChi-Nien ChenMong-Wei LinChinmay ChakrabortyMonia GarofoloChinmaya MahapatraMonia MarchettiChin-Su LiuMonica BoirivantChintan V. ShahMonica De GaspariChin-Yu LinMonica Livia GheorghiuChip HahnMonica MarcChitaranjan MishraMonica MoneaChi-Ting HorngMonica RusticeanuChi-Wei LinMonica Steluta MarcChong-Chi ChiuMonica ValentovicChongyang DuanMonica VerdoiaChor Cheung Frankie TamMonika Christine Brunner-WeinzierlChris A. GentryMonika FleckensteinChris H. L. LimMonika FranczukChris H. L. LimMonika GrabiaChris Hong Long LimMonika KlimekChris HullMonika Klinkhammer-SchalkeChris JonesMonika KniotekChris KourekMonika KoziełChris LabakiMonika KujdowiczChris PeachMonika LejmanChris ReutelingspergerMonika Lukomska-SzymanskaChrissa SiokaMonika MartiniakováChristel WeissMonika MichalakChristelle Le DantecMonika Michalek-ZrabkowskaChristelle MonvilleMonika Morawska-KochmanChristiaan F. MooijMonika Obara-MoszyńskaChristian A. PogodaMonika PechhackerChristian ApelMonika PrendeckaChristian BeltzerMonika ProbstChristian BergerMonika RauChristian BrandtMonika RuszałaChristian Calvo-HenriquezMonika SobocanChristian CamenzuliMonika SzafarowskaChristian CordanoMonika ValtinkChristian DrouetMonika Zawadka-KunikowskaChristian DualéMonique AucoinChristian EhrnthallerMonserrat Olea-FloresChristian F. FreyschlagMontone Rocco AntonioChristian GisselbrechtMontree TungjaiChristian GrebmerMontserrat Puig LlobetChristian Gunge RiberholtMonzurul RoniChristian HöhneMoon-Soo LeeChristian LiebschMoraitou DespinaChristian LottspeichMordechai PollakChristian M. H. R. De GeyterMorena Luigia RoccaChristian MackeMorgan D. SalmonChristian MahnkopfMorgan Le GuenChristian PaechMorgan R. FiresteinChristian Patrick SchaafMorihisa HirotaChristian PlaassMorihito TakitaChristian RustenbachMorin LangChristian SelingerMoritz C. DemlChristian TanislavMoritz DrefsChristian VogeleyMoritz FlickChristian Von RüdenMoriya IwaizumiChristian WunderMorohoshi TakaoChristiane AlbrechtMorris BellChristiane GasperiMorsi KhashanChristilla Bachelot-LozaMorten Baltzer HoulindChristin B. DeStefanoMorten C. MoeChristin HeneinMorten HesseChristina AdamichouMorteza Fallah KarkanChristina Anna StratopoulouMoshe Rav AchaChristina BothouMoshe SalaiChristina Gerth-KahlertMossab Y. SaeedChristina GrupchevaMotoki OkamotoChristina H. WolfsbergerMotoyuki UmekawaChristina MejersjöMouadh BarbirouChristina Oetzmann Von SochaczewskiMouhamed Djahoum MoussaChristina PflugMoyo Chikondi KruytChristina VillardMubarak Taiwo MustaphaChristina WeiseMuh. Imam AmmarullahChristine C. CallMuhammad A. JawadChristine DetrembleurMuhammad Asif NaveedChristine KochMuhammad FarooqChristine L. ChanMuhammad Ikram UllahChristine LehnerMuhammad ImamChristine M. GoodbodyMuhammad IrfanChristine Yoshinaga-ItanoMuhammad Junaid FarrukhChristodoulos E. PapadopoulosMuhammad Khalis Abdul KarimChristof RöösliMuhammad KhurramChristofer BesterMuhammad M. FurqanChristofer LundqvistMuhammad N. MahmoodChristoph ArtznerMuhammad Phetrus JohanChristoph BorzikowskyMuhammad Salman FaisalChristoph BurgerMuhammad Sohail KhanChristoph EllenbergerMuhammad UmairChristoph FaschingerMuhammed AltınışıkChristoph FriedliMuhammed GerçekChristoph G. DietrichMuhammed Sedat SakatChristoph JochumMuhammet İrfan DönmezChristoph P. HornikMuharrem AkinChristoph RiethmüllerMukhyaprana PrabhuChristoph SinningMukul VijChristoph SpangMukund SrinivasanChristoph WohlmuthMulyoto PangestuChristophe BeylsMuneeswar NittalaChristophe DelclauxMunehisa ShinozakiChristophe DurantonMurali ShyamsundarChristophe MassetMuralidharan ManiChristophe OrssaudMuralikrishna LellaChristopher A. ButtsMurat AladaǧChristopher BoehlkeMurat Şakir EkşiChristopher C. DonnellMurat ToprakChristopher E. BauerMuriel ElhaiChristopher E. GrossMurillo-Gómez FabiánChristopher E. PedigoMusaddique HussainChristopher F. LiuMusheer AljaberiChristopher J. LettieriMustafa AkkayaChristopher J. WorsnopMustafa SevindikChristopher J. YoungMustafa Vakur BorChristopher KobierzyckiMustapha KrimChristopher L. McDonaldMuthu SathishChristopher M. KappMuthukumaran JayachandranChristopher M. MaulucciMutsuaki EdamaChristopher MarshallMuyun XuChristopher N. KaufmannMuzamil Yaqub WantChristopher NewmanMyriam HadjouniChristopher PastrasMyriam M. Altamirano-BustamanteChristopher R. M. AsquithMyrto PapamentzelopoulouChristopher ReganMyung Goo LeeChristopher RocheMyung-Jin ChaChristopher SjowallN. I. VolkovaChristopher TaylorN. LeibigChristopher TenchNabeel AlmotairyChristopher WassonNabil A. HasonaChristos ChatzakisNabila BrihmatChristos ChatzikyrkouNachiappa Ganesh RajeshChristos FeloukidisNack-Hwan KimChristos K. YiannakopoulosNada AndelicChristos KontovounisiosNada SallamChristos MikropoulosNadav LevingerChristos TsagkarisNadege ZanouChrysanthi KarapantzouNadezhda GumanovaChrysanthi P. PapageorgopoulouNadhir HammamiChryso Pefkaros KatsoufisNadia CattaneChryssa BekiariNadia Judith Jacobo-HerreraChuangyu WenNadin ElsayedChuanming LiNadine ConradiChuanming XuNadine S. AguileraChui Ping OoiNadine SuffeeChun Hung ChuNafisa JadavjiChun LiangNaga Swetha SamjiChun Woo ChoiNaga Venkata Sabarinath NeerukondaChun-An ChengNagano AyanoChunbin ZouNagaraja S. BalakathiresanChunchia ChengNagib DahdahChun-Fu LiuNahyoung Grace LeeChung-Hui LinNaibedya ChattopadhyayChung-Jung LiuNaina M. P. MaideenChung-Ming ChenNaji KharoufChung-Sung LeeNajib HaboubiChung-Te LiuNajib RahmanChun-Gu ChengNakano YusukeChun-Hou LiaoNam D. TranChun-Hsin ChenNam HuynhChun-Ming ChenNamhee KimChun-Po YenNamie Okino SawadaChun-Song YangNamjoon YiChun-Yu LinNan JiangChushuang ChenNana Kwadwo BiritwumChutima JalayondejaNancy E. LaneChutima KunacheewaNancy Foldvary-SchaeferChu-Yun LuNancy Monroy-JaramilloCiavoi GabrielaNancy S. YounisCicero ArrigoNancy SaadCid André Fidelis De Paula GomesNandini DuggalCiesla MaciejNandini NairCigdem AlkanNandini VenkateswaranCihan KayaNanlin WuCila UmatNaoharu YagiCindy H. S. LeeNaohiko ImaiCindy TraboulsiNaohiro ShiojiCinzia FabriziNaoki KondoCinzia TrumelloNaoki SatoCiprian DanielescuNaoki TeradaCiprian RoiNaoki TojoCiprian TomuleasaNaoki UmemuraCiro EspositoNaoki YamamotoCiro ImbimboNaoko KatsuradaCiro Pasquale RomanoNaomi A. PorretClaire D. JamesNaomi WeintrobClaire De MoreuilNaomi YagiClaire DemiotNaosuke YokomichiClaire DupuisNaoto NishizakiClaire PearsonNaoya HasegawaClaire-Marie RangonNaoya KakimotoClara BonanadNaoyuki MatsumotoClara CenaNaoyuki MiyashitaClara CerratoNaoyuki OtaniClara Llorens-QuintanaNapoleon Torres-MartinezClara Martínez PérezNapoleon WaszkiewiczClara O. SailerNarasimha Reddy SooraClare O’DonnellNarayan GandedkarClarence KhooNarayanaswamy VenketasubramanianClauden LouisNarcís MasollerClaudia DettoriNarcis Octavian ZãrnescuClaudia DittfeldNardos AbebeClaudia DurantiNaresh BummaCláudia FarinhaNaresh KewalramaniClaudia FilippiniNaresh KumarClaudia GiavoliNaresh Kumar RavichandranClaudia Herrera-SiklodyNariman NezamiClaudia KedorNaris KitnarongClaudia MehedintuNark-Kyoung RhoClaudia PalumboNaro OhashiClaudia PisanuNarongchai AutsavaprompornClaudia Ramírez-RenteríaNarut PrasitlumkumCláudia Regina Furquim De AndradeNaseer AhmedClaudia SenglerNaside MangirClaudia SommerNasim NaderiClaudia StöllbergerNasir Muwfaq YounisClaudia TodaroNasrul WathoniClaudia TorinoNatale Salvatore BonfiglioClaudine WulfmanNatalia A. BezdenezhnykhClaudio Bascour-SandovalNatalia A. MuralevaClaudio CannistraNatália AraújoClaudio CappelliNatalia Díaz-ValdiviaClaudio CerchioneNatalia DowgiałłoCláudio Daniel CerdeiraNatalia DubrowinskajaClaudio De LazzariNatalia E. Fares-OteroClaudio FeoNatalia GeppeCláudio MaiaNatalia GrubaClaudio SpinelliNatalia I. KuryshevaClaudio TirelliNatalia Ivanovna AgalakovaClaudio VelatiNatalia MadetkoClaudio VerusioNatalia MazanowskaClaudiu C. PopescuNatalia MotasClaudiu CottaNatalia PawlasClaudiu MorgovanNatalia PerunovaClelia MarmoNatalia RakislovaClémence BonnetNatalia S. Causada CaloClemens FeistritzerNatalia StepanovaClemens KöstersNatalia Świątoniowska-LoncClemens M. RosenbaumNatalia TibertiClemens RosenbaumNatalia TrpchevskaClemens SchererNatalie EnninghorstClément BarsNatalie H. MatthewsClement MedrinalNatalie HomerClemente Maria IodiceNatalie V. ScimeClementina López-MedinaNatalie Van NiekerkClementine LabroscianoNatalija VedmedovskaClifton CallawayNataliya YaglovaClive KellyNatalya GlushkovaCody AshbyNatarajan AyithanCody PeerNataša BratinaColeman I. SmithNatasa MihailovicColin CooperNatasa MilosevicColin H. QuinnNatasa PopovicColleen AldousNatasa Vidovic ValentincicColleen HadiganNatasha A. WeaverColleen M. GallagherNathalia PadillaColleen M. McDowellNathalie Dhed́inColm O’MorainNathalie VermeulenColton J. LadburyNathan J. BrownConcepción De Hita CantalejoNathan R. TreffConcetta Di NoraNathan T. ZwagermanConcetta FedericoNathaniel Aviv CohenConghua WenNathaniel H. RobinConnie C. ShaoNato DarchiaConnie P. C. OwNattawat KlomjitConnor ZaleNattiya TeawtrakulConstance Tom NoguchiNauder FaradayConstantin T. LucaNavaneeth Krishna Rajeeva PandianConstantina AggeliNavapon TechakriengkraiConstanza AlvisiNavdeep GognaCoralie GandreNaveed SamiCoralie L’OllivierNaveen KumarCorentin PangaudNavid Jubaer AyonCorey J. HayesNazanin Ebrahimi-AdibCorina Aurelia ZugravuNazanin MojtabaviCorina Marilena CristacheNazarii KobyliakCorina Mihaela GruescuNazario FoschiCorinna EngelNazif ElaldiCorinna LebherzNderitu PaulCornelia BalaNeagu NicolaeCornelia BetschartNeal M. DaviesCornelis P. Van De VenNebojsa PavlovićCorneliu BolboceanNebojsa S. ManojlovicCorrado BernasconiNeda HakimihaCorrado CarbucicchioNeel B. ShahCorrado CiattiNeelesh Kumar MehraCorrado GizziNeeraj SidharthanCorrado PelaiaNeerav A. DesaiCorrado TagliatiNeerja BhardwajCorstiaan A. Den UilNeetu SinghCortney Wolfe-ChristensenNeftali Eduardo Antonio-VillaCosimo GallettiNegin GhamsarianCosimo NardiNehaben A. GujaratiCosimo SpertiNehal ElsherbinyCosmin CituNehal F. HassibCosmin EneNeil D. MerrettCostantino BalestraNeil LagaliCostantino MancusiNeil TandayCostanza Di ChiaraNejc PikoCostas PantosNejc UmekCourtney Gray MontgomeryNelly Schulz-WeidnerCraig DepkenNelson Andrade-GonzálezCraig ForrestNelson BrownCraig H. KennedyNenad LakusicCraig R. BaileyNenad LakušićCraig WildeNenad VukojevićCrescenzio SciosciaNerea AlonsoCrina Julieta SinescuNeriman İnançCris P. LantingNerina DenaroCristi TartaNery García PortaCristián A. AmadorNesrein HashemCristian ArzolaNessr Abu RachedCristian BaicusNéstor José Martínez-HernándezCristian CastilloNéstor MartínezCristian DeanaNestor R. VillamizarCristian DelceaNeusa Yuriko Sakai ValenteCristian FioriNeuza DominguesCristian FurauNeveen HamdyCristian IndinoNeville VassalloCristian Iulius SurcelNevo MargalitCristian MirvaldNgoc Thang BuiCristian PersuNguyen Minh DucCristian PodoleanuNial QuineyCristian StătescuNiall M. H. McLeodCristian TrambitasNianye ZhengCristian TrâmbițașNiccolò BoniniCristiana GonçalvesNiccolò LombardiCristiana Iulia DumitrescuNicholas BarrCristiane Bitencourt DiasNicholas Faure WalkerCristiane Villela-NogueiraNicholas J. ThomasCristiane Yumi Koga-ItoNicholas J. ThompsonCristiano AmarelliNicholas James CampionCristiano BalzanelliNicholas Vyner ToddCristiano GarinoNick DandCristian-Radu JecanNick Platon SachinisCristina Anna Maria Lo IaconoNick SpindlerCristina BallaNico Golfrè AndreasiCristina BarosaNico Maximilian JandlCristina BostanNico TeskeCristina CapatinaNicola Alberto ValenteCristina CiucaNicola BaldiniCristina DaiaNicola BartolomeoCristina Floriana PanăNicola CavasinCristina FonteNicola Di BariCristina Galván-CasasNicola GiesenCristina GiannattasioNicola GullickCristina GluhovschiNicola HofmannCristina GrippaudoNicola IelapiCristina GugNicola LongoCristina LapucciNicola LovecchioCristina MennittiNicola MagnavitaCristina MerlaNicola MangialardiCristina MonteiroNicola MarottaCristina NadaluttiNicola MartucciCristina OlgasiNicola MaureaCristina OrtegaNicola MondanelliCristina Pindado-OrtegaNicola MontemurroCristina PredaNicola MumoliCristina SartiNicola OrzalesiCristina SecosanNicola PederneschiCristina SmolenschiNicola RosaCristina TudoranNicola SalvadoriCristina-Mihaela LăcătușuNicola SquillaceCrystal MarrugantiNicola TambascoCrystel BonnetNicola TroisiCsaba OláhNicola ValèCsongor KissNicola VeroneseCynthia E. MuñozNicola VitaleCynthia Marie-ClaireNicola WhiteCyril EleftheriouNicola ZampieriCyril FersingNicolae BacalbaşaCyril JacquotNicolae CrisanCyril SobolewskiNicolae PopCyril TouchardNicolae-Florin CofaruCyrille BlondetNicolai HuebnerCyrus DaneshvarNicolas BenechCyrus MotamedNicolas BessotCzesław MarciszNicolas BruderD. GardinerNicolas ChatainD. L. Marinus OterdoomNicolas DecerleDa SunNicolas FeltgenDagmar PavluNicolas FischerDagmar PavlůNicolas GendronDahang YuNicolas GirerdDai PuNicolas KozakowskiDaiga KishimotoNicolas MurithDaiji NagayamaNicolas PaulDaiki HoriNicolas Perez-FernandezDaiki MuraseNicolas SaroulDaiki YokokawaNicolás Sebastián RocchettiDaisaku NakataniNicolas SegalDaisuke OgiyaNicolas VerheyenDaiva BironaitéNicole AngensteinDajie ZhangNicole CamoniDali ZhengNicole NaumannDalia MedhatNicole R. Villemarette-PittmanDalila Laoudj-ChenivesseNicoleta AntonDalila ScaturroNicoletta ZermanDamian D. MarucciNicolina CapoluongoDamian HudziakNicoline De HaasDamian KaniaNicolò BrandiDamian LedzińskiNidhi AgrawalDamian PiotrowskiNidhi AnandDamiana ScuteriNidhi BhatiaDamiano PatronoNidhi SharmaDamiano PerriNidia GomezDamiano RegazzoliNidia N. GómezDamianos KokkinidisNídia Sequeira TrovãoDamien BrezulierNiels Damkjær OlesenDamien Y. P. FooNiels KokotDamir FabijanićNiels QvistDamir FranićNien Mu ChiuDamir GrebićNigel FarrowDamla TorulNiharika DixitDan Dupont HougaardNik Amin Sahid Nik LahDan HabermanNikhil AwatadeDan M. DorobantuNikhil MadanDan SongNiklas PakkasjarviDana AlbonNikol SulloDana BadauNikola KomlenacDana Gabriela FestilaNikola StefanovićDana H. BovbjergNikola TanicDana Liana StoianNikola ŽajaDana Lucia StanculeanuNikola ZmarzłyDana Maria CopolovicNikolai PaceDandan WuNikolai Pirmin JaschkeDanguolė RugytėNikolai RamadanovDania ComparciniNikolaj FrostDanial RoshandelNikolaos A. StavropoulosDaniel A. WollinNikolaos AntonakopoulosDaniel Andrei IordanNikolaos K. FragakisDaniel BarnesNikolaos KatzilakisDaniel BehmeNikolaos LaliotisDaniel BoegerNikolaos PatsinakidisDaniel BraunNikolaos SchizasDaniel C. MorrisNikolaos SiderisDaniel CandinasNikolaos SousosDaniel Demétrio Faustino-SilvaNikolaos StalikasDaniel DubinskiNikolaos TsetsosDaniel E. PorterNikolaos VrachnisDaniel EigerNikolaos Ι. VlachogiannisDaniel EyraudNikolay BugaevDaniel G. WhitneyNikolay V. GoncharovDaniel García IglesiasNikolay YanevDaniel Guimarães CacioneNikoleta PrintzaDaniel Hägi-PedersenNikos GorgoraptisDaniel J. RubinNikos MalliaropoylosDaniel Jakob NowinskiNikos MargaritelisDaniel JenkinNikos NikolettosDaniel José BarbosaNikos ViazisDaniel Josef LindeggerNilay ÇöplüDaniel L. MenkesNilesh R. VasanDaniel L. PiskorzNils LudwigDaniel LarocheNils SandmanDaniel LighezanNils WirriesDaniel LonicNilüfer Topal GöknarDaniel Lopez-MaloNimer AssyDaniel LubelskiNimi VashiDaniel MauvoisinNina Brkić-JovanovićDaniel NovakovicNina NovozhilovaDaniel NowakNina Rosa C. C. MusolinoDaniel OberladstätterNina S. YanchevaDaniel Ortega-CuellarNin-Chieh HsuDaniel OuyangNing DingDaniel P. WhitehouseNino D. HirnschallDaniel ParamythiotisNipun Lakshitha De SilvaDaniel PecsiNir BarDaniel Pedregal-MalloNir KalismanDaniel Reis WaisbergNiraj AroraDaniel Robert SchlattererNiraj VarmaDaniel Ruivo MarquesNirit Tagger-GreenDániel SandiNirmal Kumar MohakudDaniel Sanghoon ShinNiroj Kumar SahooDaniel SchmaussNisansala AbeyrathnaDaniel ŚlęzakNisha SinghDaniel TorassaNishantha NanayakkaraDaniel TozerNitika KumariDaniel TurudićNitin GuptaDaniel Vasile BalabanNiya MilevaDaniel VeghNizamettin GüzelDaniel VentusNizamuddin SheikhDaniel VirellaNobuari TakakuraDaniel WendtNobuhiko OkamotoDaniela AmitalNobuhiro TanabeDaniela AmzarNobuhiro YaoitaDaniela AschieriNobuo YoshinariDaniela BraconiNobutaka HattoriDaniela CaccamoNobuto NakanishiDaniela Carmen AbabeiNobutsune IshikawaDaniela CasatiNoel C. Y. ChanDaniela De BartoloNoel CraineDaniela Di VenereNoel G. BoyleDaniela DibelloNoel KeijsersDaniela GoncalvesNoël M. ZiebarthDaniela HartmannNoelia CasaresDaniela JunkerNokitaka SetsuDaniela L. RebolledoNolan Stuart HornerDaniela MarascoNomfundo MoroeDaniela Maria TanaseNonthapan PhasukDaniela MarzioniNoopur ThakurDaniela Opris-BelinskiNoor Al-Huda Ali A. H. SaeedDaniela ParrinoNoor AlmandilDaniela PicciottoNora SzentmaryDaniela PlatanoNorah A. FosterDaniela QuaglinoNorbert KissDaniela RadulescuNorbert TripoltDaniela RegazzoNorbert WikonkálDaniela Sonja NoschNoreen T. NazirDaniela SpanoNoriaki IwahashiDaniela SuleaNoriaki MaedaDaniela TrabattoniNoriaki SasaiDaniela ViscontiNoriaki YoshidaDaniele ArmeniaNorihiko NaritaDaniele BagattoNorihiko ShiiyaDaniele BaniNorihiro SuginoDaniele BorzelliNorihisa ShigemuraDaniele Cristina CataneoNorihisa YasudaDaniele DiacintiNoriko TakegaharaDaniele GarcovichNorimichi KoitabashiDaniele LapaNorio ImaiDaniele LinardiNorio OtaniDaniele MasaroneNorio YamamotoDaniele NovielloNoritaka OyamaDaniele RodriguezNoritoshi KobayashiDaniele SantilliNoriyuki YanagidaDaniele SolaNorma Angélica Moy-LópezDaniele TorchiaNorma JungDanieli AndradeNorma Velázquez-GuadarramaDanielius SerapinasNorman R. WilliamsDanijel SikicNorman Scott LitofskyDanijela Petković RamadžaNosotti MarioDanijela Ristić-MedićNouran Yousef SalahDanijela TasicNourhan ChaabanDanijela TrifunovicNourtan AbdeltawabDanilo BuonsensoNozawa YosukeDanilo FliserNozha RaguemaDanilo MenichelliNuman AliDanko MiloševićNuno MoraisDan-Mircea OlinicNunzia MatroneDan-Ning HuNunzia MontuoriDanny LumNunzia PastoreDanny RamzyNunzia PrencipeDante R. CulquiNunzio CirulliDanuta DykNunzio CrimiDanuta GajewskaNunzio MontelioneDanuta MakowiecNur Najmi Mohamad AnuarDanuta NowickaNuray AcarDaphna LandauNurbaity H. M. SabriDaria A. KamayevaNurettin Özgür DoǧanDaria MesselodiNúria FarréDaria TokicNúria Fernández-SabéDariga AkashevaNurul Asma AbdullahDar-In TaiNuryani NuryaniDario Antonio MeleNutjaree JeenduangDario CalafioreNuttha LumlertgulDario MessenioO. GuardamagnaDario PellegriniOana AlmasanDarius JegelevičiusOana Cella AndreiDariush IraniOana PlotogeaDariusz GąseckiOana SandulescuDariusz KałkaOana SăndulescuDariusz M. LebensztejnObianuju O. BerryDariusz Marek LebensztejnObianuju Sandra Madueke-LaveauxDariusz MoslerOctav Marius RussuDariusz SitkiewiczOctavia BaneDariusz WłodarekOctavian AndronicDariusz Wojciech MazurkiewiczOctavian EnciuDarko ChudyOded OhanaDaryl J. WileOfer MargalitDavid A. J. WilsonOfer ReiterDavid AlexanderOfir KorenDavid Alexander Christian MessererOgnjen BarcotDavid Alexander ZiringOgul Ersin UnerDavid AntcliffeOğuzhan MeteDavid Arundel HoldsworthOh-Hyun ChoDavid AstapenkoOh-Hyun LeeDavid Baeza MoyanoOhsawa SuguruDavid Bar-OrOhsuke MigitaDavid BennettOkan AalanturkDavid DonnermeyerOksana A. ShlobinDavid EdholmOktay AlginDavid EscorsOktay ÖzmanDavid Eugenio Hinojosa-GonzálezOlavo Biraghi LetaifDavid FaraoniOle Lander SvendsenDavid FerreiraOleg EpelbaumDavid FisteraOlga BrovkinaDavid FuksOlga Bruna-RabassaDavid GaucherOlga Catalina RodriguézDavid GaulOlga CiepielaDavid González-MartínOlga D. SavvidouDavid GreenhalghOlga KovalevaDavid GrynspanOlga Laporta-HoyosDavid Ilan SusterOlga LourençoDavid J. BrinkmanOlga MorozovaDavid J. HalsallOlga N. BerdinaDavid J. MathewOlga ShchaginaDavid JaffeOlga Warszawik-HendzelDavid K. BaillyOlgica NedicDavid LafonOliver BeetzDávid LíškaOliver Buchhave PedersenDavid M. AllenOliver FrickeDavid M. ManninoOliver GimmDavid M. ShererOliver GraupnerDavid MampreOliver J. OttDavid MandelbaumOliver KalpakDavid Manzano-SánchezOliver RiesenbeckDavid Michael Paul Van MeenenOliver SanderDavid MolnarOliver SommerfeldDavid MorrisOliver ThammDavid N. GreenfieldOliver VasiljDavid P. GallowayOlivia Anna MairDavid P. NaidichOlivia ZinDavid PagliaOlivier BlinDavid PakelianiOlivier DetryDavid Parada DominguezOlivier GarraudDavid PetroffOlivier Rémy-NérisDavid PoppersOlivier SchildgenDavid R. M. A. HøjgaardOlivier TraxerDavid RobergeOlle RingdénDavid S. GoldfarbOlli TenovuoDavid SacerdotiOluchi Nneka MbamaluDavid Sánchez-MolinaOluwafisayo O. AdebiyiDavid Sánchez-PorrasOm Prakash ChoudharyDavid SanterOmar A. Al-LouziDavid SchumacherOmar CauliDavid SergeevichevOmar Farookh PinjariDavid TarnokiOmar GammohDavid W. PowellOmer DzemaliDavid WestergaardÖmer KutluDavid WuOmer LinkovskiDavid ŽižekOmero Bendicto Poli-NetoDavid ZweikerOmid PajandDavide BernasconiOmkara Swami MuddinetiDavide BizzocaOmran SaifiDavide BorroniOng Ardvin Kester S.Davide CarusoOreste MarroneDavide CitterioOriana SimonettiDavide CucchiOriel SpiererDavide De CiccoOrla MorrisseyDavide Di LazzaroOrlando PariseDavide DonnerOrsola Di MartinoDavide Giuseppe RibaldoneOry WieselDavide MaggiOsama A. ElkashtyDavide MancinoOsama A. KishtaDavide Marco CrociOsama Abou-ArabDavide Maria CammisuliOsama SolimanDavide MastrorilliOsamu ImatakiDavide MedicaOsamu IshidaDavide PisaniOsamu KumanoDavide VignaleOsamu YamaguchiDavinia WithingtonOscar Cabrera-MaranteDavor PlavecOscar Hernández-HernándezDavorin SefÓscar Lado-BaleatoDawid MadejÓscar S. GallegoDawid StaudacherOskar KornasiewiczDawid StormanOskars KalejsDawid SzwedowskiOsman AktaşDawn CarnesOsnat Itzhaki Ben ZadokDaw-Yang HwangOspan A. MynbaevDayun FengOsvaldo D. MessinaDe Almeida Araújo StanleyOsvaldo L. PodhajcerDe Gaulle I. ChigbuOsvaldo MazzaDe GongOsvaldo RajmilDean Chan Pin YinOthon PapadopoulosDean D. ErdmanOurlad Alzeus TantengcoDean David Ad-ElOussama SaidiDebasish Kumar DeyOvidio De FilippDebayan DeyOvidiu Alexandru MederleDebdeep MitraOvidiu PopDebeshi DuttaÖzgül EkmekçiDebmalya SanyalOzgur KasapcopurDebora ColangeloOzgur Koray SahingozDebora CurciÖzgür SöǧütDebora Farias Batista LeiteOzren PolašekDébora FerreiraÖzüm Yuksel TunçyürekDeborah McCurdyP. G. PaulDeepa A. MalieckalP. Hemachandra ReddyDeepa KrishnaswamyP. Mangala C. S. De SilvaDeepak ChandrasekharanP. T. M. WeijenborgDeepak Kumar KhajuriaPaapa DasariDeepali P. SundraniPabitra Kumar JenaDeepika GargPablo A. LizanaDeepika JoshiPablo AvanzasDeepika SankaranPablo CaraccioloDejan BudimirovicPablo Cervera-GarviDejan GeorgievPablo Gili ManzanaroDelfa Radić-KrištoPablo OliveraDelia HorhatPablo Parra-MembrivesDelia Ioana HorhatPablo Roman-NaranjoDelia TitPablo Thomas-DupontDelminda NevesPa-Chun WangDemetrio LarraínPadet SiriyasatienDemetrio LozanoPagnoni MarioDemetrios PetropoulosPaifeng HsuDemosthenes KatritsisPaige M. HullsDenis BaranovskiiPaisarn VejchapipatDenis EvoyPäivi KannistoDenis GallotPak Hin Hinson CheungDenis J. WakehamPakcheong ChowDenisa MarginaPaloma Malagon LopezDenise GobertPaloma Ordonez-MoranDenise VeeloPamela A. MoalliDennis EurichPamela F. WeissDennis FlanaganPamela Rufus-MembereDennis GörlichPamela SenesiDennis NiebelPanagiota G. PietriDennis OrgillPanagiota KlentrouDennis P. DeVitoPanagiota MikouDenyse Thornley-BrownPanagiotis DervenisDeodato AssanelliPanagiotis GarantziotisDeokho LeePanagiotis GivissisDerek J. B. KleinveldPanagiotis I. GeorgianosDerek S. BoeldtPanagiotis KatrakazasDerek TranPanagiotis KorovessisDeren ÖzcanPanagiotis KoulouvarisDer-Shin KePanagiotis MallisDerya KarasuPanagiotis NtellasDeryk G. JonesPanagiotis PeitsidisDesh Deepak SinghPanagiotis PentarisDeshayes SamuelPanagiotis TheofilisDesiree MurianaPanagiotis TsagozisDevang J. DesaiPanagiotis TsikourasDevis BenfaremoPanayiota PetrouDewi GuellecPang-Yun ChouDeyali ChatterjeePankaj AhluwaliaDhakrit RungkitwattanakulPankaj ChauhanDhan ShresthaPankaj SinghDhanush HaspulaPantaleo GrecoDharmalingam MalaPanteleimon E. PapakonstantinouDhaval ChauhanPanteleimon GiannakopoulosDhaval PauPanteleimon PantelidisDhruba PathakPantelis KourosDi (Maria) JiangPanthier FrédéricDi WangPanu PiirainenDiana Angelika SteppanPanupong HansrivijitDiana ErnstPao-Chu ChenDiana GiannarelliPao-Huan ChenDiana JalalPaola BonavolontàDiana KwongPaola De LucaDiana MartinsPaola FacherisDiana MassalskaPaola FeracoDiana OrbeloPaola GargiuloDiana Popa-AndreiPaola L. Carvajal MonroyDiana RamašauskaitėPaola PalumboDiana Rodríguez-HurtadoPaola ParisiDiana S. WolfePaola ParlantiDiana SpiegelbergPaola QuaresimaDiana TilevikPaola RazzoreDiana ȚînțPaola SarchielliDiane M. DaubertPaolo AiròDianne StaffordPaolo Albino FerrariDíaz-Reval IrenePaolo AseniDidier A. DepireuxPaolo BanfiDidier DuclouxPaolo BennaDidier RoulinPaolo BimaDidik Setyo HeriyantoPaolo BoffanoDiego Casas-DezaPaolo BrusiniDiego DantasPaolo CameliDiego De OrtuetaPaolo CanepaDiego Fernández-VázquezPaolo CapparèDiego MoriconiPaolo CavorettoDiego RaimondoPaolo CompagnucciDiego Rodrigo MocholiPaolo DolzaniDiego VelascoPaolo FerreroDiego ZanettiPaolo FrassanitoDiesch TamaraPaolo G. ArduinoDietrich DollPaolo GrittiDiksha KatwalPaolo GroffDileep SharmaPaolo ImmovilliDilek OzcengizPaolo IovinoDiletta LoschiPaolo LimoliDillon MintoffPaolo LimoneDilyara Radikovna KaidarovaPaolo MainoDimas Alexandre KliemannPaolo MancaDimitra SkondraPaolo Maria LeoneDimitre StaykovPaolo MartellettiDimitri PoddighePaolo MenèDimitrios A. LiakopoulosPaolo MeneguzzoDimitrios DelialisPaolo MonardoDimitrios FilosPaolo PalatiniDimitrios KatsavelisPaolo PrandoniDimitrios KotsirisPaolo RighiniDimitrios PanagopoulosPaolo SalviDimitrios PapageorgiouPaolo VerdecchiaDimitrios PatouliasPaolo VersacciDimitrios StasinopoulosPaolo VincenziDimitrios TziakasPapanikolaou DimitriosDimitrios-Efthymios VlachosParaskevi TrivilouDimitris A. TsitsikasParaskevi XepapadakiDimitris DimzasParesh DoshiDimitris LambrouParham Jabbarzadeh KaboliDimitris MandalidisParimal SamirDina AbdellatifParin J. PatelDina Di GiacomoParisa GazeraniDinah ZurPariwat PhungoenDinaldo Cavalcanti OliveiraParmar AmbikaDinesh DevadossParmenion P. TsitsopoulosDinesh K. ThekkinkattilParóczai DóraDinesh KaphlePartha Narayan DeyDinko MartinovicParthasakha DasDino LuethiParveen BazardDiogo Alpuim CostaParveen RewriDiogo BrancoParvin AbediDiogo Luís MarquesParwis MassoudyDion Tik Shun LiPascal BeuretDionysios TafiadisPascal DarbonDirk Jan Van VeldhuisenPascal HilliquinDirk WalterPascal JuilleratDivanshu ShuklaPascal ProbstDivya SinhaPascal StammetDjordje G. JakovljevicPaschalis-Thomas DouliasDmitri ShchekochikhinPasha NormahaniDmitrii S. MaltsevPaško KonjevodaDmitriy Nikolaevich AndreevPasquale AmbrosinoDmitriy VidermanPasquale CaldarolaDmitry BordinPasquale CapaccioDmitry D. ZhdanovPasquale CianciDmitry GarbuzenkoPasquale EspositoDmitry TretiakowPasquale GalloDmtry RomanovPasquale LoiudiceDoddy Moesbadianto SoebadiPasquale MoneDoerte WichmannPasquale NotarstefanoDoga KuruogluPasquale PaolissoDoha ElsayedPasquale PiombinoDohern KymPasquale ViggianoDoina A. TodeaPasqualino SirignanoDolores D. GuestPasuk M. MahakkanukrauhDolphus R. DawsonPatel PalakDomagoj GlavinaPathak Chandramani M.Domagoj IvastinovicPatorn PiromchaiDomen VozelPatrice DesmeulesDomenica MangieriPatrice GaurivaudDomenico AielloPatricia DualibDomenico BeneventoPatricia Fogarty MackDomenico CiavarellaPatricia GerbargDomenico CucinottaPatricia K. NguyenDomenico CudaPatricia L. PurcellDomenico De BerardisPatricia MelinDomenico FraccalvieriPatricia Perez-CarpenaDomenico MilardiPatricia Pérez-RodrígueDomenico PiccoloPatricia Rubio-MarinDomenico RendinaPatrick A. BallDominic M. MurphyPatrick BadertscherDominik A. JakobPatrick DunnDominik BuckertPatrick G. BradyDominik DłuskiPatrick H. DesseinDominik PruskiPatrick HarnarayanDominik S. SkibaPatrick HindletDominik SaulPatrick HornDominik StodulskiPatrick KleinDominik SzymskiPatrick KrummDominique LevêquePatrick M. KochanekDominique VodovarPatrick MaréchalDomizia VecchioPatrick McCormickDomiziano TarantinoPatrick OdonkorDonald B. SandersPatrick OnyangoDonald MattisonPatrick PalmieriDonald MolonyPatrick PflügerDonatella CiarapicaPatrick SchlegelDonato ColantuonoPatrick W. BryantDonato D’AgostinoPatrik ŠimjákDong Hoon LeePatrizia CaprariDong Jin RyuPatrizia DorangricchiaDong Keon YonPatrizia LaurentiDong ZhaoPatrizia PresbiteroDong ZhouPatrizia ZaramellaDongdong RenPatrizio PetroneDong-Guk PaengPatrycja KleczkowskaDong-Hyun KimPatrycja S. MatusikDonglei SunPatryk LipinskiDongmei LiPattama WongsirisinDongrong XuPaul A. CorrisDongxin WangPaul A. KeownDongxu ChenPaul A. SobotkaDongzhu MaPaul Alfred GrütznerDonna ZwasPaul B. HicksDonovan McGrowderPaul B. KaplowitzDoreen RichardtPaul ComfortDoreen SchmidlPaul ConnellyDorin GheorghePaul D. L. P. M. Van-Der-ValkDorin IonescuPaul DalhaimerDorina LauritanoPaul DonahueDorin-Nicolae GheorghePaul EllisDoris J. DayPaul FuchsDorit Esther ZilbermanPaul GuedeneyDorit NitzanPaul HabertDorota DiakowskaPaul I. HeidekruegerDorota H. Szczesna-IskanderPaul J. GrippoDorota KaminskaPaul J. HooverDorota KmiecPaul Jacob Bueno De MesquitaDorota KopciuchPaul L. CrispenDorota LewczukPaul MuhleDorota NowosieleckaPaul OakleyDorota Pojda-WilczekPaul P. ShaoDorota Polak-JonkiszPaul PirteaDorota Tichaczek-GoskaPaul R. HarrisDorota WojniczPaul SchoenhagenDorota Zarębska-MichalukPaul SpearmanDorota ZyśkoPaul TucanDorothea TaekouraPaul Van Der ValkDorothee AlfermannPaul WillemsDorothée Kasteleijn-Nolst TrenitéPaul ZajicDorottya FrankPaula Fernández-PiresDoug VanderLaanPaula KeschenauDouglas CarlsonPaula Parás-BravoDouglas E. NeyPaula PeixeDouglas J. GriderPaula Simplício Da SilvaDouglas P. MarxPaula TavaresDouglas TaupinPaula VirkkulaDownie LilianPaulina AdamskaDo-Youn LeePaulina CeglaDragan DjordjevicPaulina KrasnodębskaDragan MaticPaulina KrawiecDragan NikolicPaulina MertowskaDragan SimićPaulina Niedźwiedzka-RystwejDragana D. Vasić AnićijevićPaulina WlasiukDragana GabrićPaulino A. AlvarezDragana LazarevićPaul-Mihai BoarescuDragana M. StanojevicPaulo Alexandre S. Armada Da SilvaDragana RadovanovićPaulo AlvesDragana RobajacPaulo CoelhoDragana Vasic AnicijevicPaulo J. PalmaDrago JelovacPaulo Magno Martins DouradoDragos AlbuPaulo Miguel PereiraDragos Catalin JianuPaulo Rosário Carvalho SeabraDragos CozmaPavan Kumar DhanyamrajuDrake Keri A.Pavel LoskotDrashya SharmaPavel RozsivalDraško PavlovićPavel ŽáčekDrew NassalPavle BanovićDror S. ShouvalPawan Kumar RaghavDrozdstoy StoyanovPaweł ChmuraDu Geon MoonPaweł GaćDuarte MarquesPaweł GutDubravka Negovetić-VranićPawel KleczynskiDubravko HabekPaweł KordowitzkiDuc M. LePaweł KrawczykDuc Trong QuachPaweł KrukowDumitru CiolacPaweł KrzesińskiDuncan ToplissPawel MiotlaDunja DegmečićPaweł NadziakiewiczDunja Maria Baston-BüstPaweł R. LataczDuo ZhangPawel ReichertDupoiron DenisPaweł RynioDuraiyah ThangathuraiPaweł SadłeckiDušan DimićPaweł WałekDušan KlosPaweł WańkowiczDusanka ObradovicPaweł WittDushad RamPaweł ZalewskiDusica PahorPayal ShahDušica PahorPayam BehzadiDustin HillersonPayam DibajDustin R. BunchPayam SadeghiE. ColonnelloPayaningal R. SomanathE. Joanne T. VerweijPayel ChatterjeeE. Mark WilliamsPearl ChenE. Reichmann-WarmuszPedro ArmarioE. SucupiraPedro BrugadaE. Sylvester ViziPedro GuimarãesEbbe EldrupPedro IglesiasEberhard UhlPedro Manuel Rodríguez-MuñozEbru Çalik KütükçüPedro MeloEbru CanakciPedro SerralheiroEbtesam Al-SuhaimiPedro ZapaterEddy KruegerPeggy B. NelsonEddy Wai Yeung WongPeichao ChenEdel AndreasPei-Chen LiEdel Noriega-ÁlvarezPei-Gen RenEdgar CambazaPeilin ZhangEdgar DalePei-Rung YangEdilmar AlvaradoPei-Wei ShuengEdison JahajPeiyi WuEdit HermeszPei-Yuan SuEdit XhajankaPekka PorelaEdita FinaPenelope MavromaraEdita FinoPeng ChenEdlira SkramiPeng GuoEdmund ArthurPeng LiEdna L. S. De SouzaPeng ZhangEdna NavaPengxiang ZhuEdna YamasakiPengyang LiEdoardo BianchiniPenny A. McKelvieEdoardo GaudePenny AsbellEdoardo Maria MuttilloPer Olof LundgrenEdoardo MazzucchiPer TornvallEdoardo MercadantePer VengeEdoardo PappaianniPerez-Valle ArantzaEdoardo PasquiPernille Lindsø AndersenEdoardo RellaPeršec JasminkaEdoardo SavarinoPertti PatinenEdoardo SciattiPertti PirttiniemiEdoardo StaderiniPessia Friedman RubinEdoardo TronconePetar OzretićEdoardo VillaniPetca Razvan-CosminEdouard CoeugnietPeter A. ChiarelliEdris A. F. MahtabPeter A. KavsakEdson Santos Ferreira-FilhoPeter A. LanzerEduard GuaschPeter AhnertEduardo A. VegaPeter AlkenEduardo Alvarez DuartePeter BališEduardo BossonePeter BaptistaEduardo Collantes EstévezPéter CsécseiEduardo DominguezPeter EickholzEduardo Ferro Dos SantosPeter F. Dutey-MagniEduardo Flores-UmanzorPeter G. PassiasEduardo García-ReyPeter H. J. Van Der VoortEduardo Hernández-PadillaPeter HlivakEduardo José Veras LourençoPeter IgazEduardo Leite CostaPeter IssingEduardo Pandolfi PassosPeter J. ApelEduardo Perez-CamposPeter JanssensEduardo Rodríguez-NoriegaPeter KeoghEduardo Yugo SuzukiPeter KorstenEdvard EhlerPéter L. KanizsaiEdvard GalićPeter LaufferEdward C. WrightPeter Lommer KristensenEdward James Frazer DansonPeter M. AndersonEdward Joel SeptimusPeter M. ProdingerEdward JohnstonePeter MichielsenEdward KijakPéter OláhEdward KoifmanPeter OttEdward R. NewtonPeter P. UjmaEdward ZimbudziPeter Physick-SheardEdwige VoissetPeter Ping LinEdwin S. MereshPeter RadselEdwin SeetPeter RammelsbergEdy IppolitoPeter RehderEdyta DziadkowiakPeter RumneyEdyta KinelPeter S. TretiakovEdyta MakuchPeter SabakaEdyta Maria UrbanskaPeter Saliba-GustafssonEdyta ZbrochPeter SchubertEelco De BreePeter SteygerEesha SharmaPéter TajtiEeva SaloPeter TschannEffrosyni KoutsourakiPeter UrbanellisEfraim WinocurPeter Van Der VoortEfrén Murillo-ZamoraPeter WahlEfstratios-Stylianos PyrgelisPeter ZhukovskyEfthichia StiakakiPether JildenstalEgbert J. D. VeenPetr DobsakEhab EssaPetr SedláčekEhsan DowlatiPetr StouracEiichi AkiyamaPetr V. KrivorotkoEiji KosePetra A. ThürmannEiji TanakaPetra AntonováEileen G. FowlerPetra HeidenEileen M. WankePetra TurčićEinas YousefPetras PrakasEinat YehenePetrisor Aurelia GeavleteEirik SøftelandPetros ChristopoulosEirini ChristakiPetros GiovanisEirini GrapsaPetros IoannouEirini Kanella PanagiotopoulouPetros IsmailidisEirini KostopoulouPetros MouratidisEirini OrovouPetros MourouzisEirini ZacharopoulouPetter HolmlundEisa TahmasbpourPetteri HoviEitan LivnyPetya ChaveevaEitan PrismanPey Yee LeeEitaro KodaniPeyman RezaieEi-Wen (Victor) LoPhatthranit PhattharapornjaroenEjemai EboreimePhern-Chern TorEkaterina FinkinaPhilip BielefeldEkaterina N. TolmachevaPhilip DueppersEkaterina SorkinaPhilip E. G. AylwardEkin SavkPhilip Georg FerstlEkram AliasPhilip Hei LiEkrem ÜnalPhilip HelliwellEla PlowPhilip HouckElaine KwongPhilip L. MarElaine Yee Lin ChungPhilip LiElanagan NagarajanPhilip McClureEl-Anwar Mohammad WaheedPhilip N. MurphyElba LlopPhilip PallmannElda DzilicPhilip SarajlicEldon MahPhilip V. GardinerEleanor KanePhilip W. TiptonEleanor SchwarzPhilip-Christian NolteEleanor ValenziPhilipp JudEleftherios AnastasopoulosPhilipp KriechlingEleftherios G. KaklamanosPhilipp M. RoessnerEleftherios GkioulekasPhilipp SewerinElena A. KorolevaPhilippe BielefeldElena Adelina TomaPhilippe CollotteElena BertelliPhilippe E. DuboisElena BlokhinaPhilippe MichelElena BollettaPhilippe O. A. Van TrappenElena CampelloPhilippe R. KoninckxElena CaporaliPhilippe SoyerElena CavalliniPhilippe ThuillierElena Cecilia RoşcaPhilippos KlonizakisElena CojocaruPhillip A. LowElena De CristofaroPhil-Robin TepasseElena De-La-Barrera-ArandaPhimon AtsawasuwanElena DobricaPhitchayapak WintachaiElena FicoPhu HoangElena G. BignamiPia GattingerElena I. BaranovaPier Francesco FerrucciElena KosenkoPier Francesco IndelliElena MarchioriPier Luigi PalmaElena PopugaevaPier Luigi TemporelliElena Porumb-AndresePier Paolo PiccalugaElena RogantePier Sergio SabaElena RolandiPiergiorgio BolascoElena RossiPiergiorgio DanelliElena SandovalPiergiusto VitulliElena ShashovaPierluigi ZoccolottiElena StoccoPiero Antonio ZeccaElena TarcaPiero FregattiElena TchetinaPiero PaladiniElena TombaPiero PiattiElena ZaklyazminskayaPiero Vincenzo LippolisElena ZelepugaPierpaolo AlongiElena ZerkalenkovaPierre AbrahamElena-Laura AntohiPierre De TruchisElena-Mihaela CordeanuPierre HuetteEleni BakolaPierre PasquierEleni GavriilakiPierre RocheteauEleni GeorgakopoulouPierre SiéEleni IschakiPierre SimeoneEleni RebelosPierre-Antoine JugeEleonora FaccioliPierre-Aurélien BeuriatEleonora GaetaniPierre-Jean SouquetEleonora GalosiPierre-Simon JoukEleonora OrtuPierre-Yves Collart-DutilleulEleonora PintoPieter MeynsEleonora TopinoPietro AusielloEleonora VatamanPietro CastellinoEleuterio A. Sánchez-RomeroPietro FeltriEleuterio SánchezPietro FusaroliElham AfghaniPietro ManiscalcoElham AhmadianPietro MaranoElham FaghihzadehPietro MazzeoEli D. EhrenpreisPietro MesircaEli MansourPietro ParcesepeEliana CostanzoPietro PellegrinoEliano CascardiPietro RosettaElias A. KouroumalisPietro ScimemiElias A. SaidPietro ValsecchiElias DiarbakerliPiia LavikainenElias FlockerziPijus BarmanElias KehagiasPilar Aparicio-MartinezElias M. EliasPilar Blanco-LoboElias ManjarrezPilar CañadasElie El HelouPilar De Lucas-RamosElie H. MotulskyPilar Pérez-RosElie KantorPil-Sung YangEliete BouskelaPınar BormanElif ŞenPinar KorkmazElin ChorellPinchas HalperinElina TrendafilovaPinelopi ArvanitiEline Borger RognliPing C. MamiyaEline H. PloumenPing SuElisa AgostinettoPingal DesaiElisa BannonePing-Tao TsengElisa BaratellaPing-Ting LinElisa BelluzziPinkal D. PatelElisa BettellaPin-Kuei FuElisa BogossianPinuccia FavianaElisa De LorenzisPiotr B. HeczkoElisa L. Hill-YardinPiotr BialasiewiczElisa María Garrido-ArdilaPiotr CzarneckiElisa MontanaroPiotr DomagalaElisa ReitanoPiotr EderElisa SchaefferPiotr F. CzempikElisa SconfienzaPiotr FutymaElisa Stellaria GrassiPiotr GabryelElisabeta AntonescuPiotr GawdaElisabeta BădilăPiotr GronekElisabeth A. KapposPiotr Henryk SkarzynskiElisabeth BendstrupPiotr JanikElisabeth HofmannPiotr JankowskiElisabeth J. M. Nieveen Van DijkumPiotr KanclerzElisabeth Nieveen Van DijkumPiotr KorczynskiElisabeth Oppliger LeibundgutPiotr KuczeraElisabeth PinartPiotr LeszczyńskiElisabetta AntonelliPiotr MalaraElisabetta BertelliniPiotr Marian HoffmanElisabetta FerraraPiotr Mirosław MaŁkowskiElisabetta GenovesePiotr MorasiewiczElisabetta GrilloPiotr MyrchaElisabetta SchiavelloPiotr PalczewskiElisabetta SetolaPiotr RolaElisabetta TosoPiotr SobolewskiElisabetta XuePiotr ToustyElisavet GrouziPiotr WilczekElise HéonPiotr WychowańskiEliška SovováPiotr YablonskiiElitsa DeliverskaPiotr ZapalaEliya LevingerPiya TemviriyanukulEliza RussuPiyush BaindaraEliza WasilewskaPiyush RanjanElizabeth AbyPlamen PanayotovElizabeth AlgarPlanellas PereElizabeth BallPo Gee Genevieve FungElizabeth BowdridgePo-Cheng ChangElizabeth C. PasipanodyaPo-Hsien LiElizabeth CondliffePo-Hsuan LuElizabeth FernandesPo-Jen HsiaoElizabeth G. IngulliPolona ZigonElizabeth I. PierpontPolyxeni MantzouratouElizabeth J. PricePompilio FagianoElizabeth MoorePonlapat RojnuckarinElizabeth Natal De-GaspariPonnulakshmi RajagopalElizabeth PekasPooja GangwaniElizaveta M. OrlovaPooja MandkeEllen BrodinPoonam KumariEllen FunkhouserPornchai SathirapanyaEllen HillegassPo-Ting WuEllen KemlerPouya HassandarvishElmina Mammadova-BachPovilas IgnataviciusElmira JalilianPrachi UmbarkarElodie BousquetPraddep Kumar BollaEloi Franco-TrepatPradeep K. SahotaEloisa Guerrero BaronaPradipta BhaktaÉloïse GiabicaniPrado Salamanca-BautistaElpiniki TsolakiPradoldej SompolEls VerhoeyenPrahlad HoElsa C. ChanPrakash Chand NegiÉlvio Rúbio Quintal GouveiaPranabananda DuttaElvira D’AndreaPranas ŠerpytisElvira MaranesiPrasanna Kumar SanthekadurElvira Orduna-HospitalPrasanna RamaniElvira SbragiaPrasanth BalasubramanianElza Ibrahim AuerkariPrashanth PrabhuElżbieta KozłowskaPrateek BhatiaElżbieta MillerPrateek LohiaElżbieta Pac-KożuchowskaPrathip PhantumvanitElżbieta PaszekPratik AgrawalElżbieta PoniewierkaPraval KhanalElżbieta RydzPravati PalElżbieta WłodarczykPraveen Gopalakrishna PaiElzbieta Zdankiewicz-ŚcigalaPraveen Kerala VarmaEmad IbrahimPravin Motilal RathiEman MehannaPravir KumarEmanuel Hernández NúñezPrawin KumarEmanuel Loeza AlcocerPreeti KishoreEmanuela D’AngeloPrem SagarEmanuela EspositoPremashis KarEmanuele BarabinoPrescott WoodruffEmanuele BernardinelliPrimož KotnikEmanuele BertagliaPrince Kwaku AkowuahEmanuele CassioliPriscila Nasser De Carvalho KrollEmanuele D’AmicoPriscilla GuglielmoEmanuele La CortePrithviraj BoseEmanuele MarzettiPriti TewariEmanuele MondaPriyakumari ThankamonyEmanuele PignattiPriyanka LahiriEmanuele PivettaPriyanka SrivastavaEmanuele QuartoPriyankar PalEmanuele RezoagliProsanto Kumar ChowdhuryEmanuele ScalaPryscilla Fanini WowkEmanuele ZaffutoPrzemysław KrakowskiEmerson S. BernardesPrzemysław TomasikEmerson Y. ChenPrzemyslaw WolakEmese MezõsiPrzemysław ŻuratyńskiEmiel F. M. WoutersPrzemysława Jarosz-ChobotEmil Marian Arbănași ArbănașiPugazhendhi SrinivasanEmile J. M. RijckenPugazhenthan ThangarajuEmilia BiffiPuhan HeEmiliano PetrucciPune Nina PaquéEmilie FréallePuneet BatraEmilien ChabrillacPuneet SaxenaEmilien JeannotPuo-Hsien LeEmilio Garcia CabreraPuppo FrancescoEmilio González-ArnayPurushoth EthirajEmilio PiccionePurva V. SharmaEmilio RamosPushpa JoshiEmilio SaccoPustika A. WahidiyatEmily BaileyPutra SantosoEmily BryanQais RadaidehEmily CaponeQalab AbbasEmily H. ChangQamar AbbasEmily HumeQasim AlhadidiEmily JohnstonQi JinEmily K. RobertsQi ShaoEmily Kay-RivestQian XuEmma Anran RanQiang LiuEmma C. JohnsonQiang XiaEmma D’IppolitoQiang XieEmma K. StappQiang ZhuEmma L. DuncanQiantao ZhengEmma MortonQiao ShiEmma MosleyQichang MeiEmma S. DuignanQijing YuEmmah DoigQimin HaiEmmanouil AlevrakisQin FuEmmanouil KapetanakisQin WuEmmanouil N. MagiorkinisQin XuEmmanouil NikolousisQing WangEmmanouil RizosQinglan DingEmmanouil TsamisQingsong TaoEmmanouil V. DermitzakisQiong GuoEmmanuel AntonarakisQiuju ChenEmmanuel CharbonneyQiuping LiEmmanuel CrouzetQiuwang ZhangEmmanuel De MaistreQuanying BaoEmmanuel GabrielQuinton S. KatlerEmmanuel J. FavaloroQuirina Santos-CostaEmmanuel KatsanisR. Douglas WilsonEmmanuel KontomanolisR. EliaEmmanuel LedoultR. KocyłowskiEmmanuel Navarro-FloresR. M. H. RoumenEmmanuel RineauR. P. H. BokkersEmre BilginR. Spiegel JosephEmre GazyakanR. Taylor RipleyEmrush RexhajRabia RiazEnass A. Abdel-HameedRabiu MusaEncarnación Martínez-GarcíaRachael E. CliffordEndre NemethRachael HachadorianEndre ZimaRachel CooneyEndrin KoniRachel E. ReillyEndrit ShahiniRachel HaweEnes AkyuzRachel KalmannEng Tat AngRachel M. RanneyEngy ElekhnawyRachel MitchellEnikő BarabásRachel MsetfiEnikő KovácsRachel O. Bluebond-LangnerEnric Alvarez-LacalleRachel S. WallworkEnric I. CanelaRachid JennaneEnric ReverterRada KazakovaEnrico AmmiratiRade M. BabićEnrico CarminaRadhika GangarajuEnrico CollantoniRadhika S. RaghunathanEnrico GallazziRadjiv GoulabchandEnrico GiordanRadka KočkováEnrico La PergolaRadmila KaranEnrico Maria TrecarichiRadmila SparicEnrico PiccirilloRadoslaw B. MaksymEnrique Alberto Guzmán-GutiérrezRadosław GocołEnrique Berjano-ZanónRadoslaw JaworskiEnrique BoldoRadoslaw Marek KiedrowiczEnrique CarreroRadu BotezatuEnrique España GrégoriRadu ChiceaEnrique Esteve-ValverdeRadu MafteiEnrique Gomez GomezRadu OlariuEnrique González-VergaraRadu Razvan ScurtuEnrique Méndez BolainaRadu VladareanuEnrique Morales-VillegasRadu-Stefan MiftodeEnrique NoreroRaelene CowieEnrique R. SorianoRafael A. GarduñoEnrique Sáez-AlvarezRafael Brandao VarellaEnrique Urrea-MendozaRafael Camacho-CarranzaEnwu LiuRafael De CiccoEnzo BerardescaRafael De La Garza RamosEnzo IerardiRafael EscateEnzo Maria VingoloRafael García-CarreteroEnzo TagliazucchiRafael IribarrenEon-Jeong NamRafael Llombart-BlancoEppie YiuRafael Moreno-Gómez-ToledanoEraldo OcchettaRafael Salido-VallejoEran BornsteinRafael Vidal-PerezEray AtalayRafał Białynicki-BirulaErbil KaramanRafal DankowskiErdem GökerRafał GerymskiErden AtillaRafał JanuszekErdoǧan SökmenRafal KocylowskiEréndira Georgina Estrada-VillaseñorRafał MoszyńskiErhan KavakbasiRafal NowakEri IshikawaRafał ObuchowiczEric A. DuboisRafał StaszkiewiczEric BlommeRaffaele AspideEric C. CheonRaffaele De CaroEric ChristensonRaffaele DubbiosoEric F. GrabowskiRaffaele GiordanoEric F. TheeRaffaele LiuzziEric GouletRaffaele SerraEric GranholmRaghunadharao DigumartiEric LeviRagnhild Munthe-KaasÉric MariotteRahim UllahEric MartinRahul D. JawarkarEric MauryRahul GuptaEric ParentRahul JainEric RoelandRahul KanumuriEric RytkinRahul MainraEric S. HagerRahul MukherjeeEric Van BredaRahul P. SharmaEric Van GorpRahul SuryawanshiEric Yi-Hsiu HuangRahul Y. MahidaErica BazzanRaili MüllerErica NeriRaimundas LuneviciusErica VetranoRaina RamnathErich CosmiRainer DziewasErich HeckerRaja ChakrabortyErick Martínez HerreraRaja DandamudiErik EliasRajagopal MuruganErik F. HauckRajan SinghErik Martin Gustaf OlssonRajat Emanuel SinghErik Roman-PognuzRajeev KumarErik SuRajesh DevassyErika Aparecida SilveiraRajesh KaluriErika Bevilaqua RangelRajesh KasamErika BonacciRajesh Kumar JhaErika MandaràRajikha RajaErika MorsiaRajinder DawraÉrika Said Abu EgalRajiv SinglaErin E. YoungRajkumar Kottayasamy SeenivasagamErin F. HoehnRajmohan DharmarajErin S. LeBlancRajni VermaErion XhepaRajya L. GurungErkan TopkanRakesh NagarajuErkin MirrakhimovRakeshkumar B. PatelErmelindo Carreira LealRalf BlendowskeErmengol VallèsRalf EberhardtErnestina SantosRalf ErberErnesto Muñoz-MahamudRalf KetterErnesto Santiago González MesaRalf LudwigErnst OberlechnerRaluca GrigoreEros MontinRaluca IsacErsin GümüşRaluca LupușoruErtan AkbayRaluca Simona CostacheErtuğrul ŞahinRaluca TomoaiaErwan DumontetRam PrasadEsma ÖzkanRam RamamurthiEsperanza MerinoRam SagarEsra PolatRam SamujhEssam Al-MoraissiRamachandra BarikEsteban Agulló-TomásRamachandran RajalakshmiEsteban Obrero-GaitánRamachandran VinithaEsteban Puente-LópezRamalingam BethunaickanEstefania NuñezRaman MehrzadEstelle SeymanRamana VakaEsther SanfeliuRamanuj LahiriEsther ScheirlynckRamendra Pati PandeyEstíbaliz JiménezRamesh BabuEstrella RausellRamesh MukkamalaEszter DuczaRami N. Al-RohilEthan David CohenRami S. KantarEtienne Cho Ee WangRami S. NajjarÉtienne J. BissonRamit Maoz-SegalEtienne MarbaixRamnik V. PatelEtsuko MiyagiRamón Ruíz-MesaEttore CaroppoRamon TornéEttore Di TrapaniRamona Gabriela UrsuEttore MeariniRamona MeantiEttore SilvagniRamona SchweyenEugen TãrcoveanuRamy AbdelnabyEugène A. A. RameckersRamy Abou GhaydaEugene HanRan AtzmonEugene Tze-Chun LauRan DuEugene Y. H. YeungRan HarelEugenia AllegraRan KornowskiEugenia Irene DavidescuRan XuEugenio BertelliRana El RawasEugenio CammisaRanadheer R. DandeEugenio MartelliRandall T. LoderEugenio RagazziRandi J. ReidunsdatterEun Ho ChooRandi VitaEun Hwa ChoiRandolph H. L. WongEun Jeong HwangRandy M. CohnEun Ji LeeRandy NeblettEun Young ParkRangi Kandane-RathnayakeEung-Kwon PaeRanjan PremaratnaEun-Jung LeeRanjeev Chrysanth PulleEun-Young ParkRanjini SankaranarayananEusebi ChinerRanjit Kumar PaulEustaquio AbayRanjiv MathewsEuthymia VlachakiRanju Kharel SitaulaEva BaranovičováRanko M. KutlešićEva BroströmRao SunEva Ekvall HanssonRaoul BergnerEva HasslerRaoul BreitkreutzEva HavrdovaRaphael CiumanEva KnuplezRaphael FavoryEva Maria MatzholdRaphael Jay WitorschEva Martinez-JimenezRaphaël LeComteEva Mihalyko-OrbanRaphael ScherbaumEva Pereda-PeredaRaphael SchiffmannEva Piano MortariRaquel Aparecida CasarottoEvan LedermanRaquel AscençãoEvan LutherRaquel Benedé UbietoEvan RossRaquel Bouça-MachadoEvangelia AkoumianakiRaquel CorripioEvangelia AntoniouRaquel CostaEvangelia ChavdoulaRaquel EchavarriaEvangelia KouidiRaquel Esteves MarquesEvangelia KoukakiRaquel Fernandez-GarciaEvangeline MantziorisRaquel Leirós-RodríguezEvangelos MaziotisRaquel LeonEvangelos SymeonidisRaquel Mejias LuquEvann E. HiltRares CraciunEvanthia BernitsasRareş Mircea BîrluţiuEvanthia BletsaRasa BarkauskienėEvasio PasiniRasa MladenovicEvdoxia GogouRasa TamelienėEve NamisangoRasa ZarnegarEvelina Maria GosavRashika E. I. RidiEveline L. A. Van DorpRasmus Hvidbjerg GantzelEvert F. S. Van VelsenRasmus RiviniusEvgeni BrotfainRasoul MirzaeiEvgeny BlagovechtchenskiRatchadawan AukkanimartEvgeny D. KasyanovRatheesh Kumar MeleppatEvi FleischhackerRatko LasicaEwa Boniewska-BernackaRatko PrstačićEwa GrudzińskaRatna Sudha MadempudiEwa Jȩdrzejczyk-PatejRatnadeep MukherjeeEwa Otto-BuczkowskaRaúl Juan Molines-BarrosoEwa PiotrowskaRaúl López-IzquierdoEwa SkarżyńskaRaúl Quevedo-BlascoEwa TomaszewskaRaul SebastianEwa W. A. OlszewskaRaúl Soto-CámaraEwa WieloszRavi A. ThakkerEwa Wiesik-SzewczykRavi Kumar SharmaEwald LindnerRavi ManoharanEwelina Elert-DobkowskaRavi Philip RajkumarEwelina MłynarskaRavi Shankar ReddyEwelina WardzinskiRavindra A. PrabhuEyal LebelRavindra DeshpandeEytan MorRay MannehEzequiel José ZaidelRay Vern MatthewsEzio LanzaRay W. SquiresFabia AttiliRaymond H. KimFabian BartschRaymond J. WallsFabian J. BrunnerRăzvan Adrian IonescuFabian Martin TroschelRăzvan George RahotăFabian MedvedRǎzvan HainǎroşieFabian QueissertRazvan SocolovFabian StubyRăzvan-Ionuț PopescuFabian TomschiRebeca Muñoz-RodríguezFabian WeykampRebecca J. StoresFabiana GregucciRebecca K. MartinFabiana LucàRebecca LundinFabiana PaniRebecca NantandaFabien PicardRebecca S. EshraghiFabien SauvetRebecca T. HahnFabio CarboneRecep DursunFabio García-GarcíaRed M. AlinsodFabio GuoloRedha TaiarFabio MaglittoRedi RahmaniFabio Massimo UlivieriReena VsFabio MelandroRegie Santos-CortezFabio MendesRegina Ferreira AlvesFabio MiraldiRegina Fölster-HolstFabio Nishimura KanadaniRegina PodlasinFabio PellegrinoRegina Tangerino Souza JacobFabio PerrottaRegina WierzejskaFábio Roberto CabarReham HamidaFabio SantacaterinaReidar P. LystadFabio TimeusReiki NishimuraFabio TrastulliReiko AritaFabiola GiudiciReimer RiessenFabiola SpolaorReina Bianca TanFabrizia D’ApuzzoReinhard KoppFabrizio BambiniRéjean CoutureFabrizio Di MaidaRéka Eszter SzivaFabrizio GozziRekha KhandiaFabrizio Maria GobbaRémi CoudroyFabrizio MartoraRémi GrangeFabrizio MontecuccoRemigiusz BąchorFadar Oliver OtiteRemigiusz KazimierczykFady ShararaRemington L. NevinFahad A. AlhumaydhiRemo H. M. FurtadoFahad KhanRen JianjunFahad UmerRenata BalnytėFahiel CasillasRenata C. N. MarchetteFairuz Azman KhairunnuurRenata C. ScalcoFaisal F. HakeemRenata FinelliFaizah Safina BakrinRenata Pacholczak-MadejFang LiRenata Simona AuriemmaFang LiuRenata Świa̧tkowska-StodulskaFang XuRenata Voltolini VelhoFanny EysertRenata VrsalovicFanny TréchotRenate KainFanpei Gloria YangRenate KrassnigFantahun DegneheRenato CozziFa-Po ChungRenato J. VerdugoFaraedon ZardawiRenato Patrizio CostaFardod O’KellyRenato PietrolettiFares E. M. AliRenato TorresFarhad VahidRenaud De TayracFarideh RaziRene CorteseFaridoddin ShariatyRené M. CasteleinFaris F. BrkicRené M. RothweilerFarnoosh SeirafianpourRené WubbelsFarr Reza NezhatRengin GüzelFaruk BuyruReni KalfinFarzad Taghizadeh-HesaryRen-In YouFarzan SolimaniRenjie HeFateen AtaRenke MaasFatemeh AshrafianRenu VirmaniFatemeh KhatamiRenu WickremasingeFatemeh MansouriReparata Rosa Di PrinzioFatih AdaReshma R. GolamariFatih DurmuşoǧluRevathy CarnagarinFatima MartinsRex N. SmithFatimah Saeed AlhamlanReza AlizadehFatime HawcharReza JafariFatma InaniciReza PiriFatma YıldırımReza Rafiolsadat SerizawaFatme AlAnoutiReza RanjbaranFatmir DragidellaReza ShahriariradFausto BiancariReza Shekarriz-FoumaniFausto FamàReza TabriziFausto RigoReza ZamaniFausto RovetaRezvan JamaledinFayaz Rahman KhanRiaan MulderFedele LemboRiadh BadraouiFederica BuccinoRicardo A. Cartes‐VelásquezFederica Di SpiritoRicardo CabezasFederica FogacciRicardo Castro AlvesFederica GalimbertiRicardo DiasFederica Genovesi-EbertRicardo Faria RibeiroFederica GrossoRicardo FelberbaumFederica GuerraRicardo Guilherme ViebigFederica MagagnoliRicardo J. JoseFederica MaggiRicardo Rivera-LópezFederica MesciaRicardo Ruiz-VillaverdeFederica PianiRicardo Soares-Dos-ReisFederica PortunatoRiccardo BientinesiFederica VeroneseRiccardo CampiFederico BoscariRiccardo CappatoFederico CacciapuotiRiccardo CastagnoliFederico GeraldiniRiccardo FerraciniFederico GerminiRiccardo MarmoFederico GuerraRiccardo MastroianniFederico Lo TortoRiccardo MonterubbianesiFederico Manuel GiorgiRiccardo PistelliFederico MarchesiRiccardo PozzanFederico MazzacaneRiccardo TelliniFederico MigliorelliRiccardo VioFederico MorettiRich KalletFederico PerfettoRichard BodnarFederico RoncoRichard BorderFederico TacconiRichard C. DowellFederico VancheriRichard F. RiedelFedor NovikovRichard J. StarkFei XingRichard JamesFelice CrocettoRichard John Bruce FrancisFelice Eugenio AgròRichard KdolskyFelice FemianoRichard KobzaFelicitas BucherRichard KozarekFelipe AlconchelRichard LemalFelipe Araya-QuintanillaRichard M. PinoFelipe MachadoRichard MusilFelipe ParedesRichard N. MerchantFelippe Lazar-NetoRichard VollenbergFelix ChuaRichard Yudi HidaFelix De CarlosRichie H. S. GillFelix ErneRichte C. L. SchuurmannFelix F. GirrbachRick A. FriedmanFelix GassertRiham M. FliefelFelix HagenauRihui LiFelix HüttnerRiikka MartikainenFelix InchaustiRinesh GodfreyFélix Javier Jiménez-JiménezRini Devijanti RidwanFélix Marcos TejedorRinki MinakshiFelix Marius BläsiusRintu ThomasFelix Martin WernerRishabh C. ChoudharyFelix Mathias WagnerRishi Man ChughFelix MehrhofRishi RajFelix RommelRisto KauppinenFeng-Lin YenRita Carlotta SantoroFengwei ZouRita ConigliaroFengyu ChiangRita ConsoliniFerdinando AgrestaRita LealFerenc DomokiRita MatosFerhat MezianiRita PavasiniFernanda Andrade OrsiRita PintoFernanda Barros-AragãoRita PolitoFernanda GentilRitthideach YorsaengFernanda RodriguesRitu ChauhanFernandez Alba Juan JesusRiyad C. Karmy-JonesFernando Álvarez-GuisasolaRobert A. BokFernando Augusto Ugusto Mardiros HerbellaRobert A. RamirezFernando CordidoRobert A. SchwartzFernando CorvilloRobert BłaszczykFernando Freitas GanançaRobert BonekFernando GilsanzRobert Brawura-Biskupski-SamahaFernando Guerrero-RomeroRobert BreitkopfFernando J. AbelhaRobert BreuerFernando Montenegro SáRobert C. BahlerFernando Muñoz-HernándezRobert CesnjevarFernando Padovan-NetoRobert Cristian PurdoiuFernando SantosRobert CubasFernando Suarez-SipmannRobert DaileyFernando Suparregui DiasRobert DammFerras AlashkarRobert Douglas WilsonFerreira-Gonzalez IgnacioRobert E. PykeFevzi YılmazRobert EgermanFeyza F. DarendelilerRobert F. AsarnowFeza DeymeerRobert FlisiakFeza KorkusuzRobert FrithiofFilbert TotsinganRobert GajdaFiliberto Maria SeveriRobert GuidoinFilip Franciszek KarugaRobert James CassonFilip JezekRobert Jay WidmerFilip SedlicRobert L. SatcherFilipa SousaRobert L. SchelonkaFilippo AucellaRobert LämmerFilippo ConfalonieriRobert LarribauFilippo Crimi’Robert Mackenzie RossFilippo MarianoRobert MaittaFilippo MaselliRobert MarkewitzFilippo MiglioriniRobert NaeijeFilippo MocciaroRobert NowakFilippo OliveriRobert PasławskiFilippo PelizzaroRobert Peter GaleFilippo PieralliRobert Philipp KosilekFilippo T. GallinaRobert PowersFilippo Tommaso GallinaRobert PrillFilippo TorielloRobert R. GaiserFilippo VittadiniRobert S. BenjaminFilippos KontosRobert SabiniewiczFilippos VingopoulosRobert StansburyFiliz TütüncülerRobert T. BradyFin MacraeRobert T. KuthanFinn Edler Von EybenRobert T. MeansFinn JohannsenRobert TyckoFintan MoriartyRobert WestFiona BruinsmaRoberta GelminiFiorenzo MoscatelliRoberta GrassiFiras Abu AkarRoberta ModicaFirdaus HaririRoberta MonzaniFiroz AkhterRoberta ParenteFitri OctavianaRoberto Adriano LatiniFlavia ChiosiRoberto BellucciFlavia GhellerRoberto CalistiFlavia PauriRoberto Carlos Castrejón-PérezFlavia PuglieseRoberto CasconeFlavia VentrigliaRoberto CastiglioneFlavio AimbireRoberto CirocchiFlavio De Alcantara CamejoRoberto CuttanoFlavio G. RochaRoberto De PontiFlavio HeldweinRoberto Gonzalez-SalinasFlávio HojaijRoberto H. ParadaFlávio LaraRoberto LicordariFlavio MilanaRoberto Lo GiudiceFlavio TarasoutchiRoberto MaglieFlemming AndersenRoberto Martínez ÁlvarezFlor H. PujolRoberto MenèFlora N. BalievaRoberto N. MirandaFlorence TardyRoberto Oliveira DantasFlorencio Jr. ArceRoberto PradoFlorentina PluteanuRoberto RonaFlorian BaltăRoberto ScicaliFlorian BaudinRoberto VernaFlorian EmmerichRobertus Van AalstFlorian EnzmannRobiatul AdawiyahFlorian EyerRobin Man KarmacharyaFlorian FalterRocco FrancoFlorian FrankRocío Llamas-RamosFlorian G. ScurtRocío Muñoz-HernándezFlorian HeilmannRocio Nuñez-CalongeFlorian K. EbnerRocio Palomo-CarriónFlorian LipsmeierRocio ToroFlorian N. LochRod MacLeodFlorian PiekarskiRodger J. ElbleFlorian PohligRodica BalasaFlorian ReckerRodica Maricela AnghelFlorian SchachingerRodney SinclairFlorian ZorèsRodolfo HurleFlorin MituRodolphe BaillyFlorina NedeleaRodrigo A. CornejoFlorina S. IliescuRodrigo AnguitaFloris S. Van Den BrinkRodrigo Bernardo SerafimForogh SoltaninejadRodrigo Cappato AraujoFortunato BattagliaRodrigo FrançaFortunato IacovelliRodrigo OkuboFortunato LonardoRodrigo Pinheiro AraldiFoteinos-Ioannis DimitrakopoulosRodrigo ResendeFrance WalletRodrigo Rollin-PinheiroFrances Meier-GibbonsRodrigo Romero-NavaFrancesc MoresoRodrigo Sandoval BoburgFrancesca AmbrosiRodrigo Torres-CastroFrancesca AmorosoRoel H. A. WeijerFrancesca BaessatoRoel Van OvermeireFrancesca BarileRogelio Gónzalez-GónzalezFrancesca BarrettRoger AckroydFrancesca BovisRoger AdamsFrancesca BruzzeseRoger AlvarezFrancesca BuonomoRoger BouillonFrancesca CattoniRoger E. KelleyFrancesca CecchiRoger E. ThomasFrancesca CrisafulliRoger NasciFrancesca GainoRogério Manuel FerreiraFrancesca GalluzziRogerio Serafim ParraFrancesca IettaRogier P. VoermansFrancesca LatinoRohan Bir SinghFrancesca LazzaraRohana AhmadFrancesca MonariRohit GuptaFrancesca NapolitanoRohit JainFrancesca NegroRohit MoudgilFrancesca PisanoRohit RanganathFrancesca Sangiuliano IntraRok CivljakFrancesca TessitoreRok ČivljakFrancesca VincitorioRok GaspersicFrancesco AcerbiRoksana MalakFrancesco BartolucciRola JaafarFrancesco BellinatoRoland FenkFrancesco BennardoRoland S. CamenzindFrancesco BiancoRolandas ZablockisFrancesco BonomiRolando Ulloa-GutierrezFrancesco BoscoRolf LjungFrancesco CanonicoRolf N. BarthFrancesco CardaioliRomain ChopardFrancesco CarocciaRomain GilletFrancesco CastagniniRoman HaberlFrancesco CeiRoman MichalikFrancesco ClapsRoman NowickiFrancesco ColomboRoman PfeiferFrancesco De ChiaraRoman SmolarczykFrancesco DogliettoRoman TrochimczukFrancesco FacchinettiRomina Marina SimaFrancesco Fascetti LeonRomualdas MalinauskasFrancesco FerraraRon ShapiroFrancesco FontanaRonak G. SoniFrancesco GallettiRonak ReshamwalaFrancesco GaziaRonald B. BrownFrancesco GirolamoRonald S. JonesFrancesco Giuseppe MartireRonald SrokaFrancesco ImpagnatielloRonan TanguyFrancesco InchingoloRonen JaffeFrancesco LiuzzaRongkung TsaiFrancesco MaestriRonit AbirFrancesco MaffìaRoo Min JunFrancesco MarottaRoopali DonepudiFrancesco MeaniRoopesh Singh GangwarFrancesco MelilloRosa BocciaFrancesco MeroiRosa Giovanna SimonettiFrancesco MocciaRosa KlotzFrancesco MongelliRosa Krajmalnik-BrownFrancesco NappiRosa M. MartiFrancesco OrziRosa María Cabanas ValdésFrancesco PataRosa María Cardenal-PirisFrancesco PatanèRosa Maria GerardiFrancesco PellegriniRosa María López-Pintor MuñozFrancesco PellicciaRosa Martha Meda LaraFrancesco PepeRosa Martínez-PiédrolaFrancesco PetrilloRosa Redolat IborraFrancesco PiccirilloRosa SuadesFrancesco PisanuRosalba SiracusaFrancesco PontieriRosalia DettoriFrancesco ProcaccioRosália SáFrancesco RiaRosalinda MadonnaFrancesco SabbatinoRosanna DammaccoFrancesco SartiniRosanna VillaniFrancesco Saverio LudovichettiRosaria BarracanoFrancesco Saverio SorrentinoRosário SantosFrancesco SecchiRosario SarabiaFrancesco SegalaRoser GraneroFrancesco SessaRoser VelascoFrancesco SolimeneRosetta RagusaFrancesco SpallottaRoss LeightonFrancesco SpannellaRossana IzzettiFrancesco SpinelliRossella RuggieroFrancesco StringaRossella SgarzaniFrancesco TandoiRossen Mihaylov HazarbassanovFrancesco TrainaRo-Ting LinFrancesco TramaRottem KuintFrancesco U. S. Mattace-RasoRoushu ZhangFrancesco VenturelliRowan S. HardyFrancesco VitaliRowe BertrandFrancesco ZaracaRoxana Cristina RimbaşFranchek DrobnicRoxana Maria NemeșFrancina FonsecaRoxana Silvia BumbaceaFrancina MunellRoxana VasiliuFrancisco A. Gómez-VeigaRoxanne BavarianFrancisco Abad-SantosRoy LauterbachFrancisco Alcantud MarínRoy MashiachFrancisco BuitragoRoya NavabFrancisco Carlos Pérez-MirallesRoya OstovarFrancisco CarmonaRubén Cuesta-BarriusoFrancisco Do ValeRuben Lopez-BuenoFrancisco E. MartinsRuben M. PinkhasovFrancisco E. NicolasRuben Panadero AgustinFrancisco Espinoza-GomezRuben PauwelsFrancisco FernándezRubén TriguerosFrancisco Gonzalez-SalaRubens C. SiqueiraFrancisco Guede-RojasRudolf ČernýFrancisco Guillen-GrimaRudolf F. GuthoffFrancisco Guzman PrunedaRudolf Hans TinnebergFrancisco Javier Pérez-RivasRuhul AbidFrancisco Javier Povedano-MonteroRui DingFrancisco José Gómez GarcíaRui DuanFrancisco José HidalgoRui Farinha FarinhaFrancisco José Sánchez-TorralvoRui GaoFrancisco Miguel Martínez-ArnauRui LiFrancisco Navarro-TriviñoRui Pedro OliveiraFrancisco Pérez-BartoloméRui TorresFrancisco RiveroRuida HouFrancisco Sandro Menezes-RodriguesRuifang LuFrancisco TustumiRuijie HuangFranck ChiappiniRuitu LyuFranco Alchiede SimonatoRujuan DaiFranco BassettoRu-Lan HsiehFranco FranceschiniRulin ZhuangFranco GemignaniRumen KastelovFrancois CornelisRuna KuleyFrançois DuboeufRundk HwaizFrancois EyskensRune A. KrokenFrancois FaitotRuntang MengFrançois JamarRupendra ShresthaFrançois MontagneRupert HindsFrançois PhilippartRupert W. StraussFrançois PigotRupesh Kumar ChikaraFrançois RouthierRussell F. D’SouzaFrançois SaucyRussell HodgsonFrançois SchieleRustem MustafaogluFrançois TrotteinRūta VaičiūnienėFrançoise Stanke-LabesqueRute SampaioFrancois-Pierre CounilRuth TachezyFrane PaicRuth TarziFrank Blanco-PerezRuth ZimmermannFrank HaoRuud SchreursFrank R. E. NobelsRuxandra Diana SinescuFrank Vinholt SchiødtRuxandra IonescuFranka Klatte-SchulzRuxandra-Iulia MilosFranz Felix KonenRuziana MasiranFranz Josef PutzRyaan El-AndariFranz ZehentmayrRyan D. SullivanFranziska AlbrechtRyan Gouveia E. MeloFranziska DornRyan J. StubbinsFranziska WeberRyan J. WinsteadFranz-Josef SchingaleRyan M. MarquardtFrédéric GilbertRyan SullivanFrederic LioteRyan WereskiFrédéric RocheRymantė GleiznienėFrederic TriponezRyo InuzukaFrederick H. SilverRyo MaekawaFrederiek Van Den BosRyo MomosakiFrederik DenormeRyo NakamaruFrederik RaiskupRyogo MinamimotoFrederik. BerrevoetRyoichi AsakaFredric D. GordonRyoki SasakiFredrik H. SterkyRyoko AsanoFredrik HussRyong Nam KimFredrik NystromRyota MasuzakiFriederike HolzeRyota TamuraFriederike SchömigRyszard ŻabaFriedhelm BeyersdorfRyu MatsuokaFrikkie J. W. CalitzRyu WatanabeFrits Van MerodeRyuichi OhtaFronczek JakubRyuji TakedaFu DehaoRyungsa KimFuad K. HashemS. H. ShinFu-Jen ChengS. M. Yasir ArafatFulvia ZappuloSaad AlghamdiFulvio CaliseSaad Ihsan ButtFulvio LauretaniSaagar MahidaFulvio MassaroSaba BattelinoFulvio TartaraSabarinath PrasadFumihiko KoiwaSabina Antonela AntoniuFumihiro OgawaSabina GallinaFumikazu YamazakiSabina SevcikovaG. ChenitiSabine SchmatlochG. S. PushkarevSabrina HaroonGábor Gy DeákSabrina MartinezGabor J. BartonSabrina PellicciaGábor L. KovácsSabrina SandriesserGábor LipositsSabyasachi DashGabriel AnesettiSachin GoyalGabriel CismaruSachio TakenoGabriel Claudiu TenderSachiyo YoshioGabriel Dias RodriguesSadaf JahanGabriel DjedovicSadegh Amani-ShalamzariGabriel HancuSadia AjazGabriel NallathambiSadia SarwarGabriel SosneSadia ShakeelGabriel WagnerSadiq HussainGabriel WajnbergSaeed AnwarGabriela Anaya-SaavedraSaeed Reza MotamedianGabriela AniteiSaei Ghare Naz MarziehGabriela DrocSaeid KargozarGabriela GarcíaSaffet OzturkGabriela MahelkováSafwan OmranGabriela MotoieSagar SohoniGabriela Salim Ferreira De CastroSagarika BiswasGabriela SiedlerSagrario Gomez-CantarinoGabriele CanziSahar KeshvariGabriele CervinoŞahin HanalıoğluGabriele Gallo AfflittoSai Kiran BhagraGabriele GhettiSaidur MashrekyGabriele HaeuslerSaif KhanGabriele MelegariSaima RubabGabriele PaganiSait KaramanGabriele PesariniSajjad ShiraziGabriele PesimenaSakeenabi BashaGabriele SacconeSakineh HajebrahimiGabriele SavioliSakineh VarmazyarGabriele SpoletiniSakthi RajendranGabriele TodiscoSalah Eldien AltarabshehGabriele TumminelloSalam Salloum-AsfarGabriele ZanottoSalem Youssef MohamedGabriella BottariSally Ann HuberGabriella ColicchioSally PaulsonGabriella DvorakSalma AbedelmalekGabriella VerucchiSalma TahaGabrielle D. BriggsSalman Afroze Azmi MohammedGabrielle DrevetSalman ZahidGabrielle RigneySaloua MrabetGabrielle RudolfSalvador Castaneda VegaGad SegalSalvador García-DelpechGadelkarim MohamedSalvador GonzálezGaetano Antonio LanzaSalvador MenaGaetano De RienzoSalvador Romero-ArenasGaetano FassiniSalvatore BocchieriGaetano GalloSalvatore BracchittaGaetano PaoloneSalvatore CozziGaetano PaoneSalvatore GiordanoGaetano SantulliSalvatore GrisantiGaetano ScaramuzzoSalvatore IaconoGagan FloraSalvatore Ivan CaicoGaia BarisioneSalvatore Maria CorselloGaia BonassiSalvatore MastroliaGaia PipernoSalvatore SciacchitanoGail F. ShustSalvatore SclafaniGail WhitelawSalvatore ScolavinoGairik KunduSalvatore SorrentiGajane MartirosianSam KantGal TsabanSam RazaeianGalina V. KopylovaSam Yeol ChangGallus BischofSamad EsmaeilzadehGamze BildikSaman WarnakulasuriyaGamze Gamze GokalpSamantha PascaGanesh BavikatteSamantha PayneGanesh Ram VisweswaranSamantha S. SoldanGang HuangSamantha ShanGang PengSamar TharwatGang WangSamara Tatielle Monteiro GomesGanta Vijayx ChaitanyaSamasca GabrielGarey NoritzSameer A. PatelGarrett BurnettSameh AttiaGary A. HeimanSamendra KarkhurGary C. BrownSamer SroujiGary J. FarkasSamhati MondalGary J. KennedySami Alexander SafiGary K. YangSami MyllymaaGary NaceSami QadriGary S. BeasleySamineh MesbahGaryfallia PeperaSamir A. ShahGassan MoadySamir K. BallasGaston J. PiñeiroSamira TarashiGaurav BaranwalSamo FokterGaurav GuptaSamrad MehrabiGautham YepuriSamrita DograGautier ChêneSamson G. KhachatryanGavino CasuSamuel C. C. ChiangGavino FaaSamuel Fernández CarneroGaya Punnia-MoorthySamuel FrankGayatri Shankar ChilambiSamuel J. ZolinGea OlivericontiSamuel LawmanGebhard MathisSamuel Mawuli AdadeyGeert H. GroeneveldSamuel MenahemGeert MayerSamuel SchechtmanGeeta RaoSamuela TarantinoGeetha SivasubramanianSamuele CerutiGeetu BhandoriaSamuele TarditoGelei XiaoSamuele VaccariGema Ariceta IraolaSamuli JaakkolaGemma Chiva-BlanchSamy M. RiadGemma MarcucciSamy MetyasGengo SunagawaSanaa Najeh Al-Haj AliGenis CampreciosSanam Shahla RizviGenis CardonaSanchita DasGennaro AulettaSandeep DhalluGennaro Mariano LenatoSandeep K. DhalluGennaro MartucciSandeep VijayanGentaro KumagaiSandi Baressi ŠegotaGeoff HackettSándor-György ZuhGeoffroy HaririSandra Alcaraz-ClarianaGeorg C. ZieglerSandra Anić-MiloševićGeorg F. VogelSandra BarbalhoGeorg GelbeneggerSandra D’AscenzoGeorg GihrSandra FrancoGeorg HalbeisenSandra GavinhaGeorg M. StettnerSandra GiménezGeorg SingerSandra GuidiGeorg SprinzlSandra HirschGeorg WolffSandra Jiménez-Del-BarrioGeorge A. KoumantakisSandra Kalil BussadoriGeorge A. R. WigginsSandra Milena Colorado-YoharGeorge A. TavartkiladzeSandra Santos-MartinezGeorge A. TruskeySandra Van Der AuweraGeorge Alexandru MafteiSandrine DemanècheGeorge AnifandisSandro BrusichGeorge BaylissSandro GelsominoGeorge BertsiasSandro GlumacGeorge FotakopoulosSandro MicheliniGeorge ImatakaSandro NinniGeorge J. KontochristopoulosSandro RengoGeorge KarpetasSandro SchlindweinGeorge KuoSang Ho KwakGeorge L. HinesSang Hun KimGeorge LabirisSangappa B. ChadchanGeorge M. AbrahamSangeetha ThangaswamyGeorge N. KouvelosSang-Heng KokGeorge PadosSang-Hwan DoGeorge PaltoglouSangita P. PatelGeorge PanosSang-Mok LeeGeorge PavlidisSang-Moo LimGeorge R. KasyanSang-Won LeeGeorge SamanidisSanja B. VujkovGeorge SoulisSanja DabelicGeorge TavartkiladzeSanja DespotovićGeorge TherapondosSanja HromišGeorge UmemotoSanja IvkovicGeorge VartholomatosSanja Lovric KojundzicGeorge YendewaSanjaya Kumar SahuGeorges AzarSanjid ShahriarGeorges DellatolasSanjin KovacevicGeorges MaestroniSanjiv RisalGeorges MjaessSankar BhattacharyyaGeorges TarrisSankar JagadeeshanGeorgi VassilevSan-Pin WuGeorgia GomatouSantanu RanaGeorgia KaiafaSanthosh MukundanGeorgia KarpathiouSantiago BonanadGeorgina Cárdenas-LópezSantiago Ortiz-PerezGeorgina NakaferoSantino Ottavio TomasiGeorgios Antonios MargonisSanto Raffaele MercuryGeorgios BenetosSantos CastañedaGeorgios D. PanosSantosh DhakalGeorgios GalaziosSantosh S. JeevannavarGeorgios I. KaraolanisSara C. ZapicoGeorgios IatrakisSara GagoGeorgios KokolakisSara I. AbdelsalamGeorgios LaliotisSara McCulloughGeorgios MourtzinisSara MoraisGeorgios N. AntonoglouSara MoscatelliGeorgios NarosSara NunesGeorgios NikolakisSara ParigiGeorgios SahsamanisSara RegnérGeorgios SianosSara ReisGeorgios Sokratis ChatzopoulosSara Salehi HammerstadGeorgios TagarakisSara SchrammGeorgios TzavellasSara ShimoniGeorgios TzikosSara Souto-MirandaGeorgios VrakasSara Stasik-O’BrienGeorgiy KorobeynikovSara SuccuGerald KloseSara TorrettaGéraldine PoenouSara TouhamiGeraldo A. Maranhao NetoSarah B. StringfieldGerard A. SalameSarah El-NakeepGerard AnmellaSarah GephineGerard EspinosaSarah Kittel-SchneiderGerardo CazzatoSarah LawrenceGerardo GuintoSarah LensenGerardo Santos-LópezSarah M. JurickGerasimos EvangelatosSarah MartaindaleGerd Marie AleniusSarah ThomisGerdi TuliSaran LotfollahzadehGergana PetrovaSarel HalachmiGergely ÁgostonSarfaraz IqbalGergely FeherSargis SedrakyanGergő JózsaSarhang HamaGermain HonvoSarika GuptaGermana BanconeSarina Levy-MendelovichGermina-Alina CosmaSarita LagalwarGernot FuggerSaritphat OrrapinGerry CoghlanSariya MapoungGertraud Gradl-DietschSarkar M. Abe KawsarGessynger Morais-SilvaSarra SmatiGeu Mendoza CatalánSasa RajsicGhada Hussein NaguibSasan FaridiGhasem FarahmandSascha DavidGhasem SolgiSascha HalvachizadehGheorghe-Andrei DanSashka KrumovaGheun-Ho KimSaskia H. M. Van RomundeGholam Hossein RoshaniSathish ThirunavukkarasuGholamreza DaryaborSathyadeepak RameshGhufran BabarSathyavathi ChallaSivaKanakaGi Beom KimSatoru EgawaGiacomo BaimaSatoru KaseGiacomo BertoliniSatoru KyoGiacomo BusoSatoru MiyawakiGiacomo CasaliniSatoru MunakataGiacomo Dal BelloSatoru MutoGiacomo De RiuSatoshi KatoGiacomo E. IapichinoSatoshi KishigamiGiacomo FarìSatoshi NakasuGiacomo GaribottoSatoshi NozawaGiacomo M. VianiSatoshi SeinoGiacomo MiglionicoSatoshi ShojiGiacomo MugnaiSatoshi TakahashiGiacomo PapottoSatoshi YamaguchiGiacomo TiniSatoshi YuguchiGiacomo TondoSatya Ranjan MisraGiada BianchettiSatyanarayan RoutrayGiada CrescioliSaúl Gómez-ManzoGiamberto CasiniSaulius LukoševičiusGiammarco CascinoSaulius SvagzdysGiampaolo VettaSaumel AhmadiGiampiero NeriSaumya ShahGian Gaetano FerriSaurabh ChaturvediGian Luigi CanataSaurabh ChawlaGian Luigi NicolosiSaurabh LuthraGianandrea PasquinelliSaurabh RamBihariLal ShrivastavaGianantonio SaviolaSaverio IacopinoGiancarlo AldiniSaverio MuscoliGiancarlo MariSaviana GandolfoGiancarlo MichelettoSavio Domenico PandolfoGiancarlo ScognamiglioSavio George BarretoGiancarlo TodiereSavvas LampridisGiandomenico MaggioreSawan JalnapurkarGianfilippo CaggiariSawsan RashdanGianfrancesco FioriniSayali MukherjeeGianfranco FaviaSayantani ChowdhuryGianluca CaiazzoSayed Aliul Hasan AbdiGianluca CampoSayyed Ali SamadiGianluca CasseseScarfì FedericaGianluca CiardiScavello FrancescoGianluca CiolliSchohraya SpahisGianluca FaddaSchramm ReneGianluca FascioloScott A. ReadGianluca GaidanoScott HeltonGianluca NazzaroScott MatsonGianluca PaternosterScott McAlisterGianluca PiatelliScott T. McEwenGianluca PirasScozzari GitanaGianluca R. DamianiSe Hun KangGianluca Raffaello DamianiSe Jin ParkGianluca RompianesiSeamus O’ReillyGianluca SampietroSean CrawfordGianluca SerafiniSean MisekGianluca SottilottaSean X. GuGianluca TestaSebastiaan RemmersGianluca TrevisiSebastian D. SchaeferGianluca VillaSebastian FarrGianmarco AbbadessaSebastian KnitterGianmarco ArabiaSebastian M. JudGianmarco De DonatoSebastian MertowskiGianmarco IannopolloSebastian O. DeckerGianmaria D’AddazioSebastian RothGianmaria SalvioSebastian SchnaubeltGianna DiPalmaSebastian TimsGianni PaulisSebastián Valverde-MartínezGiannicola IannellaSebastian WawrockiGianpaolo RossiSebastian YuGianpaolo VaccarellaSebastian ZduńskiGiedrius BarauskasSebastian ZimmerGiia-Sheun PengSebastiano Alfio TorrisiGilad RegevSebastiano CiccoGilberto Pérez-SánchezSebastiano GiliGilda BrownSébastien BeauneGilda SandriSébastien FoulquierGiles CritchleySébastien MolièreGill A. Ten HoorŠebeštjen MiranGilles ChopardSebhat ErqouGilles GuerrierSebron W. HarrisonGilles MonifSeckin AkgulGillian L. S. SolesSecundino FernandezGillian LoweSedighe KarimzadehGil-Martínez MartaSeema GuptaGina GheorgheSefer ElezkurtajGina Maria CaetanoSeghieri GiuseppeGiner Galván VicenteSehi L’YiGino GrifoniSe-Hwa KimGioele CastelliSeifollah GholampourGiorgia AilunoSeigo OhbaGiorgia ComaiSeiichi NakagawaGiorgia GirottoSeiichi YoshimotoGiorgia GrassiSeiichiro MitaniGiorgia PepeSeika DenGiorgia VaralloSeisuke OkazawaGiorgio BergaminiSelami Aykut TemizGiorgio BerlotSelene BaroneGiorgio BernaSelvaraj JayaramanGiorgio BoganiSemih BaşçıGiorgio CalloviniSemra BilaçeroǧluGiorgio Enrico BravettiSemra DemirGiorgio FuianoSena TurkdoganGiorgio GiatsidisSenija EminovićGiorgio I. RussoSenka MeštrovićGiorgio Lo GiudiceŞenol TonyalıGiorgio LofreseŞenol TuranGiorgio LombardoSentaro KusuharaGiorgio PalandriSenthil BhoopalanGiorgio RadettiSenwei TsaiGiorgio SantoroSeok Kyeong OhGiorgio TregliaSeok-Jae KoGiorgos AlevizopoulosSeongbin HongGiovanna BorrielloSeong-Hun KimGiovanna CannasSeon-Yong JeongGiovanna Di GiannuarioSeoung Wan NamGiovanna GalloSepanta HosseinpourGiovanna IezziSerafim M. OliveiraGiovanna ManziSerban Constantin StoicaGiovanna MurmuraŞerban Vifor Gabriel BerteşteanuGiovanna PavoneSerdar YormazGiovanni BadialiSerena AmiciGiovanni Battista ForleoSerena Dell’AversanaGiovanni BrunoSerena LattanteGiovanni BuzzaccariniSerena PastoreGiovanni CannarozzoSerena SalomeGiovanni CenderelloSerge FerrariGiovanni ChiariniSerge MarbacherGiovanni CimminoSergey AvdeevGiovanni CrisafulliSergey GurevichGiovanni DamianiSergey KozlovGiovanni De AngelisSergey MoskvinGiovanni Dell`Aversana OrabonaSerghei CebotariGiovanni Donato AquaroSerghei CovantevGiovanni FalcicchioSergi Garcia-RetortilloGiovanni FanniSergi YunGiovanni GalatiSergio A. Mota-RolimGiovanni GaleotoSergio AmaroGiovanni GalloSergio Antonio TripodiGiovanni GalloneSergio BertiGiovanni GerbinoSérgio BrasilGiovanni GiordanoSergio ContiGiovanni GiurdanellaSergio Cuevas-CovarrubiasGiovanni L. BerettaSergio D’AddatoGiovanni La CannaSergio De SalvatoreGiovanni Maria ColpiSergio DellepianeGiovanni Maria ComacchioSergio FacchiniGiovanni Maria IannantuonoSergio FurgiueleGiovanni MatareseSergio HaimovichGiovanni MergoniSergio MaimoneGiovanni MisseriSergio MinucciGiovanni MotterleSergio MoralGiovanni NanoSergio OcchipintiGiovanni PalleschiSergio Piñeiro-HermidaGiovanni PapaSergio Ramón Gutiérrez-UreñaGiovanni PennisiSergio RecaldeGiovanni PerettoSérgio Rocha PiedadeGiovanni PutameSergio SalernoGiovanni RalliSergio SambataroGiovanni RollaSergio SchmidtGiovanni SalzanoSeri JeongGiovanni SansoeSerkan DündarGiovanni SantacroceSerkan UnluGiovanni ScambiaSerkan YílmazGiovanni TazzioliSeth CoreyGiovanni VitaleSeth T. HousmanGirardelli SerenaSeth TarrantGirish V. VajramaniSeun Deuk HwangĢirts BriģisSeung Eun LeeGisela MezquidaSeung Hee LeeGisella GuidoSeung Hwan KimGitta Anne JacobSeung Hyun LeeGiulia BrindisiSeung-Geun LeeGiulia CassoneSeung-Kook KimGiulia CossuSeungyup LeeGiulia D’AurizioSevcan BakkalogluGiulia Di DalmaziSeverin RodlerGiulia GaspariniSevgül DönmezGiulia GermenaSevilay Tümkaya YilmazGiulia MagnaniSeyed Ahmad Naseri-AlaviGiulia MenculiniSeyed Ahmad RasoolinejadGiulia MidenaSeyed Arash AlawiGiulia MorsicaSeyed Ehsan SaffariGiulia PaparellaSeyed Mehran FalahchaiGiulia SantoSeyed Mohammad Kazem AghamirGiulia SciosciaSeyit Ahmet ErolGiulia StronatiSeyyed Saeed KhabiriGiulia ZumboSezai YilmazGiuliana ScarpatiSh ZyoudGiuliano CostaShabeesh BalanGiuliano RamadoriShadab ShahaliGiulio CabrelleShadi H. TabibianGiulio Francesco RomitiShaghayegh Baradaran GhavamiGiulio GaviniShah-Hwa ChouGiulio GualdiShahid ZamanGiulio Maria Marcheggiani MuccioliShahin Abdollahi FakhimGiulio OlivieriShahla MirzaeeiGiulio RussoShahnawaz AnwerGiulio TessarinShahnawaz KhijmatgarGiulio VaraShahnaz ShahrbanianGiuseppa GraceffaShahzad IqbalGiuseppe AlloattiShai FactorGiuseppe AlonciShailendra Pratap SinghGiuseppe AmorusoShailendra S. GuravGiuseppe BarisanoShailendra SinghGiuseppe BarlettaShaji John KachanathuGiuseppe BasileShalini DhirGiuseppe BenagianoShalini DograGiuseppe BiaginiShambaditya DasGiuseppe BorianiShambhu YadavGiuseppe BorzellinoShameka L. CodyGiuseppe BronteShamik ShahGiuseppe BrunoShamir CassimGiuseppe CalabreseShamus CarrGiuseppe CaminitiShan ZhengGiuseppe CastaldoShane J. HavensGiuseppe CelentanoShang-Feng TsaiGiuseppe ChiarioniShan-Huey YuGiuseppe ComentaleShankargouda PatilGiuseppe D’AngeloShannon SalvadorGiuseppe De LucaShantanu GuptaGiuseppe Di PasqualeShaobin WangGiuseppe Emmanuele UmanaShao-Fang NieGiuseppe FanettiShaoning JiangGiuseppe G. LoscoccoShaoping NieGiuseppe GattiShao-Wen WengGiuseppe IettoShaoxun WangGiuseppe LanzaSharad ChandGiuseppe Lo GiudiceSharad ChandraGiuseppe LosurdoSharafaldin Al-MusawiGiuseppe LucarelliSharmilee M. NyenhuisGiuseppe MaccagnanoSharon AndersonGiuseppe MagliettaSharon CushingGiuseppe MagliuloSharon DavideskoGiuseppe MangiameliSharon Markham D. GeaghanGiuseppe Maria AlbaneseSharon SaldanhaGiuseppe MarraroSharon Yu Lin ChuaGiuseppe MasciaShatavisha DasguptaGiuseppe MigliarettiShauhuai FuGiuseppe MilitelloShaun J. KiltyGiuseppe MinerviniShaun RobinsonGiuseppe NassoShaunik SharmaGiuseppe PattiShazia BanoGiuseppe RemuzziShazid Md. SharkerGiuseppe RinonapoliShebli AtrashGiuseppe RivaShehwaz AnwarGiuseppe RossiShei-Dei YangGiuseppe RovereSheikh RiazuddinGiuseppe SignorielloSheila A. M. RauchGiuseppe SprianoSheila BermejoGiuseppe StabileSheila Sánchez CastilloGiuseppe UccelloSheila SpadaGiuseppe VezzoliSheilagh HodginsGiuseppe ZimmittiShekhar NeemaGiuseppina AmadoroSheldon GoldbergGiuseppina LaganàShemy CarassoGiuseppina MalcangiSheng ZhuGiuseppina MieleSheng-Hsiung SheuGiuseppina PalladiniSheng-Long DingGiuseppinella MelitaSheng-Min HsuGiusi Antonia TotoSheng-Po HaoGiusy GuzziShengshuai ShanGizem KeceliSheng-Tzung TsaiGjin NdrepepaShen-Han LeeGladiol ZenunajShepard HurwitzGladys HoSherif El‐MasryGlen CooperSherry MansourGlenda Giorgia CaputoSherry X. YangGlenn BaumanShevach FriedlerGlenn EastwoodShiao-Pieng LeeGlenn Matthew EastwoodShiau Foon TeeGlenn P. DorsamShibili NuhmaniGlenn PfefferShibo YuGleyson Kleber Do Amaral-SilvaShida ChenGloria AngelettiShigeaki NakazawaGloria BonaccorsiShigehiko OgohGloria CalagnaShigekazu SuginoGloria ChanShigeki MatsubaraGlória ConceiçãoShigemitsu SakumaGloria Ivy MensahShigenaga MatsuiGloria PryhuberShigeo MuroGloria SantangeloShigeru MaedaGniewko WięckiewiczShigeru ShibataGo AnanShihai LiGo TajimaShih-Chi KuGökçen Dilşa TuğcuShihlung ChenGokhan OzunerShih-Min HsiaGokhan SarginShiho NaitoGokul KrishnaShihua BaoGoncalo BarretoShih-Wei HuangGonghui LiShih-Yuan HungGonzaga Garay-AramburuShijun WeiGonzalez-Hormazabal PatricioShikha Talwar BassiGonzalo VarelaShilpa BhandiGoran AugustinShilpa KodatiGöran LarsonShimin ZhuGoran VrgočShin GotoGorazd PojeShin Jun ParkGordana Blagojevic ZagoracShin MatsuokaGordana ŽauharShin MorizaneGordian Jan HubertShingen NakamuraGordon BarbaraShingo KuwataGordon L. KleinShingo YamamotoGotam DasShin-Hoo ParkGoto YoshikazuShinichi EgawaGottfried LemperleShinichi HashimotoGottfried WechselbergerShinichi KinamiGötz FriedrichShinichiro NagamitsuGowtham Kumar AnnarapuShinji MorimotoGözde Kandemir DemirciShinji OtaniGözde Şengül AyçiçekShinji TeramotoGrace Y. LamShinnichi OkazumiGraciela Alatorre-CruzShinnkuang LinGraeme WalkerShinsaku ImashukuGraham R. WallaceShintaro AkiyamaGrant MairShintaro FujiharaGrasiele SausenShintaro HorieGrazia PaviaShintaro NakanoGrazianna CollaianniShintaro SukegawaGraziano MontaruliShinya KowaseGraziela De Luca CantoShinya MatsuzakiGraziella TuratoShinya OkazakiGrażyna DykowskaShinya TomariGrazyna GosciniakShir Lynn LimGrażyna JanikowskaShiran ShettyGrażyna NowickaShirit Kamil-RosenbergGrażyna SygitowiczShirley D’SaGrażyna Wyszyńska-PawelecShirley RussGreg HoyShirli WernerGreg Neil BowdenShiro MizunoGregor BaraShiro NakamuraGregor Sebastian ReiterShishir Ram ShettyGregory HeathShisong RongGregory P. BartonShiunwoei WongGregory W. AlbertShivam MehtaGreta MainieriShivanand PudakalakattiGretha J. BoersmaShivani BansalGrigorios G. DimasShivshankar ThanigaimaniGrigorios NasiosShixiang SunGrigorios TsigkasShi-Xing TangGro Dehli VillangerShizuko NagaoGrzegorz BielecShmuel EinyGrzegorz GrześkShobana NavaneethabalakrishnanGrzegorz HelbigShogo HayashiGrzegorz JakielShohei AkagawaGrzegorz JakubiakShoji OuraGrzegorz KoniecznyShoji TakenakaGrzegorz KowalewskiShoko HaraGrzegorz KudelaShota TakashimaGrzegorz ŁabuzShotaro NaganawaGrzegorz NowickiShoumyo MajumdarGrzegorz PiechaShravan MorlaGrzegorz SławińskiShreyas D. GowdarGrzegorz SobieszekShruti ChandraGrzegorz TrybekShuaijie WangGrzegorz WysiadeckiShubha Ranjan DuttaGrzegorz ŻurekShubhi KaushikGuadalupe Alvarez HernanShuhama RosanaGuadalupe Molina TorresShuhei YoshidaGuangyu ZhuShu-Hui WenGuglielmo ManticaShu-I. WuGuglielmo Niccolò PiozziShuichi OnoGuglielmo RamieriShuichiro AokiGuglielmo SaittoShuji OzakiGuglielmo StabileShujiro HayashiGuido ManfrediShu-Jui KuoGuido MühlmeierShumaila Aman TasneemGuido VanhamShun IshibashiGuido ZavattaShunichi FukudaGuilherme De Freitas FonsecaShunji SuzukiGuilherme Grossi Lopes CançadoShunsuke SaitoGuillaume Anthony OdriShunsuke YamadaGuillaume BaudryShusuke UedaGuillaume BonnaudShuta OharaGuillaume BonnetShuzo HamamotoGuillaume BrocShuzo SatoGuillaume DucarmeShweta RamdasGuillaume LefebvreShyam Bihari BansalGuillem Muntané-CarolShyam GajavelliGuillem Pintos-MorellShyam MoreGuillermina CuadradoShyikuen WuGuillermo García-AlíasSiamak SalavatianGuillermo PlazaSian Andrea WilliamsGuillermo Ruiz-ArgüellesSibtain AhmedGuillermo Til-PerezSiddarth AgrawalGuinevere GraniteSiddharth DugarGuiomar Perez De NanclaresSiddharth NathGül Fatma YarımSiddharth PahwaGülçin Özalp GerçekerSiddharth SunilkumarGülderen Yanikkaya DemirelSiddig AbdelwahabGülendam KaradağSiegmund LangGulletta SimoneSiew-Keah LeeGülpembe BozkurtSigal PortnoyGulzhanat AimagambetovaSigit PrastowoGunnar KristensenSigmar SchnutenhausGüntülü AkSigrid I. KvaalGuoguo YiSigrun ThorsteinsdottirGuohua DingSijie ZhengGuoju HongSijo MathewGuoli DaiSilmara Marques AllegrettiGuoli ZhengSilvana Alves PereiraGuowei KimSilvana Artioli SchelliniGuram ImnadzeSilvana MiceliGurdeep SinghSilvano DragonieriGurkan MollaogluSilvano Junior SantiniGurmukteshwar SinghSilverio RotondiGursukhmandeep SidhuSilvia A. OddoGurusaravanan Kutti SridharanSilvia AkutsuGustavo ArocaSilvia Alberti-ViolettiGustavo De Conti Teixeira CostaSilvia BarbonGustavo Fabián MolinaSilvia BroginiGustavo FernandesSilvia CastellettiGustavo Rodríguez FuentesSilvia De Miguel-BilbaoGustavo Vieira De OliveiraSilvia DelrioGusztav BeltekiSilvia E. Perez-ProttoGuy DecauxSilvia FilippiGuy MagalonSilvia FlorescuGuy RostokerSilvia Gianoni-CapenakasGwendolin Manegold-BrauerSilvia GiovanniniGwenllian TawySilvia GonellaGyörgy SzabóSilvia Grazietta FoddaiGysbert Botho Van SettenSílvia Maria Amado JoãoGytė DamulevičienėSilvia MarianiH. Denis AlexanderSilvia MascoloH. M. Ayala S. HerathSilvia Mas-PeiroHabib Md Reazaul KarimSilvia PalmaHadi Kazemi-ArpanahiSilvia PancaniHadi Ufuk YörükoǧluSilvia PianigianiHae-Young Lopilly ParkSilvia PizzighelloHafize Emine SönmezSilvia RocchiccioliHaggi MazehSilvia SpivakovskyHahn Young KimSilvia Zupančić ŠalekHai Long WangSilvija ŠoprekHai YuSilvio CaravelliHaibo WuSilviu Horia MorariuHaidong LuSimcha R. MeiselHaipeng LiuSimin SharifiHaipeng XiaoSimon AllenHaisheng YangSimon Christopher BodyHaishu LinSimon HorslenHaitham JahramiSimon John DaviesHaiyang A. YuSimon KralerHaiyun LiSimon Morand-BeaulieuHajime IchikawaSimon N. BellHajime TakatoriSimon PodnarHak ChangSimon SchoechlinHåkan N. PärssonSimon T. AbramsHakeem O. AyindeSimon VilleHala MuaddiSimon W. RabkinHalil İbrahim CeylanSimon WilkinsHalil Ibrahim TanriverdiSimona BadilescuHalina Cichoz-LachSimona BorstnarHalis SüleymanSimona Catalina StefanHamayak S. SisakianSimona CensiHamdi Ben HalimaSimona DurantiHamdi ChtourouSimona FerraroHamdy AbdelkaderSimona Maria BatagaHamid HassanzadehSimona NicoaraHamid M. SaidSimona PergolizziHamid PakmaneshSimona Roxana GeorgescuHamid Reza KatoozianSimona RuțăHamid Reza MoeinSimona Simona FioriHamid Riazi-EsfahaniSimona SperlonganoHamimatunnisa JoharSimona TavecchioHammad A. GanatraSimone Alex BagagliaHamza Naciri BennaniSimone BiniHan MeiSimone BirocchiHan Naung TunSimone ConciHan Siong TohSimone CrouchHana FakhourySimone Di PlinioHana ŠtudentováSimone Domenico AsprielloHanaa ShafiekSimone DonatiHanan ElajailiSimone Dos Santos BarretoHande Mefkure OzkayaSimone GalloHani J. MarcusSimone GrandiniHania Salah ZayedSimone GrassiHan-Mou TsaiSimone La PadulaHanna DanielewiczSimone LazzeriHanna HuebnerSimone MorraHanna KuusistoSimone MorselliHannah H. ChiuSimone PaliniHannah M. KilianSimone PersampieriHannah StevensSimone ReppermundHannah WozniakSimone SampaoloHann-Chorng KuoSimone SavastanoHannes JacobsSimone ScarcellaHanns HelmerSimone SerafiniHanns-Christian BreitSimone SforzaHans C. StubbeSimone TzaridisHans CrezeeSimone WajnerHans HenkesSimonetta MorselliHans I. WolfSimote FoliakiHans P. SviggumSina GrapeHans PasterkampSina HulkkonenHans Peter DietzSina KohlHans TroppSina M. HulkkonenHans Van EyghenSina RashediHans WolfSina TaefehshokrHans-Gert HeuftSinan DemircioğluHans-Joerg TrnkaSinan TanyolaçHans-Michael KaltenbachSingh ArshdeepHans-Oliver RennekampffSinisa DjurasevicHans-Peter BischofSinjini SikdarHans-Peter EberhardSiobhán MastersonHans-Peter WiesingerSiqi HuHans-Ulrich BenderSira NanthapisalHany MareiSiri-Maria KampHao ChenSitthichok ChaichuleeHao Chun TsaiSiu Wai ChoiHao LiSiva Prasad PandaHao YuSivabaskari PasupathyHaobijam BasantaSivapong SungpraditHao-Lin SunSiwei ZhangHaomin LiuSjoerd TimmermansHaotsai ChengSkaidra ValiukevičienėHaralampos M. MoutsopoulosSkaidrius MiliauskasHarald KrentelSlavica KvolikHarald Kurt WidhalmSlavoljub ŽivkovićHarald VogelsangSławomir Cezary ZmonarskiHaresh SelvaskandanSlawomir ChlabiczHari Siva Gurunadha Rao TunuguntlaSławomir CisieckiHariharan RegunathSławomir KoziełHaripriya GuptaSlawomir TeperHaris MemisevicSławomir WołczyńskiHarish ChanderSleiman Sebastian Aboul-HassanHariyono WinartoSlobodan MaricicHarleen ChelaSlobodan ObradovicHarold L. RekateSlobodanka Lemajic-KomazecHarold Yeehau LauSmaranda BuduruHaroon AwanSmita JhaHarri H. HakovirtaSmita NairHarriet MorfSmriti Murali KrishnaHarris S. MichaelSneha SinghHarrison J. StrattonSnehil DixitHarry AcquatellaSo Jung MunHarry V. M. SpiersSo NagaiHarshani WijerathneSo Ree KimHarshraj LeuvaSoban QadirHartmut KuhnSobuj MiaHartmut VatterSocorro Retana-MarquezHaruka FujinamiSoedarsono SoedarsonoHarukazu HiraumiSofia ChatziioannouHarumasa YokotaSofia FranciaHarun Egemen TolunaySofia MacedoHaruna KawanoSofia N. NyströmHarvey DillonSofia VasilakakiHarvinder Singh ChhabraSofia Villacis-NunezHasan GündoǧarSofia WaissbluthHasan NaveedSofoklis MitsosHasan SymumSofya MorozovaHasegawa TomonobuSofya P. KulikovaHashem Hammouda GhorabaSoha AlbeitawiHashim Talib HashimSohail SyedHasnaa JalouSoheb Anwar MohammedHassan PatailSoheil Abbaspour-RavasjaniHaugaard SbSoheil MohammadiHavva KocayigitSo-Hyun NamHaya SaturninoSoichiro EbisawaHayate NakagawaSoichiro EnomotoHayato GoSoichiro TakamiyaHayato TadaSokol TrunguHayato TakahashiSokratis GrigoriadisHaydeé Rosas-VargasSola HanHayley BarnesSomaira NowsheenHayley L. LetsonSomasundaram PrasadhHayoung ChoiSomchai AmornyotinHazem M. MousaSomdet SrichairatanakoolHazem OmranSomenath ChakrabortyHe S. YangSomsak SittitavornwongHe SunSoňa ČiernikováHeather Angus-LeppanSonal AgrawalHeather BurksSonam KumariHeather Gordish-DressmanSonam YangzesHeather Gwen MackSong HuangHeather HadjistavropoulosSong WanHeather LaneSongyi ParkHeather ZwickeySongyoung ParkHector BonillaSonia D’ArrigoHector FerralSonia FantoneHéctor González-de La TorreSonia GuptaHector Vilca-MelendezSonia RomanHee Sam NaSonia RoncheyHee Yong KangSonia SharmaHee Young LeeSonika PuriHeejin KimSonja BuvinicHeena KumraSonja HeiblHege Kampen PihlstrømSonja HochmeisterHeidi AltmannSonpavde GuruHeike ArgstatterSonu Menachem Maimonides BhaskarHeike EndepolsSonu SubudhiHeike RothSonya NaganathanHeikki O. KoskelaSoo-Hyun SungHeiko LocherSoo‐Kyoung ChoiHeiko SternSoo-Yeon KimHeiner WoltersSophia DelicouHeinrich ReschSophia M. SchmitzHeinrich ScheiblauerSophia N. SangiorgioHeinz KölblSophia Tsong Huey ChewHeinz‐Peter SchulteissSophie CaldérariHeitor Andrade Jr.Sophie Collardeau-FrachonHélder LopesSophie DeguelteHelen E. TurnerSophie MullerHelen GrechSoren U. NielsenHelen HurstSorin OlariuHelen P. KoureaSorin SieglerHelen PergantouSorin Tiberiu AlexandrescuHelena Ban-FrangežSorina Grisaru-GranovskyHelena IznardoSotirios ApostolakisHelena LingeSotirios G. ZarogiannisHelena M. GardinerSotirios GiannopoulosHelena Maria Tannhauser BarrosSotirios SotiriouHelena MartynowiczSouhail HermassiHelena MartyowiczSoumaya BoudokhaneHelena Santa-ClaraSoume BhattacharyaHélène RouardSoumojit PalHelene SpeyerSoundappan S. V. SoundappanHeleni VastardisSourav PatnaikHelge Rask-AndersenSousa Freitas Queiroz NatáliaHelios De RosarioSousana PapadopoulouHelmar Bornemann-CimentiSouthern Danielle AHelmut G. WeißSouvick RoyHem Dutt ShuklaSoyar SariHemant JainSoyoung KwakHemant KulkarniSpandana NaldigaHendrik S. FischerŠpela ŠalamonHendrik UngefrorenSpencer BehrHenglong HuSpencer P. LakeHenk S. BrandSperanza EspositoHenri AfghahiSpyrido KatsanosHenrik C. BäckerSpyridon KanellakisHenrik FalhammarSrdjan B. AleksandrićHenrike HillmannSrebro DraganaHenrique Andrade FonsecaSreedhar Reddy SutheHenrique Furlan PaunaSreenivas KarnatiHenrique José CardosoSreeparna ChakrabortyHenry Anselmo MayalaSricharan S. VeeturiHenry CohenSridevi PadmanabhanHenry LiuSridevi R. PittaHenry P. GodfreySriganesh Ramachandra RaoHenry PleassSrijithesh Rajendran SrijitheshHenry PownallSrikanth UmakanthanHenry SutantoSrinivas AluriHenryk KasprzakSrinivas DwarakanathHenryk SkarzynskiSriram GubbiHerbert ChidambaramSrjit DasHerbert LöllgenSrujana SahebjadaHerbert ValensiseStacey MeardonHeribert RamrothStaffan WahlinHerjan J. T. Coelingh BenninkStamatia TheodoridouHerko GrubitzschStamatis GregoriouHerman E. PopeijusStanislav KotlyarovHermann BlessbergerStanislav PekarskiyHerminia Brites DiasStanislav ZiaranHernando TrujilloStanisław HorakHerve BoutinStanisław KwiatkowskiHervé LobbesStanisław NiemczykHervé ReychlerStavroula IliaHerwig CerwenkaStefan BourgeoisHesam HashemianStefan Cristian VesaHester VlaardingerbroekStefan D. MomčilovićHiba NatshehStefan DoerrHideaki KawabataStefan EversHideaki NakajimaStefan Felix EhrentrautHideaki ShigaStefan HadzicHidehiro OkuStefan HummelinkHidehito KimuraStefan J. LangHidekane YoshimuraStefan KnopHidekazkeu MoriyaStefan KranzHideki FujiiStefan Kurath-KollerHideki KawaiStefan M. FroschauerHideki S. SudoStefan Marian FrentHideki SekiyaStefan MarkartHideki WadaStefan MihaicutaHidemi NakataStefan NaydenovHidemichi KouzuStefan OrwatHidenori SuzukiStefan PalkovitsHidenori TakahashiȘtefan PătrașcuHideo WadaStefan SammitoHidetada FukushimaStefan ScholzHidetaka HamasakiStefan ToggweilerHidetaka NomaStefan WeberHidetaka TamuneStefan ZielenHideto NakajimaStefania CamagniHidetomi TeraiStefania CanovaHidetoshi KomatsuStefania CarlucciHideyuki KinoshitaStefania CimbanassiHideyuki MaedaStefania FerrettiHideyuki ShiomiStefania LeuciHideyuki YoshitomiStefania MagdaHimangshu SonowalStefania MarsiliHimanshu BatraStefania PrincipeHimanshu DeshwalStefania TroiseHinhin KoStefania ZampattiHinne A. RakhorstStefania ZanettiHinrich StaeckerStefanie A. WoodardHipolito NzwaloStefanie Kennon-McGillHiral MasterStefano AlbaniHiroaki IchijoStefano BallestriHiroaki KijimaStefano BenenatiHiroaki NagaseStefano BibbòHiroaki NakagawaStefano CanosaHiroaki NekiStefano CarugoHiroaki TobaStefano CiardulloHiroaki YokoteStefano CirilloHiroaki YoshikawaStefano CoaccioliHiroe OhyamaStefano DastoliHirofumi AndoStefano FestaHirofumi ImaiStefano Francesco CrinòHirohito IshigakiStefano FraraHirokazu InoueStefano FratiniHirokazu ToyoshimaStefano GennaiHiroki IdeStefano GhioHiroki KabataStefano GhirardelliHiroki NishikawaStefano GiarettaHiroki SatoStefano GonnelliHiroki TeragawaStefano LuzzagoHiroki WakabayashiStefano MarchettiHiroko KobayashiStefano Marco Paolo RossiHiroko TerashimaStefano Maria PriolaHiromasa HasegawaStefano MariniHiromichi YumotoStefano MartinaHiromitsu NegoroStefano MoalliHironari TamiyaStefano MoiaHironobu TodaStefano NecozioneHironori IshiguchiStefano NistriHiroshi HoriuchiStefano OlgiatiHiroshi InuiStefano PalermiHiroshi IrisawaStefano Piero Bernardo CioffiHiroshi IwamotoStefano RealeHiroshi MukaeStefano RestainoHiroshi NoguchiStefano SalgarelloHiroshi NonoguchiStefano SavinelliHiroshi TanakaStefano SchenaHiroshi UreshinoStefano SebastianiHiroshi WakabayashiStefano SpieziaHiroshi YonekuraStefano TaboniHirotaka HasegawaStefano TambuzziHirotaka NakashimaStefano TavanoHirotaka SawanoStefany PrimeauxHirotaka YadaSteffen GrautoffHirotoshi KikuchiSteffen HauptmannHirotsugu FukudaSteffen RosahlHiroya AkaboriSteffen WagnerHiroya KuroyanagiStein Gunnar LarsenHiroyoshi DoiSteliana GhibuHiroyoshi MoriSten RasmussenHiroyuki DaidaStepan GambaryanHiroyuki HanadaStepan HavranekHiroyuki HiraiStephan AltmayerHiroyuki IsayamaStephan FuhrmannHiroyuki NiidaStephan HalleHiroyuki ShigetaStephan HeisingerHiroyuki TakahashiStephan HohmannHiroyuki UetakeStephan KemmnerHiroyuki YamadaStephan LauxmannHiroyuki YamaguchiStephan LeisengangHisami AndoStephan Ong ToneHisao ImaiStephan PayrHisham AltayebStephan ReshkinHisham FansaStephan StoebeHitesh VijStéphan TroyanovHitoshi AndoStephanie A. BergHitoshi HachiyaStéphanie BaronHitoshi KagayaStephanie DramburgHiu Chuen LokStephanie E. SillivanHo Seok SeoStephanie L. SellersHoda HatoumStephanie R. AlbinHolger LoessnerStephanie Rodríguez BesteiroHolger MuehlanStephanie S. BussHollis O’NealStéphanie SigautHolly A. ShillStephanie T. YerkovichHomayoun TabandehStephen A. ContagHonda HsuStephen A. ZdericHong Jin KimStephen Andrew VernonHong MaStephen ContagHongbo DuStephen D. GillHonghe LiuStephen D. RyderHongmin ChuStephen E. ConglyHongping JinStephen FalkHongshik AhnStephen FavaHongsun GuoStephen HollyHongying WangStephen InnsHong-Yuan HuangStephen KellettHongyuan ZhangStephen PanHoracio Matías CastroStephen R. BakerHoraţiu MoldovanStephen StrumHorea FeierStephen X. ZhangHoria BumbeaSteve HyerHoria ManoleaSteven BaumruckerHoria StancaSteven BrennanHorst Von BernuthSteven De ReuverHortensia Moreno-MacíasSteven E. WilliamsHosam ShehaSteven J. FlieslerHoshi SujinSteven KnapperHossein AziziSteven LevyHossein MotedayyenSteven LohHossein TabatabaeianSteven M. KoehlerHossein ZarrinfarSteven M. ParnesHouman KhaliliSteven PorterHouman SotoudehSteven RogersHo-Yeon KimSteven S. SpiresHrayr AttarianSteven SilversteinHridesh Kumar MishraSteven WolfHristo Mihaylov KirovStian AlmelandHsiangkuo YuanStryder MeadowsHsiang-Ning LukStuart LaveryHsin-Yao WangStuart McCluskeyHsin-Yen YenStuart RundleHsin-Yi YangStuart SnowdenHsiu-Ching ChiuSturm TimoHsiulin ChenStuti MisraHsueh-Ju LuStylianos TomarasHsueh-Yu LiSuat SaribasHsu-Heng YenŞuayip Burak DumanHsun-Liang ChanSubendu SarkarHua ZhuSubhash MedhiHuaishuang ShenSubir Kumar JuinHuaitian LiuSu-Boon YongHuan GuSuchada KongkiatkamonHuan-Gan WuSuchith ShettyHuang-Pin WuSuchitra JoshiHuang-Ping YuSudarshan BhattacharjeeHubert KameckiSudhir AggarwalHubert MarotteSudhir KrishnanHubertus AxerSudhir KshirsagarHueliton Wilian KidoSudi PatelHugh GashSudipta BiswasHugo H. OrtegaSue L. HallHugo Martínez-RojanoSue PlummerHugo Pena-VerdealSueli Coelho Da Silva CarneiroHugo Rodriguez-ZanellaSufang LiuHui ZhaoSufaru Irina GeorgetaHui-Ching ChuangSuhaib J. S. AhmadHuiChuan YuSuhail Sarwar SiddiquiHui-Ju LinSuhaila Abd MuidHui-Ling LinSu-Ho LimHumberto HernandezSuhwan LeeHun LeeSuisui HaoHung-Che LinSuixin LiuHung-Chih ChiuSujin LeeHung-Da ChouSujit Kumar TripathyHung-Jen ChenSujith PereiraHung-Jen WangSujun ZhengHung-Pin TuSukhmani BediHung-Wei WangSukhvinder SinghHungwon TchahSu-Kiat ChuaHung-Yang ChangSukkum ChangHung-Yu ChangSulagna SaittaHun-Ju YuSüleyman Cemil OğlakHuseyin EdeSuling HuangHüseyin M. AyhanSultan Ayesh Mohammed SaghirHuseyin TarhanSultan TousifHussain Yar KhanSumadi Lukman AnwarHussam AliSumaiah AlarfajHussein AbdelazizSuman ChaudharyHwa-Jung KimSuman DuttaHye Chang RhimSumana GhoshHye Seon KangSumche ManHyeck-Soo SonSumit SohalHye-Min SohnSun Tae AhnHyeongwon YuSun Young JangHyeri SeokSunee ChansangpetchHyeung-Ju ParkSung Eun KimHye-Yon ChoSung Eun ParkHyo-Jeong LeeSung Huang Laurent TsaiHyo-Joon YangSung Mi HwangHyojune KimSung Soo AhnHyo-Jung JeongSung-Chul LimHyoungmin KimSung-Ho HerHyuk-Joong ChoiSunghoon ShinHyun Jin ChoiSunghyun PaickHyun Joon KimSung-Kyo KimHyungju KwonSungmin LimHyungon LeeSung-Sahn LeeHyung-Youl ParkSung-Whan KimHyunil LeeSunil K. NadarHyun-Jin MinSunil S. KarhadkarHyun-Min SeoSunil VernekarHyun-Sun ParkSunita KeshariHyun-Yoon KoSun-Jai KimIacopo GesmundoSun-Joo JangIain C. CampbellSunny Chi Lik AuIain Parry HargreavesSupanji SupanjiIakovos AmygdalosSupavit ChesdachaiIan C. FrancisSupreeth P. ShashikumarIan DeSouzaSupriya SharmaIan JenkinsSupriyo RayIan JudsonSurachai Sae-JungIan M. ThornellSuranga KodithuwakkuIan MorganSurendra RajpurohitIason KyriazisSurendra ShuklaIbolya CsecsSuresh KalathilIbrahim BozgeyikSuresh KeshavamurthyIbrahim M. SayedSuresh NarvaIbrahim MaraiSurya KantIbrahim Y. AbdelgawadSusan J. JenkinsIbtissam AcemSusan L. HutchinsonI-Cheng LuSusan M. SheaIchiro KukitaSusana C. PenasIchiro TakajoSusana M. CerritelliIchiro YamadaSusana ObregonIda CariatiSusana OchoaIda PastoreSusana Sangiao-AlvarellosIdan GorenSusanna M. C. KauhanenIdit MaharshakSusanna NuvoliIdo FeferkornSusanna ProudmanIftikhar Ali KhawarSusanne Greber-PlatzerIgnacio Díez LópezSusanne KimeswengerIgnacio E. TapiaSusanne R. KerscherIgnacio Martínez-González-MoroSusanne StickelIgnacio N. RetamalSushil ChaudharyIgnacio TartaraSushma TejwaniIgnatios IkonomidisSushruth EdlaIgnatius NipSuvaporn AnugulruengkittIgnatius Ryan AdriawanSuwasin UdomkarnjananunIgnazio CondelloSuyanto SuyantoIgor A. DyachenkoSu-Ying TsaiIgor A. ZolotukhinSuzanne HolewijnIgor Age KosSuzanne KatsIgor BednarskiSuzanne LesageIgor DumicSuzuki ShugoIgor FischerSven F. GarbadeIgor KhatkovSven GuntherIgor OscorbinSven LichtenbergIgor VendraminSven NiklanderIgor Y. IskusnykhSven StieglitzI-Jung TsaiSven VetterIker J. BautistaSvenja Dannewitz ProssedaIkhwan RinaldiSvenja KoslowskiIkhwanuliman PuteraSven-Oliver TröbsIkhyun JunSverre Morten ZahlIkjoon MoonSvetla TodinovaIkkei TamadaSvetlana IvanovaIlaha IsaliSvetlana RatchinaIlan BruchimSvetlana SimićIlan MerdlerSvetlana V. MustafinaIlaria CampioniSvetlana Yu. ChikinaIlaria CerasoliSwapna Mahurkar-JoshiIlaria De BenedettoSwapnil Ganesh SanmukhIlaria De SimoneSwapnil HiremathIlaria FantoniSwastik PhuleraIlaria GrassiSwathi SangliIlaria GuerrieroSweta B. PatelIlaria IacobucciSyazrah SalamIlaria MarcantoniSyazwan SaidinIlaria PatiSyed Adeel HassanIlaria RiboldiSyed Arman RabbaniIlaria RighiSyed BukhariIlaria RivoltaSyed Faisal ZaidiIlaria SimonelliSyed Hani AbidiIlaria Tocco TussardiSyed MehdiIleana ConstantinescuSyed Mohammed BasheeruddinIlhan InciSyed NabilIlias MahmudSylvain DurandIlias P. DoulamisSylvain IcetaIlias SkeparniasSylvain LamureIlja VietorSylvain ManfrediIlke Coskun BenlidayiSylvain Royİlknur KaleliSylvère Störmannİlknur Kıvanç AltunaySylvester Reuben OkekeIlo E. LeppikSylvester Yao LokpoIlonka Kreitschmann-AndermahrSylvia E. BadonI-Lun HsinSylvia T. SingerIlya KlabukovSylwia ŁaganIlya L. GubskiySyoichi TashiroIlya M. MukovozovSzabolcs VarbiroIlyes BenlalaSzczepan PiszczatowskiIlze ŠtrumfaSzczepan WiechaImadeldin ElfakiSzu-Tah ChenImam AmmarullahSzymon DarochaIman AganjSzymon JanczarIman TavassolySzymon Łukasz DraganImed LatiriSzymon SkoczynskiImeke GoldschmidtSzymon SkoczyńskiI-Mo FangTadahisa InoueImoigele P. AisikuTadahisa SugiuraImtiyaz Ahmad WaniTadamichi AkagiIn Ah ChoiTadashi FuruyamaIna SevicTadashi ItoIna Valeria ZurloTadashi NakamuraIñaki LeteTadashi SakaguchiInar CastroTadatsugu MorimotoInas K. SharquieTadayuki OshimaInchang HwangTadeja KuretÍndia Olinta De Azevedo QueirozTadeusz DerezinskiIndrani PoddarTadeusz PrzybyłowskiIndravadan PatelTadeusz RobakIndre Bileviciute-LjungarTae Eun LeeInés Aguinaga-OntosoTae Ha ChungInes Bilic-ĆurčićTae Han KimInês FerreiraTae Hoon KongInés Jiménez VarasTae Jin YunInes KovačićTae Keun OhInes KožuhTae Keun YooInes MarongiuTae Kon KimInes MonteTae-Hwan KimInês SantosTae-Jin KimInez BronsveldTae-Min RheeInger UhlenTae-Soo ChoiIngo TodtTae-Yoon LeeIngo VolkmannTaeyoung KongIngrid Fricke-GalindoTaghred M. SaberIngrid MartonTaha Koray ŞahınIngrid PrkačinTahereh JavaheriIngrid TonhajzerovaTahereh ShokohiIngrid TonniTahir AnsariIngrida BalnyteTahir NooraniIngunn DybedalTahir ShahIngvíld E. BirschmannTahmineh MokhtariInki MoonTaihei ItoIn-Lu Amy LiuTai-Heng ChenInmaculada Bautista CastañoTai-Hsien WuInmaculada C. Palomo-ToucedoTaira FukudaInmaculada RiquelmeTaja RamutaInnokentii E. VishnyakovTajana FilipecIoan Alexandru FlorianTakaaki InoueIoan SarbuTakaaki MurakamiIoana Cristina DahaTakaaki SatoIoana GrigorasTakaaki TsuchidaIoana LeseTakafumi NakayamaIoana Marina GrinţescuTakafumi SatoIoana-Mihaiela CiucaTakafumi YokotaIoanna DimopoulouTakahiro OtsudoIoannis A. KalogiannidisTakahisa GonoIoannis A. TsolakisTakahito KatanoIoannis AnagnostopoulosTakahito OhshiroIoannis D. LaoutarisTakamasa MiyauchiIoannis E. KougioumtzisTakamichi KuwaharaIoannis HalkiadakisTakanobu HirosawaIoannis IoannidisTakanori MorikawaIoannis KatsarosTakanori YasuIoannis KopsidasTakashi HiraiIoannis LiampasTakashi KohnoIoannis M. AslanidesTakashi KokudoIoannis N. BoletisTakashi MatsushimaIoannis P. AlexanianTakashi MinegishiIoannis PanagouliasTakashi OgawaIoannis PapadiochosTakashi OhtsukaIoannis ParaskevaidisTakashi OnoIoannis PetrakisTakashi SuzukiIoannis SavvasTakashi TakedaIoannis SkalidisTakashi TanakaIoannis T. KonstantinidisTakaya HigakiIoannis VasileiadisTakayasu MishimaIoannis VentoulisTakayasu OhtakeIoannis VlastosTakayuki MasakiIolanda AquilaTakeji SaitohIolanda PopaTakemichi FukasawaIolanda Valentina PopaTakeo FuruyaIonut DonoiuTakeshi KawasakiIosif MarincuTakeshi KokubuIppei MiyataTakeshi NakaharaIraklis TsangarisTakeshi OgawaIrawaty DjaharuddinTakeshi UnokiIrena GlažarTakeshi YagyuIrena Gribovskaja-RuppTaketo KurozumiIrena JekovaTaketo YamadaIrena MilaniakTaku AokiIrena MladenovaTakuhei ShojiIrena TabainTakuhiro MoromizatoIrena WaleckaTakuji IwashitaIrena Wojsyk–BanaszakTakuji YokoeIrene BiasoliTakuma InagawaIrene CampiTakuma MatobaIrene Cortés-PérezTakumi InamiIrene FuscoTakumi TakeuchiIrene GagliardiTakumi TsujiIrene KarampelaTakuro KoboriIrene OttavianiTakuro ShirasuIrene R. DeganoTakuya KoieIrene ScalaTakuya MiyagiIrene SoletoTakuya NakaiIrfan A. RatherTakwa GabrIrfan KhanTal GilboaIria Da Cuña-CarreraTal LevinsonIrina EsanuTalia L. FuchsIrina GradinaruTalida Georgiana CutIrina Iuliana CostacheTam PaulIrina Ivanova IvanovaTamara K. NowlingIrina KiselevaTamara Pawlaczyk-KamienskaIrina KondakovaTamara PecherinaIrina LarionovaTamara SerdinšekIrina TsibulakTamás BaranyaiIrina VierasuTamás ConstantinIrini P. ChatziralliTamás DócziIris HolzerTamás F. MolnárIris ParriniTamás HaideggerIris Z. UrasTamás MajorIrit HochbergTamás OllmannIrit KrauseTamas SzakmanyIrma DumbryteTamer SeckinIrmgard TegederTamerah HuntIrmina NahonTami BornemanIrshad Ahmed HajamTami Brutman-BarazaniIrteja IslamTammy KindelIryna SamarskaTamra RanasingheIrzal HadžibegovićTandy Hastings-IsonIsaac NgereTang-Chuan WangIsaac RheeTania Ahalya ThimrajIsaac Seow-EnTânia Silvia FrödeIsabel AndiaTania TriantafyllouIsabel BesharTanima DeIsabel Corrales GutiérrezTanja GrotenIsabel Escobio-PrietoTanja HüschIsabel M. McFarlaneTanja J. MiličićIsabel Rodríguez CostaTanja Premru-SršenIsabel Rubio-AliagaTanja VeljovicIsabel V. FigueiredoTankeshwar BoruahIsabell HaaseTanu WadheraIsabella AquilaTanuj WadhiIsabella LurjeTanushree DangiIsabella PisaniTanveer SinghIsabelle LalemanTanya J. LehkyIsabelle PiazzaTanya L. GlennIsabelle SerrTanya StratevaIsain ZapataTanzil Mahmud ArefinIsam AtroshiTanzila SabaIsamu HokutoTao YangIsao OkayasuTara DonkerIsao OtsukaTarek ElshebinyIsela Montúfar-RoblesTarik KivrakIshag I. AdamTarik Sqalli HoussainiIshan BhattTariq MalikIshita BasuTaro ArimaIşın Yagmur CombaTaro TakeuchiIslam AbdelrahmanTaro YamashitaIslam AliTartaglia Gianluca MartinoIsmael Sánchez-GomarTarun DaliaIsmaheel O. LawalTarvinder Singh DhanjalIsmail MunajatTaryn KlarnerIsmini E. PapageorgiouTasnim AhsanIsmini LasithiotakiTat San LauIsrael AmiravTatiana ChristidesIsrael Casado-HernándezTatiana EfimovaIsrael Pérez-TorresTatiana I. ArefievaIssac SachmechiTatiana Maria Suárez-CortésIssam KoleilatTatiana S. KalininaIstván KissTatiana Yu KuznetsovaIstván OsztheimerTatjana DjukicItalo BraghettoTatjana WelzelItamar LoewensteinTatsat PatelItay BarneaTatsuma OkazakiItika GargTatsunori MinamideIulia-Ioana Stanescu-SpinuTatsunori SakamotoIuliana CotiTatsushi MutohIuliana NenuTatsuya FujikawaIuliia A. KorolevaTatsuya ImabayashiIva BinicTatsuya KinIva KiracTatsuya KondoIván Alquisiras-BurgosTatu RojalinIvan BudimirTatyana L. AzhikinaIvan CundrleTauseef AkhtarIvan FistonićTayebeh KazemiIvan GiovanniniTaylor J. ReifIván Herrera-PecoTeacler G. NematadziraIvan L. HandTe-Chun ShenIvan LandiresTeddy WuIvan Lozada-MartínezTe-Feng Arthur ChouIvan MalčićTejabhiram YadavalliIván Martínez-BazTejas BarotIvan PalaginTejas KarhadkarIvan Peres CostaTejeshwar RaoIvan ShirinskyTeleky Bernadette-EmokeIván Valdivia-GandurTeng Grace ZhangIvan VellaTeng-Yu LeeIvana BabovicTeo Jeon ShinIvana HromatkoTeodor M. Svedung WettervikIvana JochmanováTeodora TelecanIvana KraljevicTepetes KonstantinosIvana MravicicTeppei OmoriIvana ŠkrlecTerence A. ImberyIvana StanimirovaTeresa AuguetIvana SutejTeresa C. CardosoIvana TrivicTeresa L. DanforthIveta Y. YotovaTeresa LettiniIvo Dumic-CuleTeresa Makowiec-DąbrowskaIvy Ka Yu ChengTeresa Mary MacDonaldIwahashi NoriakiTeresa PerraIwan August MeynaarTeresa Rubio-TomásIwona Bednarz-MisaTeresa TrenkwalderIwona BonieckaTeresio AvitabileIwona GorczycaTereza GabelićIwona Krela-KaźmierczakTereza NovákováIwona Malinowska-LipieńTeri GreilingIwona PuzioTeri M. KozikIwona Sudol-SzopinskaTerry C. F. YipIwona ŚwiątkiewiczTeru KamogashiraIwona WertelTeruhiko HamanakaIzabela DudaTeruya KomatsuIzabela Maurício De RezendeTessho MaruyamaIzabela ZakrockaTetsuo NishikawaIzabella PawluczykTetsuo ShojiIzumi AsahinaTetsuro TamakiJ. A. P. M. De LaatTetsuya AmanoJ. Andrew OnesimuTetsuya IshikawaJ. Chase McNeilTetsuya OgawaJ. Daniel TwelkerTetsuya TakahashiJ. De MouzonTetsuya ToyonoJ. Domínguez‐CheritTe-Yao HsuJ. J. Van Der SmagtThaddaeus ToenniesJ. Kelly SmithThaíse Gonçalves AraújoJ. Marc SimardThalia MedeirosJ. Pedro TeixeiraThammanard CharernboonJ. S. Hill GastonThanh Thao NguyenJ. W. GuoThanigaivelan KanagasabaiJaap GroothoffThanongchai SiriapisithJacek BilThaschawee ArkachaisriJacek BoguckiThatiane Lopes Valentim Di Paschoale OstolinJacek GagalaThawatchai AkaraviputhJacek MigajThayne KowalskiJacek OwczarekTheddeus Octavianus Hari PrasetyonoJacek PyżalskiTheera TongsongJacek SmerekaTheerut LuangmonkongJacek SzeligaThemistoklis I. DagklisJacek WilczyńskiTheo M. De ReijkeJack AnsellTheocharis G. KonstantinidisJackie Tan-ShowyinTheodomir DusabimanaJackilen ShannonTheodor AdlaJackline Ayres-SilvaTheodor TirilomisJacky Y. SuenTheodora Kunovac KallakJacob E. OllechTheodora PapamitsouJacob E. TeitelbaumTheodore Alexander KungJacob E. TulipanTheodore KarapanayiotidesJacob SkovTheodoros BourasJacobo Rodríguez-SanzTheodoros DimitroulasJacobo Trébol-LópezTheodoros FilippopoulosJacobs AdriaanTheodoros KarapanayiotidesJacobus MullerTheodoros MavridisJacobus PrellerTheofani AntoniouJacobus Van Der VeldenTheoni KanellopoulouJacopo BurrelloTheresa AtkinsonJacopo CiaffiTheresa HauckJacopo FalcoTheresa V. StrongJacopo LisoniTheresa WimberleyJacopo PruccoliTherese BormannJacopo SabbatinelliTherese Fostervold MathisenJacopo VannucciThereza Maria Magalhães MoreiraJacqueline E. ReznikThi Thao Linh NguyenJacques BarbetThibault CanceillJacques MansouratiThibaut ChapronJad Adrian WashifThiemo DingerJae Boum YoumThierno Madjou BahJae Hee ChoThierry BoveJae Ho ChungThierry HauetJae Hoo LeeThierry MomJae Myoung NohThierry SimonartJae Sung LeeThilini N. JayasingheJae Wook YangThilinie RajapakseJae Yon WonThilo WelschJae Yong KimThinzar M. LwinJaehee LeeThinzar MinJaehong AhnThirumal RajJaehun JungThomas A. BucherJae-Hwi NhoThomas A. MastersonJae-Hyeong ParkThomas BartlJae-Hyun KwonThomas Bjørsum-MeyerJae-Hyung ChoThomas ButtgereitJae-Jin KimThomas C. HutchensJae-Joon HwangThomas D’HoogheJaejoon LimThomas DaikelerJaejun LeeThomas DavergneJae-Kwang ShimThomas DenekeJae-Sung YooThomas DietrichJaewon KimThomas E. MayerJaeyul LeeThomas FaitJaeyun WangThomas FinkJafir ShiraziThomas FreimanJagathesh Chandra RajendranThomas HampJagdeep KaurThomas HankJagdish JoshiThomas HegyiJahan AlamThomas HierlJaideep MahendraThomas HollandJaikirshan J. KhatriThomas J. GastJaime O. Herrera-CáceresThomas Jan HeyseJaime Piquero-CasalsThomas KoehlerJaimin PatelThomas LindnerJai-Wun ParkThomas M. HofbauerJakob DörlerThomas M. TiefenboeckJakob GubensekThomas MoumnehJakob MorgensternThomas OchsenkühnJakrin KewcharoenThomas OstermannJakub GlowackiThomas PerreaultJakub Kła̧czThomas Pierre LecompteJakub KlimkiewiczThomas R. KostenJakub KornackiThomas SenonerJakub KristekThomas T. H. WanJakub SzabelskiThomas TannouJamal SaadThomas TischerJameel Rizwana HussaindeenThomas W. Van Der VaartJames BluettThomas W. WainwrightJames C. FordThomas Wesley TempletonJames D. BlankenshipThomas ZellersJames FrictonThoranis ChantraratJames G. HeckerTianfang MaJames GasperinoTianfu WuJames HiltonTianxiao ZhangJames HunterTiberiu Augustin GeorgescuJames Janopaul-NaylorTiberiu BatagaJames LaffertyTiberiu Paul NeaguJames McGuireTibor GyökeresJames MorseTiemin LiuJames P. HunterTien Sy DongJames PowersTieying YinJames R. S. HockerTigestu Alemu DesseJames T. KryzanskiTihomir MustakovJames T. NugentTiina IhalainenJames TsaiTijl VermassenJames V. MichaelTilili BarhoumiJames W. GrauTill Orla KlatteJames WalterTilman CalliessJames WilliamsTim A. FischellJamie BhamraTim A. LudwigJamie K. CapalTim HoremanJamie L. W. KennedyTim HulsenJamir Pitton RissardoTim JodaJamshid Abdul-GhafarTim LynchJamuna Rani AppalasamyTim M. J. EwoldtJan Adriaan GrawTim NessJan BroggerTim PrickettJan C. WüstenfeldTim WalkerJan ChojnackiTim WehnerJan ChrastinaTimo Alexander AuerJan Darius UnterlauftTimo SorsaJan De PooterTimo Z. Nazari-ShaftiJan H. SchaeferTimon VandammeJan HartmannTimothy A. RitzmannJan HenzelTimothy BoergerJan IdkowiakTimothy EricksonJan J. StepanTimothy G. ShortJan Krzysztof NowakTimothy J. MeekerJan KühnischTimothy M. E. DavisJan L. C. LoeffenTimothy PohlmanJán LeštákTimothy PruettJan M. FederspielTimotius Ivan HariyantoJan MersmannTina BartosikJan MirTina FindleyJan NovákTina KapurJan Pauluschke-FröhlichTina TellumJan PetriczkoTina Tičinović KurirJan PřečekTina Ulrike CohnertJan PurruckerTinakon WongpakaranJan Robert KrögerTine L. DohlmannJan SarlonTing ZhongJan SchröderTingchen LuJan ŠkrhaTing-Hsun LanJan TrnaTing-Jung WuJan Van RamshorstTing-Kuo ChangJan Willem KallewaardTingting ShiJan WintrichTingwei ChangJan ZabrzynskiTin-Tin Win-ShweJana HaberlováTirthankar SinhaJana MašlankováTito BrambulloJana NekolováTizian RosenstockJana SvobodováTiziana Di CesareJana TegelbeckersTiziana FeolaJane E. KrauseTiziana GreggiJane SimonsenTiziana PietrangeloJaneen R. JordanTiziana RussoJanet HumeTobi SomervilleJanet S. DufekTobias BerglerJanez RavnikTobias BomholtJani SilvaTobias F. S. PustjensJani T. TikkanenTobias GoeserJanina MarkowskaTobias M. BallhauseJanindra WarusavitarneTobias MettJanmenjoy NayakTobias PuengelJános SinkóTobias RufJan-Paul GundlachTobias SchuppJanusz SielskiTobias WeinbergerJanusz Zenon WójcikTobias WengenmayerJan-Willem BeenakkerTobias ZeusJaques Van HeerdenTodd A. HardyJaques WaisbergTodd MoenJar Yi HoTodd T. SchlegelJared SmithTolga OlmezJared T. AhrendsenTolga ZamanJarlath Christopher BolgerTom C. T. Van RietJarmila SiegelovaTom DecrooJarmila SiegelováTom SunnyJaro KarppinenToma SpirievJaromir AstlTomas BlancoJaroslav LindnerTomas BogielJaroslav MajernikTomas De MayoJaroslav PejchalTomáš KrejčíJaroslav PreslTomas NovotnyJaroslaw ChmielewskiTomas SimurdaJarosław DulskiTomas TamošuitisJaroslaw KwiecienTomaso BottioJaroslaw Meyer-SzaryTomasz BaronJarosław PasekTomasz BlicharskiJarosław TrȩbaczTomasz BochenekJarosław WaloryTomasz BondaJaroslaw ZalewskiTomasz CudejkoJarrad H. Van StanTomasz DziodzioJascha WendelsteinTomasz GabryśJasenka WagnerTomasz GęcaJasmijn V. KorpershoekTomasz GolebiowskiJasmin KnopfTomasz GołębiowskiJasmina IsakovićTomasz GradalskiJasminka ŠtefuljTomasz GrzelaJasna JančićTomasz HirnleJasna KusturicaTomasz JadczykJason AckrivoTomasz KluzJason AcworthTomasz KobielaJason Daniel WagganerTomasz M. KarpinskiJason José BendezúTomasz MazurekJason Philip AppletonTomasz RakowskiJason QuTomasz RusakJason S. ChinitzTomasz StompórJason S. RadowskyTomasz TokarekJason SuterTomasz TrzmielJason TasoulasTomasz UrbanowiczJason WashburnTomasz W. KaminskiJason X. ChengTomasz ZarnowskiJason YapTomasz ZielinskiJason YuenTomaszewski MichałJasper QuakTomaž GoslarJasper StevensTomaž GračnerJasper VergutsTomaž ŠtupnikJaspreet KaurTomaž VovkJaume MarrugatTomer Ziv-BaranJaume Miranda-RiusTomislav BokunJavad AlizargarTomislav KizivatJavad HeshmatiTomislav KokicJavad Javan-NoughabiTomislav KuzmanJavier ChinenTomislav RukavinaJavier CoboTommaso B. JanniniJavier De La CruzTommaso Di MairaJavier FernándezTommaso FossaliJavier García-AbellánTommaso Francesco GalbiatiJavier GómezTommaso GrecoJavier J. García-AlegríaTommaso NeriJavier Jiménez-CandilTommaso PettenuzzoJavier LacorzanaTommaso SteccaJavier LumbrerasTommaso VerdolottiJavier MendicuteTomohiko AoeJavier Menéndez-MenéndezTomohiko NishigamiJavier ModregoTomohiko SadaokaJavier Molina-CerrilloTomohiro OsakiJavier OsorioTomohiro UnoJavier Quillo-OlveraTomohiro YazawaJavier SantosTomokazu ShimizuJavier-David Lopez-MorinigoTomoki MaedaJawad KhanTomoki NakamuraJay YadavTomoko AokiJaya Shanker TedlaTomoko KuritaJayabalan NirmalTomomi KotaniJayalakshmi VallamkonduTomonori KenmokuJayanta SamantaTomotaka KawayamaJayanthi ParthasarathyTomotaka SaitoJayson StoffmanTomotaka UgaiJe Hyeok OhTomoya FujieJe Hyun SeoTomoya HiroseJe Hyung HwangTomoya TateishiJeadran Malagón-RojasTomoya YokotaJean AmiralTomoyuki HiokiJean Benoît CorcuffTomoyuki KanazawaJean Christophe IanottoTomoyuki SasanoJean De La RosetteTomoyuki TakigawaJean François CailhierTomoyuki Tomoyuki AsadaJean François LlitjosToni P. MilesJean GuibourdencheTonking BastolaJean KanitakisToon DuijnhouwerJean M. DavidsonTope OyeladeJean Marc AyoubiTorben GlatzJean Marc LévêqueTorbjörn LedinJean Paul M. ValléeTöres TheorellJean Paul Semasaka SengomaTorgny SunnerhagenJean Philippe MironToril FjørtoftJean Pierre LindenmayerTorsten ChristJean-Baptiste RiccoTorsten LoopJean-Christophe LegaTorsten PastorJean-Christophe PhilipsToru AwayaJeané CloeteToru KotaniJean-François ChenotToru MiwaJean-François DeuxToru TaguchiJean-François SarrazinToru WakamatsuJean-François SubraToru YagoJean-Francois TimsitToshiaki IbaJeanine R. Jarnes-UtzToshiaki KotaniJean-Marie BardToshiharu FujiiJean-Michel LavoieToshihiko MatsumotoJean-Michel LeméeToshihiro FukuiJeannette SchenkToshihiro KawaeJean-Philippe BerteauToshihiro MurajiJean-Philippe DidonToshihiro TsurudaJean-Pierre GagnerToshikatsu TanakaJean-Pierre HoToshiko NakaiJean-Rémi LavillegrandToshimitsu KatoJeanson ArnaudToshimitsu YamaokaJean-Stephane DavidToshinobu KazuiJean-Xavier MazoitToshiyasu NakamuraJean-Yves HogrelToshiyuki KawaiJee Hye WeeToshiyuki MatsuiJee Hyun KimTouraj EhtezaziJee Taek KimToyonori TsuzukiJeena GuptaTracy A. WilliamJeewan ThapaTravis B. NielsenJeff C. W. WangTrevor GersonJeffery L. MeierTrifan Anca-VictoritaJeffrey B. VelottaTrim LajqiJeffrey Che-Hung TsaiTrine KåsineJeffrey D. DellaVolpeTrinidad Montero-VilchezJeffrey E. IndesTrisha SinghJeffrey E. JanisTrisha V. VigneswaranJeffrey KahnTristan De NattesJeffrey M. Dodd-OTristan T. HormelJeffrey R. BorisTrond Geir JenssenJeffrey S. ForsseTruls E. Bjerklund JohansenJeffrey SchweitzerTsan-Chi ChenJeffrey SilberzweigTse-Shao ChangJeffrey VanWormerTso-Chou LinJeffrey W. BellTsong Huey ChewJegan IyyathuraiTso-Ting LaiJehan AlamTsukasa YoshidaJelena KovacTsunahiko HiranoJelena Marković FilipovićTsung Li LinJelena MirnićTsung-Hsien LeeJelena MunjasTsung-Hsuan LaiJelena S. DrulovićTsung-Jen WangJelena StojanovićTsung-Jung LiangJelena VekicTsung-Li LinJelena VeličkovićTsung-Yu HuangJelica Grujić-MilanovićTsutomu NakashimaJelle OverboschTsuyoshi IchinoseJen-Chih TsengTsvetelina BatsalovaJeng-Dau TsaiTsvetelina VelikovaJeng-Ren DuannTsz Wing LeungJeng-Wen ChenTuck YongJenica N. UpshawTudor Claudiu SpînuJeniffer CarrilloTudor Sorin PopJennifer A. ZellersTuğçe Aksu UzunhanJennifer B. DennisonTu-Hsuan ChangJennifer BeldingTunc ErenJennifer C. PisanoTunde PetoJennifer FangTurgut B. CengizJennifer FredianiTuvia Ben GalJennifer J. McComasTuvia Ben-GalJennifer L. EatonTyler A. ScullenJennifer M. BinghamTymoteusz MillerJennifer M. EatridesTyng Yuan JangJennifer M. LynchTyrone PretoriusJennifer M. VojtechTzer-Bin LinJennifer MytychTzu-Chieh ChouJennifer ThorneTzu-Ting HuangJennifer Van WykTzu-Yen HuangJennifer ZitserTzu-Yu PengJenn-Ming YangTzyy-Ling ChuangJenny BischoffUberto BortolottiJenny HoitUday Praful KundapJenq-Shyong ChanUdhayakumar RamalingamJens BarthelmesUgo CovaniJens J. RassweilerUgo De GraziaJens Kjeldsen-KraghUgo GrossiJens KöllermannUgo IndraccoloJens Steen NielsenUgo LimbrunoJeong DongtakUgo MarcheseJeong-Ho SeokUgolino LiviJeong-Hoon LimUiju ChoJeongkui KuUlderico FreoJeong-Kyu LeeUlf GunnarssonJeremias Bayon LorenzoUlf GyllenstenJeremy AugustinUlf SchönermarckJérémy BourenneUliana A. TsoyJeremy CharriotUlrich LienerJérémy CorrigerUlrich RotherJeremy D. P. BlandUlrich Severin DischingerJeremy J. PomeroyUlrich SpeckJeremy RempelUlrich WalterJeremy S. LeventhalUlrike KuchlerJer-Ming ChangUlrike KumpfJeroen C. HolUlrike M. M. BauerJeroen Van SchependomUlrike RaapJerome AvouacUlrike Van DaeleJerome CanadyUlrikke VossJér̂ome René LechienUmar HayatJérôme RollinUmberto AgugliaJerome TannerUmberto BaccaraniJerónimo Aragón VelaUmberto BracaleJerónimo González-BernalUmberto CommitteriJerred Junqi WangUmberto LorenziJerremy WeertsUmberto MalapelleJerritta SelvarajUmberto TarantinoJerry Y. DuUmer SaeedJer-Yen YangUmesh Chandra BeheraJerzy BiałeckiUmesh VenkatesanJerzy ChudekUmut CelikyurtJerzy JankauUna D. NedeljkovićJerzy MackiewiczÜnal BıçakcıJerzy MosiewiczUndefined ShalimarJerzy WisniewskiUndral MunkhsaikhanJeske WalterUp HuhJesko L. VerheyUpasana RayJess D. EdisonUri FarkashJess LambrechtsenUrmi KhannaJesse GroenUrna KansakarJesse HatgisUros BumbasirevicJesse JupiterUrska ArnautovskaJessica BriffaUrsula CatenaJéssica Conti RéusUrsula ReiterJessica CusatoUrsula WernekeJessica GonzálezUsha KimJessica H. HustonUtkarsh KohliJessica J. HarrisonUwe PlatzJessica M. CottreauUwe Von FritschenJessica Magid-BernsteinV RameshJessica PistellaV. E. OleynikovJessica RademacherV. G. AbilashJessica RanieriVadim ByvaltsevJessica S. WestVadim V. GenkelJessica S. WhittleVahid EslamiJesús Abelardo Barea-MendozaVahid KhosravaniJesus AibarVahid ShaygannejadJesús García-CanoVaidotas UrbonasJesus S. JimenezVaishali AggarwalJesus Salvador Jimenez LopezValdis PīrāgsJeyasakthy SaniasiayaValentina Brzović RajicJezid MirandaValentina BucciarelliJi Bak KimValentina CossigaJi Hwan ParkValentina De NunzioJi Hyun KimValentina GattiJi Hyun ParkValentina GiardiniJi YangValentina GrazioliJi Yih ChenValentina IoriJi Young AhnValentina IzzoJiaan-Der WangValentina Lucia La RosaJia-Horung HungValentina LupatoJian GeValentina OpancinaJian WangValentina ScanoJianan ZhaoValentina TostoJianguo SunValentina TsibizovaJian-Jun LiValentina ValleJianli NiuValentina VicennatiJiann-Her LinValeria AlbanoJianqiu KongValeria AllizondJiantao YaoValeria BelleudiJianwen FangValeria BrazzelliJianxiang XueValeria CorradettiJianyuan ChaiValeria DionisiJiaojiao ChengValeria GambacortaJiaqi ZhangValeria GiorgiJiawei LiuValeria MussoJiawei WangValeria StatiJiayan PanValeria TombiniJia-Yih FengValeria ViscoJichang WangValérie BillardJie ChenValerie Bracchi-RicardJie DingValérie McLinJie JiaValerio BonavolontaJie LiuValerio GallottaJie SunValerio LeoniJie ZhuValerio MaisJiejie LiValerio SanguigniJien Jiun ChenValeriu ArdeleanuJien-Jiun ChenValeriu Marin ȘurlinJiheum PaekValery V. LikhvantsevJijun HuangValeryia MikalayevaJill AdamskiValeska HofmannJillian R. RichterValter TravagliJiman ParkVamsee MallajosyulaJin BaiVan Giau VoJin ČhengVanesa Antón-VázquezJin Joo ChaVanesa BlázquezJin Wook KimVanessa Barreto RochaJin Yong JeongVanessa BianconiJin YuanVanessa FreyJin ZhangVanessa SciaccaJinae LeeVan-Giau VoJinan LiVanna Chiarion-SileniJing CaoVarah YuenyongviwatJing YuanVarda Gross-TsurJing-Jing FangVarius DannenbergJingjing HuangVartika TomarJingjing SuVarun GuptaJingwen CaiVarun MongaJingyu WangVasile ForisJin-Heon JeongVasile PotopJinhong MinVasileios SiokasJin-Hwa JungVasileios VavourakisJin-Hyuck LeeVasilena SitnilskaJin-Luan LiVasilije StambolijaJinming HanVasiliki ChantziaraJinna YaoVasiliki KaioglouJinn-Rung KuoVasiliki RaptiJin-Seng LinVasiliki TsigkouJin-Tung LiangVasiliki VlachaJinwei WangVasilios E. PapaioannouJinwei XieVasilios PergialiotisJirada SringeanVaska Vandevska-RadunovichJiří BonaventuraVassilis L. TzounakasJiří KarásekVassilis SkampardonisJiri MálekVasthi LópezJiri PlasekVasudev BallalJitender DabasVasudeva IyerJitendra KanshanaVedat EljeziJitendtra Kumar Singh PariharVedat TopsakalJitin BajajVedran PremužićJitka VeldemaVeeranna MaddipatiJiuann-Huey Ivy LinVeerapong VattanavanitJiucun WangVeerappan R. MuthukkaruppanJiunn-Liang ChenVelayutham RavichandiranJiun-Wen GuoVeljko PetrovićJiwan GeVendel KristensenJiwang ZhangVenija CeroveckiJi-Won KwonVenkatraman GaneshJiwon RyuVenketesh SivaramakrishnanJiyun LeeVera A. YakupovaJo KatoVera BékésJo Murphy-LawlessVera LarinaJo NijsVerena BoßungJo RaskinVerena EllerkampJoachim KunzVeria VacchianoJoachim RisseVerica PavlicJoan BagóVern Hsen TanJoan CalvetVeronica AbateJoan Espaulella-PanicotVeronica AranJoan GilVeronica BaioccatoJoan Jiménez-BaladoVeronica De SimoneJoan Leal-BlanquetVerónica García-SanzJoana F. SacramentoVeronica Mugarab SamediJoana Oliveira MirandaVeronica OjettiJoana PereiraVerónica Romero-FerreiroJoanna A. MusialikVeronika HufJo-Anna B. BaxterVeronika OlejnickovaJoanna BartosinskaVesna Mijatovic-JovinJoanna ChorbińskaVesna TesicJoanna CzerwińskaViann Nguyen-FengJoanna Domagala-KulawikVibha TandonJoanna E. ŚliwkaVibhu Krishnan ViswanathanJoanna FilipowskaVibor MilunovićJoanna GołębiewskaVicenç FalcóJoanna Gromadzka-OstrowskaVicenç Torrente-SegarraJoanna JaniszewskaVicent Modesto I. AlapontJoanna JaworskaVicent Primo RomagueraJoanna Kacperczyk-BartnikVicenta Llorente-CortesJoanna KapustaVicente Bertomeu-GonzálezJoanna KonopińskaVicente Calixto Zanón-MorenoJoanna KowalskaVicente ClimentJoanna Maria LotowskaVicente Faus-MatosesJoanna Myszkowska-RyciakVicente Pallarés-CarrataláJoanna Perła-KajánVicki PattonJoanna Roskal-WałekVicki Trier TaastiJoanna TrelinskaVicky EhlersJoanna Wiktoria Przeździecka-DołykVicky Panduro-CorreaJoanna Wojtasik-BakalarzVictor Doménech GarcíaJoanne FieldingVíctor Gómez‐MayordomoJoanne MurrayVictor Jimenez-YusteJoanne YipVictor LlorençJoao A. A. SantinhaVíctor M. RiveraJoão Agostinho Machado-NetoVictor MarkJoão Antônio Leal De MirandaVictor MartinJoão Azevedo-SilvaVictor PalarieJoao Barbosa-BredaVictor R. GordeukJoao BotelhoVictor U. SchmidbauerJoão BotelhoVictor ValderrabanoJoão CaramêsVictor Vlad CostanJoão De Melo NetoVictoria GaribJoão Gama MarquesVictoria Gómez-Dos-SantosJoão Gaspar-MarquesVictoria M. LeavittJoão Gualberto De Cerqueira LuzVictoria MacBeanJoão M. SantosVictoria Mazoteras-PardoJoão Manoel Silva JúniorVictoria S. McKennaJoão Manuel Mendes CaramesVictoria Therese MückeJoão MartinsVictoriano GarreJoão Miguel Marques Dos SantosVictor-Vlad CostanJoāo Nobre CardosoVid MatišićJoão Paulo Mandes TribstVida DemarinJoão Sequeira AlvesViet LeJoão-Paulo-Mendes TribstViet TranJoaquim Murray Bustorff-SilvaVijay Kumar JidigamJoaquim Soares Do BritoVijay LetchumanJoaquín PortillaVijaya Lakshmi NagJochen KaiserVijaya SarathiJochen ReiserVijayakumar SukumaranJodi Ann EdwardsVik KhullarJody Corey-BloomVikas SharmaJoel GelmanVikas TandonJoel PolletVikash JaiswalJoelle KarlikVikash KansalJoerg KrebsVikram ThakurJoerg-Ingolf SteinVikrant RaiJoeri Jan PenViktor ChrobokJoesph RafettoViktor SimeonovskiJohan G. J. Vande WalleViktoria MusterJohan HambraeusViktória WoronikJohan HoebekeVilai KuptniratsaikulJohan NobleVilim MolnarJohan RaederVilma PinchiJohann HenckelVimal ChadhaJohann SellnerVimal MehtaJohanna TakácsVinayak M. JoshiJohanna WestraVinayKumar HallurJohanne Le Beyec-Le BihanVincent AntoJohannes BonattiVincent Blasco-BaqueJohannes CarlVincent CastelainJohannes EhlerVincent J. AlentadoJohannes H. SchmitzVincent JavaugueJohannes HoferVincent L. RoweJohannes J. RingVincent Peter C. MagbooJohannes KerstenVincent YeungJohannes M. AlbesVincente Javier Clemente-SuarezJohannes MagerVincenzo AbbateJohannes Maximilian AlbesVincenzo CandelaJohannes OppermannVincenzo CastiglioneJohannes OttVincenzo CataneseJóhannes ReynissonVincenzo De FrancescoJohannes Schneider-ThomaVincenzo FiorentinoJohannes StoecklVincenzo Giannicola MendittoJohannes WachVincenzo La MuraJohari Yap AbdullahVincenzo Livio MalavasiJohn A. CaldwellVincenzo NuzziJohn A. D’EliaVincenzo PatellaJohn D. FitzgeraldVincenzo PolizziJohn D. HorowitzVincenzo RicciJohn Dirk Vestergaard NielandVincenzo SavarinoJohn DotisVincenzo SucatoJohn EismanVincenzo VizziJohn ElefteriadesVincenzo VoiJohn EllulVineet GauharJohn F. WardVineet K. RaghuJohn G. QuigleyVineeta TanwarJohn H. DarkVinod KumarJohn H. PyneVinodh KakkasseryJohn HarrisonViola Maria PopovJohn HortonViola SangaJohn J. NemunaitisVioleta AlarcãoJohn K. YueVioleta MonasterioJohn KellumVirginia CipolliniJohn L. ReaganVirginia CorazziJohn M. BarryVirginia Sánchez-MonroyJohn M. CarneyVirginie Ève LvovschiJohn M. FlackVirginie LemialeJohn M. PerryVisanee TantiseviJohn N. AllanVisasiri TantrakulJohn R. RinkerVishakha Anand PawarJohn RiceVishal ChavdaJohn RudanVishal Vishnu TewariJohn S. WienerVishma Pratap SurJohn T. SchulzVishnu Muthuraj KumarasamyJohn T. SigalosVishnunarayan Girishan PrabhuJohnathan Ross RenewVishwajeet SinghJohn-David AubertVissaria SakkaJolanta CylwikViswas Raja SolomonJolanta DadonieneVitali FrancescoJolanta DadonienėVitaliano F. MuziiJolanta E. LosterVito PesceJolanta Justina VaškelytėVito RomanoJolanta Klukowska-RötzlerVitolds MackevicsJolanta Kostrzewa-JanickaVitor Barreto ParavidinoJolanta Kucharska-MazurVítor CavadasJolanta Nawrocka-RutkowskaVittorio AprileJolanta Parada-TurskaVittorio DibelloJolanta StrzeleckaVittorio Emanuele BianchiJolien RobijnsVittorio FaveroJon Ander Nieto-GaraiVittorio FuscoJon B. SuzukiVittorio MazzarelloJon OdlandVittorio RamellaJon P. WietholterVittorio SambriJon Salmanton-GarcíaVivek Kumar SinghJon StavresVivek PandeyJon WadsleyVivek PatelJonas A. PramuditaVivek PecheJonas Michel WolfVivek SinghJonas Müller-HübenthalVivek YedavalliJonatan García CamposVivian F. KaulJonatan OrasVivian Paraskevi DouglasJonathan BarrattViviana IvanJonathan BusadaViviana MucciJonathan C. P. RoosViviana OnofreiJonathan D. HoViviane A. SarmentoJonathan D. KochavVlad AlexandrescuJonathan DavidVlad CiobotaruJonathan FordVlad GorduzaJonathan GreenbergVlad PădureanuJonathan H. PelletierVladiana TuriJonathan HowlandVladimir A. ShvartzJonathan J. H. BrayVladimir BedekovićJonathan LecureuxVladimir HeiskanenJonathan LuisiVladimir KenisJonathan Michael FlanaganVladimir LozanovskiJonathan P. GiurintanoVladimir M. ShipulinJonathan RoydsVladimir PorochJonathan SaxeVladimir ShvartzJonathan SleemanVladimír ŠrámekJonathan TownendVladimir TesarJonathan WislerVladimir TodorovJonathon R. HowlettVladimir V. BezuglovJone A. StanleyVladimir VekslerJong Hun KimVladimir YussupovJong Yeon LeeVlad-Iustin TicaJongbin KimVlad-Olimpiu ButiurcaJonghwa JangVlasios KarageorgosJong-Jer LeeVlatka LajnertJong-Ki HuhVlatka ReskovicJongkil JooVo Truong Nhu NgocJongsang SonVojkan LazićJong-Woon ChoiVojko KanicJonhatan SilvaVojtěch PeřinaJoo Hee YoonVolkan ArısanJoo Hyun OhVyacheslav OgayJoon Hyop LeeW. Bijlsma MerijnJoon Kee LeeW. Tony ParksJoon Y. KangWade BerritinniJoongbum ChoWadi N. SukiJoost M. MeijerWael AhmadJoost Van Der VorstWael SaadeJoost Van MelleWagih Mommtaz GhnnamJordan HalseyWahyu CaesarendraJordan P. SandWai LeeJordi BoverWajdy AlawaidaJörg FelderWaldemar GozdzikJörg HänzeWaleed MohamedJörg KempfertWalid AlrayashiJorge A. Roman-BlasWalid Mahmoud KhaliliaJorge AramburuWalid ShalataJorge C. KattahWally WojciechowskiJorge Calderón-ParraWalter ColangeliJorge CastanonWalter DorigoJorge Francisco Gómez CerezoWalter E. KaufmannJorge GrasaWalter FlapperJorge Gutiérrez-CuevasWalter FurlanJorge MartinsWalter J. FlapperJorge N. R. MartinsWalter L. StrohmaierJorge PetroWalter LischJorge Piano SimõesWalter M. Van Den BerghJorge Sánchez InfanteWalter Roman-JuniorJorge Santos-JuanesWalter SerraJorgen BauwensWan Aliaa Wan SulaimanJorinde A. W. PoldermanWan Nordiana RahmanJörn SchmittWanchun SuJosanna M. Rodriguez-LopezWanda K. O’NealJoscha BuechWang Zheng-XinJosé A. CarriónWanguo LiuJosé A. Gómez-PuertaWanjiku MathengeJose A. GuirolaWan-Jin ChenJose A. O. SimoesWan-Ju Annabelle LeeJosé A. RizzoWan-Long ChuangJosé Alexandre ReisWanni LinJosé Ángel CabreraWan-Tai M. Au-YeungJose Antonio Alvarado-MorenoWanwen ZengJosé Antonio García-ErceWaqas A. BurneyJose Antonio Lopez-EscamezWarawut ChaiwongJosé Antonio Morales-GonzálezWasan KatipJosé Antonio RianchoWasim S. El NekidyJosé Avendaño-OrtizWassan NoriJose Barreto CarvalheiraWasundara FernandoJosé Carlos CarvalhoWataru AndoJosé De La Flor MerinoWataru IsonoJose Donato Jr.Wei ChengJosé E. KriegerWei Cheong NgeowJosé Eduardo TeixeiraWei ChiJosé EleutérioWei Dong GaoJose Francisco Tornero-AguileraWei Hsum YapJose G. MantillaWei HuangJose Garcia RodriguezWei Shiang LinJosé Gerardo González-GonzálezWei SuJose Gomez-SanchezWei Wen LimJose IglesiasWei WuJose Ignacio BilbaoWei ZhaoJose Ignacio Salmeron EscobarWei-Chieh HuangJose Jair Alves Mendes JuniorWei-Chieh LeeJosé López-LópezWei-Chih ChenJosé Luis Cebríán-CarreteroWei-Chun LinJosé Luis Clúa EspunyWei-Chyou WeiJosé Luis Martín-ContyWeifeng ZengJosé Luis Muñoz De NovaWei-Hsun YangJosé Luis Rodríguez GutiérrezWeihua DingJosé Luís Sánchez-CarazoWei-Jen TingJosé Luis Vázquez NogueraWeiming OuyangJose M. GamonalesWeisyun HuJose M. MestreWeita ChenJose M. RamirezWeiwei CuiJosé Manuel CifriánWei-Yi WuJosé Manuel MendesWeiyun ShiJosé Manuel RubínWellington P. MartinsJosé María De Miguel-YanesWellington Pinheiro Dos SantosJosé Maria LassoWen Hai ShaoJose Maria MarimonWen Huei LiaoJosé María Mora-GutiérrezWen Lin ChaiJose Maria Pereira De GodoyWen-Bin WuJose Medina PoloWen-Bo ZhangJosé Miguel González-ClementeWen-Chih LiuJosé Miguel Seguí-RipollWen-Chun ChangJose Miguel Sempere OrtellsWenchun ChenJose N. Sancho-ChustWendy Scholten-PeetersJose PerdomoWenfei ZhuJose Sebastián Velázquez BlázquezWen-Fu LaiJose TunonWenhui XieJosé Ursic-BedoyaWenjing ZhangJosé Valentín García GutiérrezWen‐Kuan ChiuJosé Vicente Andrés PeiróWen-Pin HuJosé Vicente Noronha SpolidoroWenting GuoJose ZeballosWen-Wei SungJose-Antonio Mirón-CaneloWenxin SongJosef BaumgartnerWenyan ZhangJosef FinstererWen-Yih JengJosef Georg GrohsWenzel M. HackengJosef SmolleWenzhen CaoJosefa Gonzalez-SantosWenzhen GeJosefine RotheWerner GrafJosé-Juan Pereyra-RodriguezWerner KrutschJose-Manuel Ramos-RinconWerner StreifJosé-María Sánchez-GonzálezWeslania Viviane NascimentoJosep Maria UstrellWeston T. PowellJosep Pardos-GeaWey Ran LinJoseph Boone MuhlesteinWibke Müller-SeubertJoseph FurakWiebke SolaßJoseph J. CrivelliWiebke SommerJoseph KauerWieland ElgerJoseph M. RutkowskiWieslawa DuszynskaJoseph MaaloulyWiesława MłodawskaJoseph MagagnoliWijnand K. Den DekkerJoseph NassifWilco ZijlmansJoseph P. H. McNamaraWilhelm BehringerJoseph PikkelWilhelm EisnerJoseph SerroneWilhelm MistiaenJoseph T. ChurchWillaert EvaJosephina SampsonWilliam ChanJosé-Tomás NavarroWilliam D. Moscoso-BarreraJosh SchroederWilliam E. AckermanJoshua A. BloomWilliam G. BlakeneyJoshua C. DoloffWilliam HurfordJoshua D. WhittakerWilliam KongJoshua HeflerWilliam KraemerJoshua I. RosenbloomWilliam MacAllisterJoshua J. X. LiWilliam Mark ErwinJoshua M. BockWilliam P. HausdorffJoshua RezkallaWilliam PoncinJoshuan J. BarbozaWilliam PrimackJosip A. BorovacWilliam R. MowerJosip TomićWilliam RomanJosko BozicWilliam W. StringerJosue MartosWilliam Y. RaynorJosy AugustineWilliams RiccardoJothi VargheseWilly HauzerJo-Ting TsaiWilmer Gianfranco Silva-CasoJouni LauronenWindy McNerneyJovana BjekićWinfried M. K. AmoakuJoyce PopoolaWing Chung ChangJoyce TengWing Lok ChanJoyce WhittingtonWinnie C. W. ChuJozef BudayWioleta KowalskaJozsef BallaWioletta PawlukowskaJózsef LaczkóWira WinardiJózsef TollárWitold GertychJu Ok ParkWitold PolanskiJuan Antonio Valera-CaleroWitold StrebJuan Bautista De SanctisWitold TłustochowiczJuan Bosco López-SáezWitold Z. TomkowskiJuan Bueno-NotivolWlodzimierz SamborskiJuan Carlos Falcón-GonzálezWojciech CytawaJuan Carlos Garcia-PaganWojciech DąbrowskiJuan Carlos GrignolaWojciech DudekJuan Carlos Martinez EspinosaWojciech EliaszJuan De Dios Benítez SilleroWojciech GlinkowskiJuan Esteban Gómez-MesaWojciech JachećJuan F. BlancoWojciech KiebzakJuan Francisco Rodríguez-TestalWojciech KlocJuan Francisco Vazquez CostaWojciech MagońJuan GallardoWojciech PepkeJuan Garcías-LadariaWojciech SatoraJuan Garduño-EspinosaWojciech SkrobotJuan Gerardo Reyes-GarcíaWojciech WańhaJuan Gómez RivasWojciech WitkiewiczJuan J. BadimonWojciech WolanśkiJuan Jose AcevedoWolf DietrichJuan José Criado-AlvarezWolfgang A. WetschJuan José LarrañagaWolfgang F. BuhreJuan José López-NúñezWolfgang FitzJuan Jose Nieto FontarigoWolfgang Hohenforst-SchmidtJuan Luís Chávez-PachecoWolfgang KernerJuan Luis Leon-LlamasWolfgang RascherJuan Luis Muñoz BellidoWolfgang SchadenJuan Luis Sánchez GonzálezWollborn JakobJuan M. Hincapie-CastilloWon Young KimJuan Manuel Espinosa-SanchezWon-Bae ParkJuan Manuel Marquez-RomeroWong AdrianJuan Manuel PerezWonhyo SeoJuan Martin PalomoWonjung HwangJuan Maza-SolanoWon-Keun KimJuan Miguel Martínez-GalianoWon-Pyo LeeJuan Mozas-MorenoWoo June ChoiJuan Pablo Sanabria-MazoWoohyeun KimJuan PalomoWoojae HanJuan Patricio NogueiraWoo-Jong KimJuan Posadas-CallejaWoong Jin BaeJuan QinWoonhyeok JeongJuan Rodríguez MansillaWooyeong ParkJuan TorrasWorakan PromphanJuan Torres-MachoWouter KokJuan Vicente MampelWu YaoJuana María Bretón-LópezWulf SchneiderJuan-Manuel AnayaWyss FernandoJubina VenghateriXanthopoulos KonstantinosJudit Cabana-DomínguezXavier A. SantanderJudith HoningXavier DrouotJudith KarpXavier HeimJudith SchwartzbaumXavier VallèsJudith WienkeXianchen LiuJudy Chia-Chun YuanXiang QianJudy M. Muller-DelpXiang WangJudy OuXiang ZhouJuei-Tang ChengXiangchen DaiJuergen GrafenederXiang-Guo LiJuergen HoneggerXiangjiu DingJuha TaavelaXiangli ZhaoJuhan LeeXiangpeng KongJuhani AiraksinenXiangpeng LiaoJuhani H. MäättäXiangsheng FuJuhra ChristianXiangyu ZhangJui-Chieh ChenXianqi LiJui-Sheng SunXianwen WangJukka KanervaXianxiu ChenJukka KoffertXianyi ZhangJukka P. KoffertXiao XuJukka YlikoskiXiaodong GeJulajak LimsrivilaiXiao-Dong ZhouJules MesnierXiaofei LiJules Tianyu Zhang-YinXiaoguang LiuJulia ChisholmXiaohai MaJulia DlugaiczykXiaohan FanJulia FerrariXiaohui Rausch-FanJulia K. BosdouXiaohui ZhangJulia L. M. DunnXiaojiong JiaJulia M. PotterXiaokang WuJulia PrillerXiaoliang GanJulia RadosaXiaoming DuJulia Sarah Maria ZimmermannXiaonan ZhangJulia SarantXiaoqiang TangJulia SongXiaoyan DuJulian KünzelXichi WangJulián L. Zayas-ArrabaXijing WangJulian SchererXin CuiJulian SingerXin TongJulian VeserXin WangJulian WolfesXin YeJuliana FernandesXinchen WuJuliana HackXingchun GaoJulie BolcaenXinghua WangJulie Brogaard LarsenXinglong ZhengJulie Chauvel-PicardXingping QinJulie CwikelXingshun QiJulie HagstrømXingyu WuJulie K. FreedXin-Hua XiaoJulie PerinelXinlei LiJulie ShulmanXinli DuJulie SoulardXinni SongJulie SwartzendruberXintao TuJulie VrankenXinyue DongJulie WangXiucong BaoJulieanne P. SeesXiuhua WengJulien AncelXiulan YangJulien BerhouetXiying GuanJulien CarvelliXizhen XuJulina Md-NoorXóchitl Ortiz-JiménezJulio Cesar AguilarXóchitl TrujilloJulio Cesar FranciscoXosé Luis Pérez-FernándezJulio Francisco Jiménez-BonillaXu ChenJulio Ortega-UsobiagaXuan LiJulio Plaza DíazXuan LiuJulio VillanuevaXuan ZhangJulius HöhneXuanang XuJuliusz HuberXucheng HouJun HiroseXuejuan JiangJun Hui JeongXuenong ZouJun KishiharaXue-Song LiuJun Neng RoanXuhua TanJun OtoXuming MaoJun WanXurxo Dopico CalvoJun WangYacine JaïdiJun YamashitaYacine OuahchiJun YuYacov ShachamJunaid AhmedYaddanapudi RavindranathJunaidi KhotibYadong HuangJuneo Freitas SilvaYaei TogawaJunfei ZhaoYael ZaltzJunfeng WangYafei WangJung Suk LeeYafeng LiJung-Hee LeeYahya AhmadipourJung-Ho YunYahya SefidbakhtJungmyung LeeYaichiro OkuzuJungtae AhnYair BenyaminJunhyung KimYamei TangJunichi IwataYamini SubramaniJunichi KikutaYan HuangJunichi ShimizuYana Roka-MoiiaJun-Jen LiuYancy FerrerJunli ZhaoYanfeng GaoJunling ZhuangYang ChenJunmei ZhangYang PengJunnliang ChangYang YangJunsheng PengYangpeiwei HuangJunya ItoYangtian YiJunya SatoYangyang ZhangJunyoung AhnYangyiran XieJun-Young YangYanhe LueJunzi WuYanis Toledano-MagañaJuraci A. CesarYanjie ZhangJuraj PayerYanling YanJuraj PrejacYannick Fabian DiehmJure AljinovicYannick Van SleenJürgen GermannYannik KalbasJürgen PannekYannis K. ValtisJurgen SchleefYannis TheodorakisJuri TaborriYanwu XuJurij HanzelYanxia LuJurij Matija KališnikYao WangJuš KšelaYaodong GuJu-Seop KangYaolung ChangJussi KosolaYao-Te TsaiJustas ZilinskasYaron Bar-LavieJustin A. YuYasar Mehmood YousafzaiJustin D. KrogueYash GuptaJustin FerdinandusYash Paul SharmaJustin ParkYashbir SinghJustin W. SilversteinYashima KazuoJustus BaumgartenYashuhiko EbinaJustus G. GarwegYasin YilmazJustus GroenYasir RehmanJustyna AgierYasir WaheedJustyna Cwajda-BiałasikYasmin MaorJustyna Gornowicz-PorowskaYasmyne C. RonquilloJustyna KowalskaYassir A. YassirJustyna KutybaYasuhiko BabaJustyna Małyszek-TumidajewiczYasuhiko HayashiJustyna MiszczykYasuhiko SugawaraJustyna Opydo-SzymaczekYasuhiro IzumiyaJustyna SikoraYasuhiro KanataniJutta ArensYasuhiro MochidaJu-Yi ChenYasuhiro OkumuraJuzer ShabbirYasuhiro TakahashiJwalant MehtaYasuhiro YokoyamaJyh-Miin LinYasuka MatsunagaJy-Ming ChiangYasuko FujitaJyoti Bala KaushalYasukochi SatoshiJyoti UpadhyayYasunori IidaK. A. PritchardYasuo ItoK. N. ChethanYasushi IshigakiK. R. RamanathanYasushi KotaniKaboni Whitney GondweYasushi ShimadaKadri SuijaYasushi ShinoharaKai GuoYasushi UekiKai ZacharowskiYasushige ShinguKaia LaidraYasutaka KakinokiKai-Che WeiYasuto KinoseKai-Chun ChengYasutoshi KimuraKaijian HouYasutsugu ShionoKailash NarayanYasuyuki ShiraishiKailash SinghYau Kei ChanKaixin LiangYavuz Emre ŞükürKaja KasarełłoYaw Duah BoakyeKajal KhodamoradiYawei J. YangKajo BućanYawei LiKalaimani ElangoYaying SunKalathil K. SureshkumarYazine MahjoubKaleen HayesYazmín Hernández-DíazKa-Leung WongYe CaoKalliopi AlpantakiYeChun RuanKalliopi PlatoniYegor TryliskyyKalyanasundaram MadhuYehia SalehKamajit SinghYehong ZhuoKamal El BissatiYen Po WangKamal KishoreYen-Chun PengKamalesh ChakravartyYen-Nung LinKamayani SinghYen-Wen ShenKameel KassabYeo Jin KimKamen V. VlassakovYeon Hyeon ChoeKamil GillYeunjoo E. SongKamil MichalikYevgeniy BrailovskyKamil NelkeYezaz GhouriKamil WdowiakYi DaiKamila M. LudwikowskaYi SunKamila Pokorska-NiewiadaYi ZhangKamila R. IrvineYi-Chen HsiehKammi B. GuntonYichi YangKamyar AsadipooyaYi-Chin FanKang Min ParkYichun ChenKannan RamarYie Hou LeeKannan SridharanYifat SnirKanokwan SanchaisuriyaYihao ChenKanon JatuworaprukYi-Heng LiKanthee AnantapongYi-Hsing ChenKaoruko ShimizuYi-Jou TaiKapil Kiran AedmaYi-Ju ChenKapil SirohiYi-Jung LiKara K. BrockhausYik Ling ChewKara L. HollowayYiliang KuoKaran SinglaYinan JiangKarel KostevYin-Chih FuKarel KotaškaYing GaoKaren A. SullivanYing JiangKaren BannertYing QingKaren BohmwaldYing-Chun ChienKaren JohnstoneYingJu LaiKaren K. BallenYingjui LinKaren L. RechYingleong Rigel ChanKaren L. SternYingli YangKaren MolyneuxYing-Shuo HsuKaren Villar ZarraYingting CaoKaren WinerYingWei FanKarin ConinxYin-Hwa ShihKarin Ekström SmedbyYinzhi LangKarin MalíčkováYishay SzekelyKarin PfisterYitang SunKarina KapczukYi-Ting LinKarina LezirovitzYiu Wing KamKarina Wierzbowska-DrabikYixiang DengKarine PanicoYize I. WanKarl Anders KnutssonYoann BirlingKarl AndriessenYoav ArnsonKarl Guenter NoéYoav NahumKarl Henrik GrinnemoYochai BirnbaumKarl Peter IttnerYoga YuniadiKarol Alí Apaza AlccayhuamanYogananda S. MarkandeyaKarol ČurilaYohan KerbageKarol Kaziród-WolskiYohannes W. WoldeamanuelKarol KonaszewskiYohei HayashiKarol NowakYohei IkezumiKarol Rawicz-PruszyńskiYohei KitamuraKarol SzylukYohei KomaruKarolina BonińskaYohei OkadaKarolina ChilickaYoichi KobayashiKarolina ŁuczkowskaYoichi MatsuoKarolina NoworytaYoichi WatanabeKarolina Walczyńska-DragonYojiro KatoKarsten GroteYoko FukuharaKarsten LenkYoko HoshiKarthick SubramanianYoko OzawaKarthik TappaYolanda Castellote-CaballeroKarthikeyan MuthusamyYolanda FortenberryKasha Priya SinghYoncheong WongKasi VegesanaYong ChenKasper SalinYong DuKassem SharifYong Il HwangKatalin PetőYong Ju JangKatanasaka YasufumiYong Koo KangKatarina BjörsesYong TangKatarina SebekovaYong-Beom ParkKatarina Steding-EhrenborgYongbo ZhengKatarzyna A. LisowskaYong-Chan KimKatarzyna AkutkoYongfen XuKatarzyna ArkuszYong-Seog OhKatarzyna Bąk-DrabikYongsheng RuanKatarzyna BanachYong-Soo KwonKatarzyna Beata CywkaYongwook JungKatarzyna BłochowiakYongxing YaoKatarzyna DziendzikowskaYoon Young KimKatarzyna FischerYoon-Goo KangKatarzyna GrzelaYoon-Ji KimKatarzyna GurzawskaYoon-Seob KimKatarzyna HolcmanYoon-Soo KimKatarzyna HolubYoo-Ri ChungKatarzyna KotfisYorihisa KitagawaKatarzyna Kotulska-JóźwiakYorito HattoriKatarzyna KrysikYoshiaki MizutaniKatarzyna KrzywickaYoshiaki ZaizenKatarzyna LeszczyńskaYoshifumi IkedaKatarzyna LewandowskaYoshifumi SugitaKatarzyna LomperYoshiharu KawaguchiKatarzyna Mańka-MalaraYoshihiko FurutaKatarzyna Michalska-MałeckaYoshihiko UsuiKatarzyna Mocny-PachońskaYoshihiro FukumotoKatarzyna NeubauerYoshihiro KamadaKatarzyna NowomiejskaYoshihiro NoguchiKatarzyna Ogrodzka-CiechanowiczYoshihiro TanakaKatarzyna PankiewiczYoshihiro TangeKatarzyna PawlakYoshihisa MatsukawaKatarzyna Plagens-RotmanYoshihito ShimaKatarzyna PogodaYoshikatsu NomuraKatarzyna Ptaszynska-KopczynskaYoshikazu Honda-OkuboKatarzyna RobaszkiewiczYoshikazu KishinoKatarzyna SędłakYoshiko KidaKatarzyna SkrypnikYoshiko MatsudaKatarzyna ŚmiłowskaYoshimasa HachisuKatarzyna WigluszYoshinori UchidaKatarzyna ZdanowiczYoshio YamaokaKate CheneyYoshiro HoraiKate HimmelmannYoshiro MatsuiKaterina GrafanakiYoshiteru MorioKaterina JirsovaYosra A. HelmyKateřina KaňkováYosra FalfoulKaterina KomrskovaYosuke HirakawaKatharina AlthausYosuke MatsuuraKatharina Antonia DrerupYosuke NakazawaKatharina FeilYosuke SekiKatharina HanselYouakim SalibaKatharina RumpYouichi YasuiKatherine A. A. ClarkYoulim KimKatherine A. Kaproth-JoslinYoun Joung ChoKatherine A. McCrackenYounes ZaidKatherine Ann McAllisterYoung Suk KwonKathleen A. R. SchildrothYoung-Hee NamKathleen M. AntonyYoung-Hoon LeeKathleen NichollsYoung-Keun OnKathrin Julia WeidnerYoun-Jee ChungKathrin WagnerYousef Ahmed FouadKathrin WelckerYousif SubhiKathryn AppletonYoussef LatifehKathryn Browning-CarmoYozo IshiujiKathryn Jean WichtYu Jin LimKátia Nunes SáYu KangKatia OrvinYu Ri WooKatia StefanantoniYu SawadaKatja MohorcicYu SolovievaKatja SchladitzYu SunKatrin KoflerYu TakahashiKatrin RauenYu TaniguchiKatrina LambertYuan GaoKatrine Lawaetz KristensenYuan YuanKatsuhiro ItoYuan-Chieh LeeKatsuhisa OhgiYuan-Hung WangKatsuko KikuchiYuanhung WuKatsunori MatsuedaYuan-Hwa ChouKatsunori YoshidaYuan-Sheng TzengKatsushi TakebayashiYuan-Ta LiKatsuya KitamuraYuan-Ti LeeKatsuya SakaiYuan-Yuan WangKaviraja UdupaYubbu PutriKawsar AhmedYu-Chih ChenKayvan ZainabadiYu-Chih WangKazimierz KarolewskiYu-Ching ChouKazimierz KuliczkowskiYudai YamazakiKazuaki OyakeYuedan WangKazuhide OhtaYuen-Ki CheongKazuhiko HashimotoYu-Feng HuKazuhiko TakeuchiYugo IwayaKazuhiro IwadohYuhei MatsudaKazuhiro KimuraYuh-Mou SueKazuhiro MiyataYuhree KimKazuhiro OmuraYu-Hsuan Joni ShaoKazuhito SuzukiYu-Hung ChenKazuki HiraoYuichi GotoKazuki M. MatsudaYuichi HoriKazuki UedaYuichi NishiokaKazuki YasudaYuichi SaitoKazuko Kaneda-NakashimaYuichi TakanoKazumasa MatsumotoYuichiro ShirotaKazunao HayashiYuichiro WatanabeKazunori TobinoYuichiro YoneokaKazuo KawasugiYuji HigashimotoKazuo TanneYuji KamijoKazutaka AonumaYuji KasukawaKazuya TateishiYuji NagatomoKazuyoshi NakanishiYuji ShimizuKazuyuki HirookaYuji UchioKei EndoYujiro NishiokaKei SaitoYu-Jung ChengKei YamadaYuka I. SumitaKei YamasakiYuka OtakiKeigi FujiwaraYukari Aoki‐NonakaKeigo UchimuraYuki HashimotoKeiichi KubotaYuki KataokaKeiichi MatsubaraYuki KomukuKeiichiro MukaiYuki NishiKeiji ShinozukaYuki YoshimatsuKeiko MaedaYukichi TokitaKeiko YamamotoYukihide NishimuraKeisuke EguchiYukihiro SanadaKeisuke MurakamiYukihiro WadaKeisuke SuzukiYukiko NakaharaKeitaro KanieYukinori OzakiKeith SiauYukio KobayashiKeld-Erik BygYukio SekiKelly ChiquetoYuko NakagawaKelly Cristina StéfaniYuko OnoKelly DomvriYuko OyaKelly E. JohnsonYuko Waguri-NagayaKelly Lisette TillemanYula SerpanosKelly PicardYuli FradkinKelsey GabelYulia GendlerKelvin Ian AfrashtehfarYulia O. BobrovaKemal GörürYu-Lin WangKemal ÖrnekYuliya I. RaginoKen MillsYu-Lung HsuKen SakaiYuma FuseKengo FuruichiYuman LiKengo TakahashiYumiko IshizawaKenichi GotoYumiko KanzakiKenichi SamejimaYu-Min HuangKenichi YamadaYun FengKen-Ichiro KonishiYun Jin KimKenichiro MotomuraYun WangKenji HayashidaYundi FengKenji IharaYung-Hao TsouKenji YamagataYungi KimKenneth A. AndreoniYung-Shun JuanKenneth A. EarleYung-Shuo KaoKenneth A. EllenbogenYung-Taek OuhKenneth G. LindenYung-Tsu ChoKenneth HungYung-Wei ChiKenneth MoiseYu-Ning HuKenneth N. WhiteYunkoo KangKenneth OoiYunsong LiuKenneth R. KnechtYupei LiKenneth R. McLeishYuqing ZhangKenneth VitaleYuri BattagliaKenneth WalshYuri GanushchakKenneth Wayne AltmanYuriy L. Vasil’evKennio Ferreira-PaimYuriy V. FlominKenny Yat Hong KwanYurong LiangKensuke KumeYusef MoullaKensuke MatsushitaYu-Sheng YangKensuke UraguchiYushi SuzukiKent D. CarlsonYusuf SertKenta FurutaniYusuf Ziya ŞenerKenta MurotaniYusuke AraiKentaro OkazakiYusuke EndoKentaro TojoYusuke HashimotoKentaro WakamatsuYusuke IsshikiKenya KamimuraYusuke KatayamaKenzaburo NakajimaYusuke KuritaKeren Cohen-HagaiYusuke NakagawaKeren GrinbergYusuke OsawaKerr GrahamYusuke ShimizuKerstin Alexandra KlotzYuta MarukiKerstin JunkerYuta TerabeKetil StørdalYutaka EguchiKeunBaDa SonYutaka IgarashiKeun-Yeong JeongYutaka MifuneKevin ChangYutang WangKevin ChenYutaro KandaKevin HackshawYuval KriegerKevin J. LandYu-Wei LiuKevin J. WhiteheadYuxia MaKevin L. GarvinYuya WatanabeKevin LobdellYvan MartineauKevin M. KliftoYvelynne P. KellyKevin RoedlYves BorbélyKevin RolfeYves PietteKevin WillyYves SabanKeyla Vargas-RománYvonne A. EfeberaKeyue DingYvonne SingerKeyvan AghababaiyanZ. BergerKezhou ZhuZ. Ozlem SoranKhadija BanuZ. YusofKhaldoon AljerianZacarías Sánchez MiláKhaldoon O. Al-NosairyZachary BurtonKhaled El MatriZafar NeyazKhaled EmaraZaffer QasimKhaled SinjabZaher BahouthKhalid AlmasZahi I. MitriKhalid MasoodZahra AshenaKhalid Shaker IbrahimZahra BagheriKhalid SiddiquiZahra HooshyariKhan HekmatyarZahra MalekiKhashayar SakhaeeZaid Al-QurayshiKhayal GasimliZaid Bin MahbubKhristine Joy C. AntiguaZaid YounesKhrystyna O. SemenZaki HaidariKi KimZakir KhanKi Wha ChungŻaneta Kimber-TrojnarKiani KiandokhtZarenezhad ElhamKibum KimZarrin BasharatKichang LeeZbigniew AdamczewskiKi-Il LeeZbigniew GarncarekKijin KimZbigniew JaroszewiczKikuo IsodaZbigniew RaszewskiKil-Soo KimZbigniew SerafinKim LawsonZbigniew ZyliczKim Van HoorenbeeckZbyněk StraňákKimberly JasmerZdenek ZadakKimberly S. ToppZdravka PoljakovičKimi SatoZdravko PetanjekKinga Grzech-LeśniakZeba FarooquiKinga MusiałZeenat IqbalKingman StrohlZeev OrmianerKingston RajiahZekiye KaraçamKira AstakhovaZeliha GulerKiran ReddyŽeljka Kačarević PerićKiran SandhuZeljka MinicKirat K. ChandŽeljka Večerić-HalerKiril M. StoyanovŽeljko ReinerKiritkumar PatelZeljko ZivanovicKirsi M. IsoherranenZenon MariakKirsten CouplandZenon PogorelićKirsten Marie JochumsenZeynep Alpay SavasanKirstin VachZeynep B. ZenginKishor PantZhan ShuKishore BalasubramanianZhao WangKishore BharathyZhao YangKishu RanjanZhaohui BaiKitamura MineakiZhaozhong ZhuKi-Won MoonZhemeng WuKiyoshi AsadaZhen QiuKiyoshi YaoedaZheng Zachory WeiKiyotaka UchiyamaZheng ZeKjersti JohnsrudZhenhong HeKlaas F. FranzenZheqi LiKlaudia BanachZhi ChaiKlaudia DopytalskaZhibo MaKlaus BrusgaardZhichao WuKlaus DresingZhihao ChenKlaus Edgar RothZhihuang ZhengKlaus MatschkeZhijun LiuKlaus PetersZhijun ZhouKlearchos PsychogiosZhipeng CaoKlemen SteblovnikZhipeng HuKlemens BuddeZhiping FengKnaizer BorisZhirong YangKnierim JanZhiyong HouKo Eun KimZhong WangKo HashimotoZhongbing LuKodjovi D. MlagaZhongheng ZhangKoen M. E. M. ReyntjensZhongmin JinKoga YasuhikoZhongwei HuangKohei NozakiZhongxing ZhangKohei OkuyamaZhou WeidongKohei ShigetaZhubin J. GahvariKoichi AndoZhuqiu JinKoichiro KinugawaZia HashimKoji MitaZiad KhoueirKoji OtaniZiad Said DahdouhKoji YamatsuZichen JiKojiro HyodoZiga SnojKok Hian TanZikai HaoKok Siong ChenZili XieKollarigowda RavichandranŽilvinas SaladžinskasKomandoor SrivathsanZisman DevyKonarski WojciechŽivka DikaKondapa Naidu BobbaZoann NugentKonlawij TrongtrakulZoe RutherfordKonosuke NakajiZofia CzosnykaKonrad AumannZofia T. BilińskaKonrad FutymaZoka MilanKonrad Leszek MendralaZoltan HeroldKonrad StępieńZoltán JáraiKonstantin Christoph KobanZoltán RakonczayKonstantin Johannes ScholzZoltan RuzsaKonstantin KudrinZongli RenKonstantin L. UttingerZongzhi YinKonstantin SchwarzZoran TodorovićKonstantinos A. GatzoulisZorana DobrijevićKonstantinos DitsiosZoubir DjeradaKonstantinos DroutsasZoya V. SurninaKonstantinos G. KyriakoulisZozan GulekenKonstantinos KatogiannisZrinka MihaljevićKonstantinos KypriotisZsofia DohyKonstantinos MargetisZsolt NemethKonstantinos N. PaterakisZsolt PiróthKonstantinos PapadopoulosZsolt VajdaKonstantinos PappelisZsuzsa Máthéné SzigetiKonstantinos PerivoliotisZsuzsanna ArányiKonstantinos PetsiosZsuzsanna OroszKonstantinos S. MylonasZsuzsanna RecsanKonstantinos SeretisZuhair NattoKonstantinos SidiropoulosZul’Fiya Sagdullaevna KhodzhaevaKonstantinos SpanosZülkif TanrıverdiKonstantinos StamatiouZully PedrozoKonstantinos StergiosZulvikar Syambani UlhaqKonstantinos TriantafylliasZurabi LominadzeKonstantinos TriantafyllouZuzana VišňovcováKonstanty SzułdrzyńskiZuzanna NowakKoray DurmazZyanya Reyes-CastilloKornanong YuenyongchaiwatZygfryd Witkiewicz

